# Decreased adipokine CTRP4 in CAD patients: CTRP4 attenuates atherosclerosis via inhibition of RAGE and TLR4

**DOI:** 10.1002/ctm2.70624

**Published:** 2026-02-18

**Authors:** Xinyi Shu, Feifei Li, Jiawei Chen, Xinrui Wu, Leyuan Tao, Abulikemu Amuti, Shuai Chen, Jinwei Quan, Jingmeng Liu, Yipaerguli Maimati, Fenghua Ding, Ying Shen, Qiujing Chen, Weifeng Shen, Ruiyan Zhang, Yang Dai, Xiaoqun Wang, Lin Lu

**Affiliations:** ^1^ Department of Cardiovascular Medicine, Rui Jin Hospital Shanghai Jiao Tong University School of Medicine Shanghai P.R. China; ^2^ Institute of Cardiovascular Diseases Shanghai Jiao Tong University School of Medicine Shanghai P.R. China; ^3^ National Research Center for Translational Medicine, Rui Jin Hospital Shanghai Jiao Tong University School of Medicine Shanghai P.R. China

**Keywords:** atherosclerosis, CTRP4, RAGE, TLR4

## Abstract

**Background:**

C1q/TNF‐related proteins (CTRPs) belong to the adipokine family. Here, we aimed to assess the relation of CTRP4 levels in serum and perivascular adipose tissue (PVAT) with coronary artery disease (CAD), and investigate the effect of CTRP4 on atherosclerosis and the underlying mechanisms.

**Methods:**

CTRP4 levels were examined in serum and epicardial adipose tissue (a major PVAT) from patients with CAD. Atherosclerotic lesions were analysed in CTRP4^‒/‒^/ApoE^‒/‒^ and ApoE^‒/‒^ mice. The paracrine effects of CTRP4 on atherosclerosis were tested by supplementation with CTRP4, either via PVAT transplantation or tail vein injection in CTRP4^−/−^/ApoE^−/−^ mice. CTRP4‐interacting proteins were identified using immunoprecipitation and mass spectrometry.

**Results:**

CTRP4 levels were lower in serum and epicardial adipose tissue of patients with CAD compared to non‐CAD controls. CTRP4 knockout promoted atherosclerosis in ApoE^‒/‒^ mice. Supplementation of CTRP4, but not receptor for advanced glycation end‐products (RAGE)‐ and toll‐like receptor 4 (TLR4)‐binding incompetent CTRP4 mutant, either through adipose tissue transplantation from wild‐type mice or intravenous injection of recombinant protein, attenuated atherosclerosis in CTRP4^‒/‒^/ApoE^‒/‒^ mice. In macrophages, CTRP4 protein, but not the mutant, suppressed the expression of lipopolysaccharide‐induced inflammatory cytokines. Mechanistically, the anti‐atherogenic effects of CTRP4 were mediated by the engagement and inhibition of RAGE and TLR4.

**Conclusions:**

Decreased CTRP4 levels in serum and epicardial adipose tissue are associated with CAD in patients. CTRP4 deficiency promotes the development of atherosclerosis in ApoE^‒/‒^ mice, whereas CTRP4 supplementation attenuates atherosclerosis via binding and inhibition of RAGE and TLR4. These results suggest that CTRP4 is a novel anti‐inflammatory and anti‐atherogenic adipokine inversely associated with CAD and a potential therapeutic target.

## Graphical abstract

1. CTRP4 is a key endogenous inhibitor of vascular inflammation and atherogenesis. Deficiency of CTRP4 promotes atherosclerosis in ApoE^‒/‒^ mice.

2. CTRP4 supplementation either via PVAT transplantation or injection of recombinant protein antagonise atherosclerosis in ApoE^‒/‒^ mice.

3. The anti‐atherogenic effect of CTRP4 is mediated through RAGE and TLR4 engagement and inhibition.

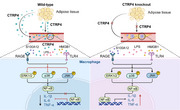

## 1 INTRODUCTION

Adipokines play an important role in the development of atherosclerosis.^1^ In metabolic disturbance, visceral adipose tissue secrets pro‐inflammatory adipokines to influence the vascular wall in an ‘inside‐out’ way or by an ‘outside‐in’ mechanism exerted from inflamed perivascular adipose tissue (PVAT) during the pathogenesis of atherosclerosis.^2^, ^3^

C1q/TNF‐related proteins (CTRPs) belong to the adipokine family. These proteins form homo‐ and heterotypic trimers of oligomers with different biological functions.^4^ Previous studies have shown that the CTRP family members are implicated in the regulation of different pathophysiological stages of atherosclerosis. CTRP3, CTRP9, CTRP12, CTRP13 and CTRP15 play protective roles against atherosclerosis, whereas CTRP1, CTRP5 and CTRP7 exert pro‐atherosclerotic effect.^5^, ^6^, ^7^, ^8^ Moreover, CTRP3 inhibits lipopolysaccharide (LPS)‐induced systemic inflammation and ERK‐1/‐2 phosphorylation in adipose tissue in mice.^9^ CTRP3 also suppresses toll‐like receptor (TLR)‐mediated expression of cathelicidin antimicrobial peptide in adipocytes.^10^

The receptors for the major CTRPs are unclear, and some are redundantly targeted by several CTRPs. The mechanism by which CTRPs activate their receptors and influence downstream signalling pathways is largely unknown.

Recently, information regarding the relationship of CTRP4 with cardiovascular diseases has been reported. In a cross‐sectional study, serum CTRP4 concentrations were negatively associated with carotid atherosclerotic plaques in patients with T2DM.^11^ Moreover, CTRP4 reduces inflammation by limiting endothelial‒monocyte interactions via the SIRT6/Nrf2 pathway.^12^ In this study, we showed that CTRP4 levels were decreased in serum of coronary artery disease (CAD) patients than in that from non‐CAD subjects, and in epicardial adipose tissue surrounding atherosclerotic coronary arteries from CAD patients compared with that surrounding non‐atherosclerotic coronary arteries. CTRP4^−/−^/ApoE^−/−^ mice exhibited increased atherosclerosis compared with ApoE^−/−^ mice. Administration of recombinant CTRP4 protein inhibited atherogenesis in ApoE^−/−^ mice. Moreover, adipose tissue transplantation from wild‐type mice but not CTRP4 mutant‐expressing mice onto the carotid arteries of CTRP4^−/−^/ApoE^−/−^ mice attenuated partial ligation‐induced atherogenesis. Mechanistically, CTRP4 exerted anti‐inflammatory and anti‐atherogenic effects through the engaging and inhibiting receptor for advanced glycation end‐products (RAGE) and TLR4 signalling pathways.

## 2 METHODS

### 2.1 Human samples

All procedures were performed under the ethical standards of the Declaration of Helsinki (2013), with approval obtained from the Institutional Review Committee of Shanghai Jiao Tong University (approval no. RJ‐CAMPUS‐2022177). Written informed consent was provided by all participants before enrollment.

Diagnostic coronary angiography and, when indicated, percutaneous coronary intervention (PCI) were performed via radial or femoral access using standard techniques. After intracoronary nitrate administration, angiographic images were subsequently analysed by semi‐automated quantitative coronary angiography using edge‐detection software (QAngio XA, Medis) by two experienced cardiologists blinded to clinical and biochemical information. CAD was defined as at least one major coronary artery stenosis ≥50%.^13^

From January 2023 to September 2023, 556 consecutive symptom‐driven patients with CAD underwent PCI in Ruijin Hospital affiliated to Shanghai Jiao Tong University School of Medicine. For research, individuals with prior coronary artery bypass grafting (CABG) (*n* = 35), acute coronary syndrome (*n* = 102), malignant tumours or immune system disorders (*n* = 19), renal insufficiency requiring haemodialysis (*n* = 18) and heart failure with NYHA class III or IV (*n* = 59) were excluded. Overall, 323 patients with CAD were included in this study for serum CTRP4 measurement. This study also enrolled 315 healthy controls undergoing physical check‐ups with no established diagnosis and evidence of cardiovascular diseases during examination (Table S1 and Figure S1).

Epicardial adipose tissue surrounding coronary arteries with severe atherosclerosis in CAD patients (undergoing CABG) and non‐atherosclerotic arteries in non‐CAD patients receiving other cardiac surgeries (including valve replacement or repair, heart transplantation) (*n* = 9) were obtained. Among these samples, three per group were utilised for RNA sequencing (RNA‐seq), and nine samples per group were employed for Western blot and immunofluorescence staining analyses.

### 2.2 Animal studies

All experimental procedures involving animals received ethical approval from the Animal Care Committee of Shanghai Jiao Tong University (approval no. RJ2023027) and were carried out in line with the National Institutes of Health (NIH) guidelines for the Care and Use of Laboratory Animals.

Using a CRISPR/Cas9 genome‐editing approach, CTRP4 knockout (CTRP4^‒/‒^) mice were established (Shanghai Biomodel Organism Science and Technology Development Co., Ltd.). The target site was selected within the coding region of exon 2 of CTRP4‐001, and a highly efficient gRNA was designed and screened, followed by in vitro transcription of gRNA and Cas9 mRNA. During microinjection, gRNA and Cas9 mRNA were injected into C57BL/6 fertilised eggs, generating F0 founder mice. Under gRNA guidance, Cas9 nuclease introduced frameshift mutations or premature stop codons, resulting in functional gene loss. Founder (F0) genotypes were validated by PCR and Sanger sequencing. Verified founders were backcrossed onto a C57BL/6 background to establish F1 heterozygotes, and homozygous CTRP4^‒/‒^ mice were subsequently derived. To generate CTRP4 and apolipoprotein E (ApoE) double knockout (CTRP4^−/−^/ApoE^−/−^) mice, homozygous CTRP4^−/−^ mice were crossed with ApoE^−/−^ mice of the C57BL/6 strain. CTRP4^+/+^/ApoE^−/−^ littermates were used as controls.

To investigate the effects of recombinant CTRP4, 8‐week‐old male CTRP4^‒/‒^/ApoE^‒/‒^ or ApoE^‒/‒^ mice were administered with recombinant CTRP4 or CTRP4^Mut^ (10 µg/mouse, every other day), or saline, via tail vein injection, and atherosclerosis was induced by feeding a Western diet for 12 weeks. To further examine whether PVAT‐secreted CTRP4 exerts anti‐atherogenic effects through a paracrine pathway, adeno‐associated viruses overexpressing wild‐type CTRP4 (AAV‐CTRP4), CTRP4^Mut^ (AAV‐CTRP4^Mut^) or control vector (AAV‐Vector) were administered intraperitoneally to male CTRP4^‒/‒^ mice (6‒8 weeks old) at a dose of 5 × 10^11^ vector genomes in a 100 µL volume of sterile saline for 2 weeks. Mesenteric adipose tissue (MAT) harvested from donor mice was grafted perivascularly onto the carotid arteries of male CTRP4^‒/‒^/ApoE^‒/‒^ recipients at 8 weeks of age; after transplantation, recipients were maintained on a Western diet for 14 days to induce carotid atherosclerosis.

The mice were housed at the Animal Experiment Center of Rui Jin Hospital, Shanghai Jiao Tong University, under pathogen‐free conditions, where they were provided unlimited access to food and water, and exposed to a 12‐h light/dark regimen. For diet‐induced atherosclerosis, male mice were maintained on standard chow until 8 weeks of age and then switched to 12‐week Western diet (40% fat, 1.25% cholesterol; D12109C, Research Diets). In transplantation experiments, recipient mice were kept on a 2‐week Western diet after grafting.

### 2.3 Cell culture

Bone marrow‐derived macrophages (BMDMs) were prepared following an established procedure.^14^ Briefly, bone marrow cells were harvested from the long bones of 6‐week‐old CTRP4^−/−^ and wild‐type mice, and differentiated in complete Roswell Park Memorial Institute (RPMI) 1640 medium containing 10% foetal bovine serum (FBS) and 1% penicillin/streptomycin, with macrophage‐colony stimulating factor added at 50 ng/mL in 5% CO_2_ at 37°C for 48 h.

To determine whether CTRP4 alleviates inflammation, BMDMs from CTRP4^−/−^ mice were pretreated with recombinant CTRP4 protein for 3 h, and exposed to inflammatory stimuli, such as LPS. To further investigate the functional domains of CTRP4 responsible for its anti‐inflammatory effects, BMDMs were pretreated for 3 h with CTRP4, CTRP4^D2^ or CTRP4^Mut^ proteins (1 µg/mL), and subsequently exposed to LPS (50 ng/mL), HMGB1 (5 µg/mL) or S100A12 (50 µg/mL) at indicated time (0, 20 and 40 min).

To determine whether RAGE, TLR4 and interleukin‐6 alpha‐receptor (IL‐6Rα) are involved in CTRP4‐mediated anti‐inflammatory effects, BMDMs from CTRP4^−/−^ mice were transfected with siRNA‐RAGE (si RAGE), siRNA‐TLR4 (si TLR4) or siRNA‐IL‐6Rα (si IL‐6Rα), with or without pretreatment with recombinant CTRP4 protein (1 µg/mL, 3 h), and then exposed to LPS (50 ng/mL) for 20 min. Subsequently, the activation of inflammation signalling pathways was measured.

Human undifferentiated THP‐1 monocytes and 3T3‐L1 pre‐adipocyte cell line were obtained from Ethephon Biotechnology Co., Ltd. human THP‐1 monocytic leukemia cells (THP‐1) cells were grown in RPMI 1640 medium supplemented with 10% FBS and 1% penicillin/streptomycin. To induce differentiation, THP‐1 cells were treated with phorbol 12‐myristate 13‐acetate (100 ng/mL) for 48 h.^15^

The 3T3‐L1 cells were maintained in Dulbecco's modified Eagle's media (DMEM) containing 10% newborn calf serum (NCS) and 1% penicillin/streptomycin. Two days after reaching confluence (defined as day 0), preadipocytes were induced to differentiate by replacing the medium with DMEM/10% NCS containing 5 µg/mL insulin, .5 mM 3‐isobutyl‐1‐methylxanthine, .5 µM dexamethasone and 2 µM rosiglitazone for 48 h. Cells were subsequently maintained in DMEM/10% NCS with insulin (1 µg/mL) until days 6‒8, with medium changes every 2 days.^16^

To determine whether pro‐atherogenic stimuli modulate CTRP4 expression in macrophages and adipocytes, BMDMs from wild‐type mice and differentiated 3T3‐L1 adipocytes were incubated for 24 h with oxidised low‐density lipoprotein (ox‐LDL; 100 µg/mL), IL‐6 (50 ng/mL), LPS (50 ng/mL), tumour necrosis factor‐alpha (TNF‐α; 50 ng/mL) or interferon‐γ (IFN‐γ; 50 ng/mL).

The HEK293 cells were sourced from the Classic Specimen Culture and Storage Center, Wuhan University and maintained in minimum essential medium containing 10% FBS, 1% non‐essential amino acids and penicillin/streptomycin in 5% CO_2_ at 37°C.

### 2.4 Blood sample measurements

Following an overnight fast, blood samples were collected from all participants (323 patients with CAD and 315 control subjects). Serum was separated immediately and stored at −80°C to minimise diurnal variations in CTRP4 levels. Serum CTRP4 was determined with a commercially available enzyme‐linked immunosorbent assay kit (SK00084; Avisera Bioscience Inc.) according to its instructions.

### 2.5 Plasmid construction, siRNA, recombinant adeno‐associated virus and recombinant protein generation

To ascertain CTRP4 function and verify the interaction of CTRP4 with RAGE and TLR4, human full‐length cDNA of CTRP4, RAGE and TLR4 was subcloned into the mammalian expression vector pcDNA3.1 (+) to generate pcDNA3.1‐CTRP4‐6×His (His‐CTRP4), pcDNA3.1‐RAGE‐3×Flag (Flag‐RAGE) and pcDNA3.1‐TLR4‐3×Flag (Flag‐TLR4) by OBiO Technology. To further determine the regions of CTRP4 that interact with RAGE and TLR4, the following mutant plasmids were constructed, including pcDNA3.1‐CTRP4‐D1 (25‐162)‐6×His (His‐CTRP4^D1^), pcDNA3.1‐CTRP4 ‐D2 (172‐317)‐6×His (His‐CTRP4^D2^) and pcDNA3.1‐CTRP4 (R69A, R71A, R98A and Y128A)‐6×His (His‐CTRP4^Mut^).

The recombinant adeno‐associated virus vector in serotype 8 capsids was constructed to encode murine CTRP4 (AAV8‐CTRP4) and CTRP4^Mut^ (AAV8‐CTRP4^Mut^) by OBiO Technology. The expression of CTRP4 and CTRP4^Mut^ was mediated by AAV serotype AAVRec2, which efficiently targets adipose tissue.

To test the interaction of CTRP4 or CTRP4 mutant with RAGE and TLR4, recombinant proteins of His‐CTRP4, His‐CTRP4^D2^ or His‐CTRP4^Mut^ were generated as described previously.^17^ Briefly, CTRP4, CTRP4^D2^ or CTRP4^Mut^ proteins, fused with a His‐tag, were expressed in HEK293 cells. Five days post‐transfection, cells were harvested and solubilised in extraction buffer (50 mM Tris, 150 mM NaCl, 8 M urea, 20 mM imidazole). His‐tagged proteins were purified by Ni‐IDA chromatography, captured on Ni‐agarose beads, and eluted with imidazole. The eluates were further polished by reverse‐phase high‐performance liquid chromatography on a SOURCE 15 RPC column (GE Healthcare). Purity was verified using SDS‒PAGE and Western blot, and the final preparations were stored in phosphate‐buffered saline (PBS) (pH 7.4). Endotoxin levels were confirmed to be <1 EU/µg.

### 2.6 Western blot analysis

Protein levels in tissues and cultured cells were quantified using Western blot analysis.^13^ Briefly, lysates were separated by SDS‒PAGE and transferred onto polyvinylidene fluoride membranes (IPVH00010; Millipore). The membranes were then blocked with either 5% nonfat milk or 5% BSA (for phospho‐protein detection). Following blocking, primary antibodies were applied overnight at 4°C. After washing, the membranes were incubated with secondary antibodies conjugated to HRP for 1 h at room temperature. Protein signals were detected using an Imaging System (Tanon 4200, Tanon) and enhanced chemiluminescence. Quantification was performed using ImageJ software (NIH), with normalisation to β‐tubulin (for tissue samples) or GAPDH (for cell samples). The antibodies used are detailed in the Key Resources Table.

### 2.7 Co‐immunoprecipitation assays

Co‐immunoprecipitation (Co‐IP) assays were conducted following previous study.^18^ HEK293 cells were co‐transfected with His‐tagged CTRP expression plasmids (full‐length CTRP4, mutant CTRP4, CTRP1 or CTRP3) in combination with Flag‐tagged RAGE or TLR4. After 36 h, proteins were extracted from cells and quantified. Each sample (300 µg) was rotated overnight at 4°C in the presence of anti‐His and anti‐Flag antibodies. The bead‒antibody‒antigen complexes were separated using a magnetic separator and washed three times on ice with immunoprecipitation lysis buffer. The antigen was eluted with 2× SDS sample buffer (40 µL) at 100°C for 10 min, followed by SDS‒PAGE.

### 2.8 Adipose tissue transplantation

Adipose tissue transplantation was conducted as detailed previously.^19^, ^20^ All recipient mice were maintained under inhalational anaesthesia with 1.5% isoflurane delivered in oxygen at .5 L/min. Forty milligrams of MAT was obtained from CTRP4^‒/‒^ mice or wild‐type controls (6‒8 weeks old). The adipose tissue was loop over the carotid artery after the removal of endogenous PVAT. Mice were maintained on a Western diet for 14 days after surgery. On day 14 post‐transplantation, the mice were euthanised by cervical dislocation under deep isoflurane anaesthesia (3%), and the carotid arteries and transplanted adipose tissues were collected for further analysis.

A partial ligation model was established in the left carotid artery, with or without transplantation. The mice underwent perivascular collar placement after deep anaesthesia with an intraperitoneal injection of pentobarbital sodium. The external carotid, internal carotid, and occipital artery (branches of LCA) were ligated with 6‐0 silk suture, while the superior thyroid artery was kept patent.^21^ Throughout the experiment, the animals were monitored regularly for signs of infection or distress, and their health status was supported with unrestricted access to food and water.

### 2.9 Histological and immunofluorescence analysis

Histochemistry and immunofluorescence staining were conducted as outlined in previous studies.^18^ To evaluate the atherosclerotic lesions in the entire aorta, the dissected aorta was fixed in 4% paraformaldehyde and longitudinally opened from the ascending aorta to the iliac artery bifurcation. En face analysis was performed using Oil Red O staining, and the lesion area was quantified using Image‐Pro Plus 6.0 (Media Cybernetics) as a percentage of the total aortic area. For aortic root assessment, 8‐µm serial frozen sections were collected, and lesions were examined in five independent sections, each spaced 80 µm apart.^22^ Plaque composition was characterised by haematoxylin and eosin (H&E) staining; lipid deposition area (mm^2^) was visualised using Oil Red O staining, while collagen content (percentage of lesion area) and necrotic core size (µm) were assessed using Masson's trichrome staining.

Immunofluorescence staining was performed on human and mouse tissues that were fixed in 4% paraformaldehyde, followed by preparation of 5‐µm paraffin sections. After antigen retrieval in sodium citrate buffer (10 mM, pH 6.0), sections were permeabilised with .2% Triton X‐100 and blocked with 10% goat serum followed by overnight incubation at 4°C with anti‐CTRP4 (1:100) and anti‐macrophages/monocytes (MOMA‐2) (1:100) antibodies. Alexa Fluor‐conjugated secondary antibodies (1:1000) were applied for 1 h at room temperature, nuclei were counterstained with DAPI. Images were acquired on a fluorescence microscope (BX53, Olympus), and quantitative analysis was conducted with Image‐Pro Plus 6.0 software (Media Cybernetics). Quantification of CTRP4 was represented as the fluorescence intensity of CTRP4 per image, and quantification of MOMA‐2 positivity was expressed as the MOMA‐2^+^ area normalised to the total lesion area.

### 2.10 Analysis of single‐nucleus RNA sequencing dataset

Single‐nucleus RNA sequencing (snRNA‐seq) dataset of human PVAT (GSE166355, *n* = 3) was retrieved from the NCBI Gene Expression Omnibus.^23^ Data were analysed in R using Seurat (v5.3.0; SeuratObject v5.1.0).^24^ After quality control based on standard metrics (nFeature_RNA, nCount_RNA and percent.mt), data were log‐normalised, variable features were selected, and dimensionality reduction was initiated by principal component analysis. Harmony embeddings were applied to correct for batch effects. (uniform manifold approximation and projection) UMAP was used to visualise the cell clusters, which were further annotated based on marker genes. UMAP plots were used to display cluster structure, dot plots to summarise marker expression across annotated cell types, and violin plots to show the distribution of C1QTNF4 (encoding CTRP4) expression in major populations.

### 2.11 Bulk RNA‐seq

Bulk RNA‐seq was conducted as described previously.^25^ To analyse transcriptomic changes in human and mouse adipose tissues, total RNA was extracted from epicardial adipose tissue surrounding atherosclerotic coronary arteries and non‐atherosclerotic coronary arteries (*n* = 3 per group), as well as from the subcutaneous and visceral adipose tissue of CTRP4^‒/‒^ and wild‐type mice (*n* = 3 per group), using TRIzol Reagent. RNA quality control included NanoDrop 2000‐based quantification (Thermo Fisher Scientific) and RNA integrity profiling on an Agilent 2100 Bioanalyser (Agilent Technologies). Library preparation and NovaSeq 6000 sequencing were outsourced to OE Biotech (Shanghai). FASTQ data were cleaned with FASTP by removing adapters and low‐quality bases/reads prior to downstream analyses. Differential gene expression was analysed using DESeq2, applying a significance threshold of adjusted *p* < .05 and a |log2 fold change| > 1. Visualisation of these genes was carried out in R with ggplot2 (volcano plots) and pheatmap (heatmaps), respectively.

### 2.12 Surface plasmon resonance

Surface plasmon resonance (SPR) was performed following the method outlined in previous studies.^26^, ^27^ To investigate the interaction between CTRP4 and RAGE or TLR4, SPR analysis was performed on a Biacore 8K system (Cytiva, GE Healthcare) using a CM5 sensor chip (29149603; Cytiva). Recombinant wild‐type or CTRP4 mutant proteins were immobilised onto the chip surface via amine coupling, in line with the manufacturer's instructions. Briefly, the surface of the CM5 chip was activated with N‐ethyl‐N′‐(dimethylaminopropyl) carbodiimide and N‐hydroxysuccinimide to form reactive ester groups. The CTRP4 protein (at an appropriate concentration) was then injected, allowing it to bind covalently to the chip surface via the amine groups. The unreacted sites were blocked with ethanolamine to prevent non‐specific binding.

To detect the interaction with RAGE or TLR4, recombinant RAGE or TLR4 proteins in serial dilutions were flowed over the CTRP4‐immobilised surface at a rate of 30 µL/min. The binding responses were monitored in real‐time, and the association and dissociation kinetics were recorded. The binding affinity (*K*_D_) between CTRP4 and RAGE or TLR4 was determined using Biacore 8K Evaluation software (version 3.0.12).

Control experiments were conducted using a blank flow cell on the CM5 chip to account for non‐specific binding. The interactions were evaluated by calculating the equilibrium dissociation constant, which was derived from the ratio of the association and dissociation rates.

### 2.13 Mass spectrometry analysis

THP‐1 cells were stimulated with LPS (50 ng/mL) for 12 h, and the cell lysate was incubated with the lysate of HEK293 cells overexpressing His‐CTRP4. Immunoprecipitated His‐CTRP4 samples were obtained using an anti‐His antibody. Protein bands were excised and subjected to trypsin digestion, followed by analysis of the extracted peptides using liquid chromatography‒tandem mass spectrometry (LC‒MS/MS) on an Orbitrap Elite mass spectrometer (Thermo Fisher Scientific). Data analysis was performed using the MaxQuant software, and the identified proteins were annotated using UniProt database. Mass spectrometry analyses, including gel extraction, sample loading, data acquisition and protein identification, were performed by Genechem Co., Ltd.

### 2.14 Molecular docking

Molecular docking analysis was performed following the previous study.^28^ The 3D structures of CTRP4 (AF‐Q9BXJ3‐F1), CTRP1(AF‐Q9BXJ1‐F1), CTRP3 (AF‐Q9BXJ4‐F1), RAGE (AF‐Q9BXJ4‐F1) and TLR4 (AF‐O00206‐F1) proteins were retrieved from the AlphaFold Protein Structure Database (https://alphafold.com/). Protein structures were prepared by removing water molecules and heteroatoms using PyMOL. Docking simulations were conducted using AutoDock Vina to explore the binding interactions between CTRPs and RAGE or TLR4. The docking grid was set around the potential binding sites, and poses were evaluated by predicted binding energies and key interaction contacts.

### 2.15 Statistical analysis

Data are shown as mean ± standard deviation. Statistical analysis was performed using GraphPad Prism 8 (GraphPad Software). The distribution of the data was assessed for normality with the Shapiro‒Wilk test. For parametric data, two‐group comparisons used unpaired Student's *t* test (two‐tailed), whereas comparisons across multiple groups used one‐way ANOVA with Bonferroni correction for post hoc testing. For data that deviated from normality, nonparametric tests were employed: the Mann‒Whitney *U*‐test for two‐group comparisons and the Kruskal‒Wallis test with Dunn's multiple‐comparison procedure for analyses of three or more groups. Binomial logistic regression was constructed to identify the independent determinants of CAD. The variables included age, sex, body mass index, hypertension, glycaemic status, high‐sensitivity C‐reactive protein and CTRP4 levels. All analyses used two‐sided tests with an overall significant level of *α* = .05 except for special indication.

A priori power analysis was performed in PASS v15 (NCSS, LLC) to determine the required sample size for two‐group comparisons, based on a two‐sample *t*‐test assuming equal variances (10% standard deviation, 80% power and 5% type I error rate) for two‐group comparisons. One‐way ANOVA was utilised to compare more than two groups, with independent groups analysed separately. For measurements taken from multiple tissues within the same animal, one‐way repeated measures ANOVA was employed. In all cases, the statistical power requirements were deemed achievable with a minimum of three animals per group.

## 3 RESULTS

### 3.1 Decreased levels of CTRP4 in serum and epicardial adipose tissue of CAD patients

**FIGURE 1:**
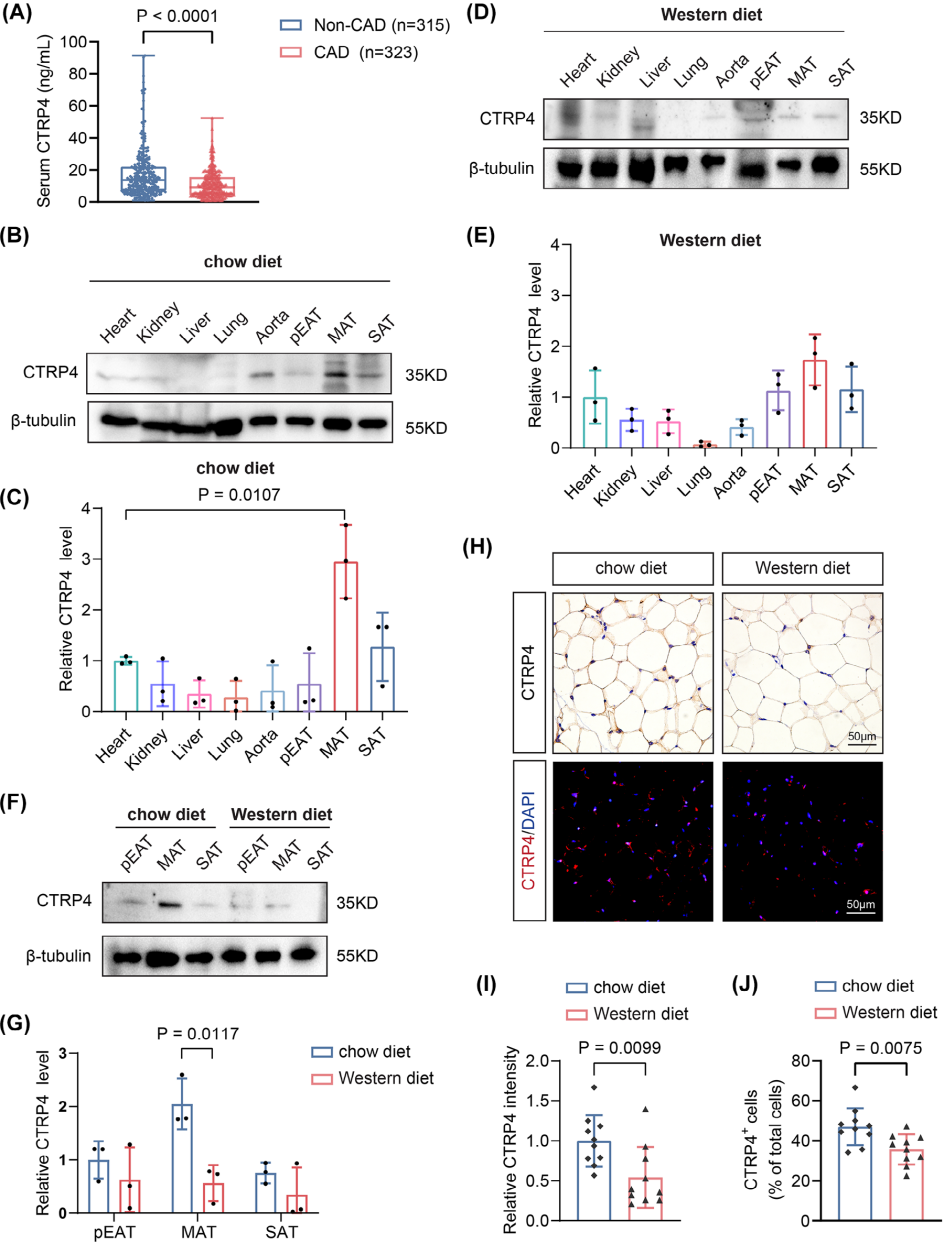
Reduction of C1q/TNF‐related protein 4 (CTRP4) level in the serum of patients with coronary artery disease (CAD), and in adipose tissue of mice with high‐fat feeding. (A) Serum CTRP4 levels in patients with CAD (*n* = 323) and non‐CAD controls (*n* = 315) were detected using enzyme‐linked immunosorbent assay (ELISA). (B) CTRP4 protein levels in the heart, kidney, liver, lung and adipose tissue of C57BL/6 mice (*n* = 3) fed a chow diet were detected by Western blot. (C) Quantification of the data in (B) (*n* = 3). (D) CTRP4 protein levels in the heart, kidney, liver, lung and adipose tissue of C57BL/6 mice (*n* = 3) fed a Western diet were detected by Western blot. (E) Quantification of the data in (D) (*n* = 3). (F) Western blot was used to detect the levels of CTRP4 protein in the para‐epididymal adipose tissue (pEAT), mesenteric adipose tissue (MAT) and subcutaneous adipose tissue (SAT) of C57BL/6 mice fed a chow diet and those fed a Western diet (*n* = 3 per group). (G) Quantification of the data in (F) (*n* = 3). (H) Immunohistochemical staining was used to observe the levels of CTRP4 MAT of C57BL/6 mice fed a chow diet and those a Western diet. Scale bar, 50 µm. (I) Quantification of CTRP4 protein levels in (H) (*n* = 10). (J) Quantitative analysis of immunofluorescence staining in (H) showing the proportion of CTRP4‐positive cells in adipose tissue (*n* = 10). Data are presented as mean ± SD. Data in (A) were analysed using Mann‒Whitney *U*‐tests. Data in (C) and (E) were analysed using one‐way ANOVA followed by Bonferroni post hoc tests. Data in (G), (I) and (J) were analysed using unpaired Student's *t*‐test.

To investigate the association between CTRP4 and atherosclerosis, we evaluated the serum CTRP4 levels in patients with CAD and healthy controls without CAD. The demographic characteristics and parameters of all participants were presented in Table S1. Notably, CAD patients had lower serum CTRP4 levels than non‐CAD controls (*p* < .001) (Figure 1A). Moreover, CTRP4 levels were inversely correlated with the Gensini score (Spearman's *r* = ‒.381, 95% confidence interval [CI]: ‒.473 to ‒.281, *p* < .001) and SYNTAX score (Spearman's *r* = ‒.257, 95% CI: ‒.359 to ‒.149, *p* < .001) (Figure S2A,B). We further classified the patients with CAD into three subgroups according to the number of diseased coronary arteries, using non‐CAD controls as 0‐vessel disease, and categorised the patients into four subgroups according to quartiles of the Gensini or SYNTAX scores. CTRP4 levels persistently associated with CAD severity (Figure S2C‒E).

Multivariable logistic regression analysis (Table S2) demonstrated a consistent association between lower CTRP4 levels and the presence of CAD across all models: unadjusted (model 1), adjusted for age and sex (model 2), and fully adjusted for clinical characteristics (model 3). In the fully adjusted model, individuals with serum CTRP4 levels <12.22 ng/mL (cutoff from receiver operating characteristic curve [ROC] analysis) had a significantly higher likelihood of CAD (odds ratio = 2.20, 95% CI: 1.56‒3.11, *p* < .001) compared to those with levels ≥12.22 ng/mL. ROC analysis yielded an area under the curve (AUC) of .638 (95% CI: .596‒.681, *p *< .001) for CTRP4 in predicting CAD, with an optimal cutoff point of 12.22 ng/mL (sensitivity = .644 and specificity = .562). In addition, inclusion of CTRP4 on top of conventional risk factors effectively improved the predictive performance of the regression models (Figure S3).

Next, in C57BL/6 mice, we examined the expression of the CTRP4 protein in different organs and in different adipose depots. The results showed that in mice fed with chow diet, CTRP4 was enriched in the MAT and lower expression was observed in the heart, liver, kidney, lung, skeletal muscle, aorta, para‐epididymal adipose tissue and subcutaneous adipose tissue (Figure 1B,C). However, CTRP4 levels in MAT were significantly declined in C57BL/6 mice fed with Western diet feeding (Figure 1D‒G). These features were confirmed by immunohistochemistry and immunofluorescence staining (Figure 1H‒J).

**FIGURE 2:**
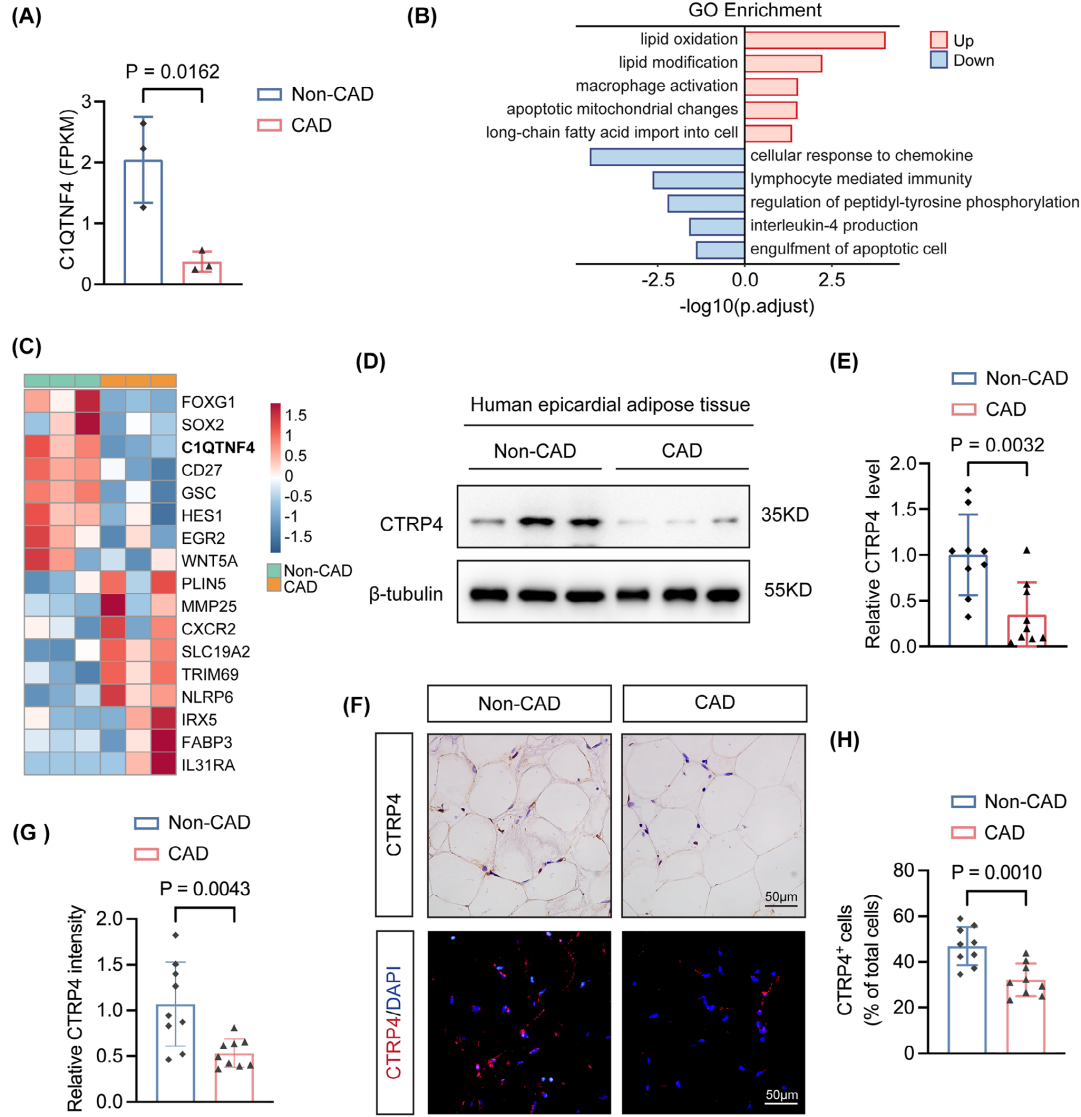
Decreased C1q/TNF‐related protein 4 (CTRP4) levels in epicardial adipose tissue of patients with coronary artery disease (CAD). (A) Transcriptional expression of C1QTNF4 was assessed in human epicardial adipose tissue surrounding atherosclerotic coronary arteries (*n* = 3) and non‐atherosclerotic coronary arteries (*n* = 3) by bulk RNA sequencing. (B) Gene Ontology (GO) enrichment analysis of differentially expressed genes. (C) Heatmap of differentially expressed genes. (D) Western blot was used to detect the expression of CTRP4 in epicardial adipose tissue surrounding atherosclerotic coronary arteries from patients with CAD undergoing coronary artery bypass grafting (CABG) surgery (*n* = 9) and non‐atherosclerotic coronary arteries from non‐CAD patients receiving other cardiac surgery (*n* = 9). (E) Quantification of the data in (D). (F) Immunohistochemical and immunofluorescence staining of CTRP4 in epicardial adipose tissue surrounding atherosclerotic coronary arteries from patients with CAD undergoing CABG surgery (*n* = 9) and non‐atherosclerotic coronary arteries from non‐CAD patients receiving other cardiac surgery (*n* = 9). Scale bar, 50 µm. (G and H) Quantification of CTRP4 protein levels in epicardial adipose tissue using immunohistochemical and immunofluorescence staining in (F). Data are presented as mean ± SD. Data in (A), (E), (G) and (H) were analysed using unpaired Student's *t*‐test.

To ascertain whether CTRP4 is negatively associated with CAD, and to identify the atherosclerosis‐related genes in epicardial adipose tissue, we examined gene expression profiles in epicardial adipose tissue surrounding severe atherosclerotic coronary arteries (*n* = 3) and non‐atherosclerotic coronary arteries (*n* = 3) by RNA‐seq. In total, 1072 genes with differential expression were identified, comprising 678 upregulated and 428 downregulated genes. These genes were associated with pathways involved in lipid oxidation, macrophage activation and cellular response to chemokine, as determined by Gene Ontology (GO) enrichment analysis. Notably, the expression of CTRP4 mRNA in epicardial adipose tissue surrounding atherosclerotic coronary arteries was significantly lower than that adjacent to non‐atherosclerotic arteries (Figure 2A‒C), and this finding was supported by Western blot analysis (Figure 2D,E). Moreover, immunohistochemical staining confirmed that CTRP4 expression was reduced in epicardial adipose tissue adjacent to atherosclerotic arteries compared with the control adipose tissue (*p* < .05) (Figure 2F‒H).

To delineate the cellular sources of CTRP4 and explore stimuli that may drive its downregulation, we first analysed a publicly available snRNA‐seq dataset of human PVAT (GSE166355). CTRP4 transcripts were detected across multiple cell populations, including adipocytes, endothelial cells, fibroblasts, macrophages and other cell types (Figure S4A‒C). Given that adipocytes are the primary endocrine cell type responsible for adipokine production, and that macrophages are key mediators of inflammation and atherosclerosis progression and engage in extensive crosstalk with adipocytes, we focused subsequent experiments on these two cell types. Differentiated 3T3‐L1 adipocytes and BMDMs from wild‐type mice were therefore treated with several pro‐atherogenic stimuli, such as ox‐LDL, LPS, IL‐6, TNF‐α or IFN‐γ. CTRP4 expression was reduced to varying degrees in 3T3‐L1 adipocytes or BMDMs following exposure to ox‐LDL, LPS, IL‐6 or TNF‐α, whereas IFN‐γ induced no appreciable change in CTRP4 levels (Figure S4D‒M).

### 3.2 CTRP4 knockout promotes the development of atherosclerosis in ApoE^‒/‒^ mice

Supplementation of CTRP4 through PVAT transplantation from wild‐type mice or injection of CTRP4 protein via tail vein attenuates atherosclerosis in CTRP4^‒/‒^/ApoE^‒/‒^ mice.

**FIGURE 3:**
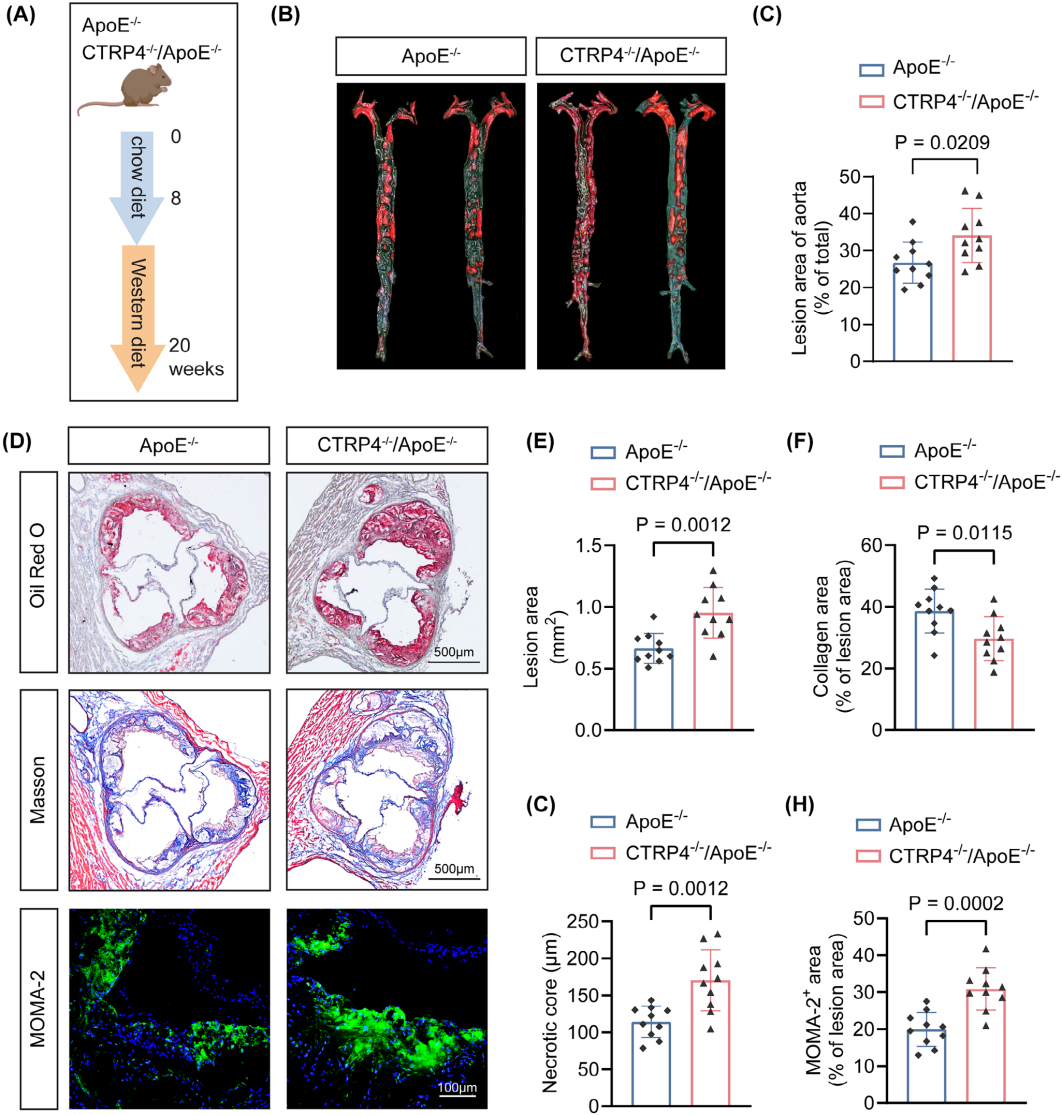
Deficiency of C1q/TNF‐related protein 4 (CTRP4) promotes atherogenesis in ApoE^−/−^ mice. (A) Experimental procedure: ApoE^−/−^ and CTRP4^−/−^/ApoE^−/−^ mice (male) were fed a chow diet for 8 weeks, followed by a Western diet for 12 weeks to establish atherosclerosis models (*n* = 10 per group). (B) En face analysis of aortic plaque area in ApoE^−/−^ and CTRP4^−/−^/ApoE^−/−^ mice. (C) Quantification of the data in (B). (D) Oil Red O, Masson's and macrophages/monocytes (MOMA‐2) staining were performed to evaluate plaque area, collagen content, necrotic core size (scale bar, 500 µm) and macrophage infiltration (scale bar, 100 µm) in the aortic roots of ApoE^−/−^ and CTRP4^−/−^/ApoE^−/−^ mice. (E‒H) Quantification of the data in (D) (*n* = 10 per group). Data are presented as mean ± SD. Data in (C), (E), (F), (G) and (H) were analysed using unpaired Student's *t*‐test. ApoE, apolipoprotein E.

Based on the above results, we investigated whether CTRP4 influenced the development of atherosclerosis. CTRP4^‒/‒^/ApoE^‒/‒^ mice were generated and underwent a 12‐week Western‐diet challenge, alongside ApoE^‒/‒^ control mice (Figure 3A). Notably, CTRP4^‒/‒^/ApoE^‒/‒^ mice showed enhanced atherosclerosis in aorta and aortic roots compared to ApoE^‒/‒^ mice (both *p* < .05) (Figure 3B‒E). Moreover, necrotic core diameter and macrophage infiltration were increased in CTRP4^‒/‒^/ApoE^‒/‒^ mice by Masson's and MOMA‐2 staining analysis (Figure 3D,F‒H). This suggested that CTRP4 is a protective adipokine against atherosclerosis.

**FIGURE 4:**
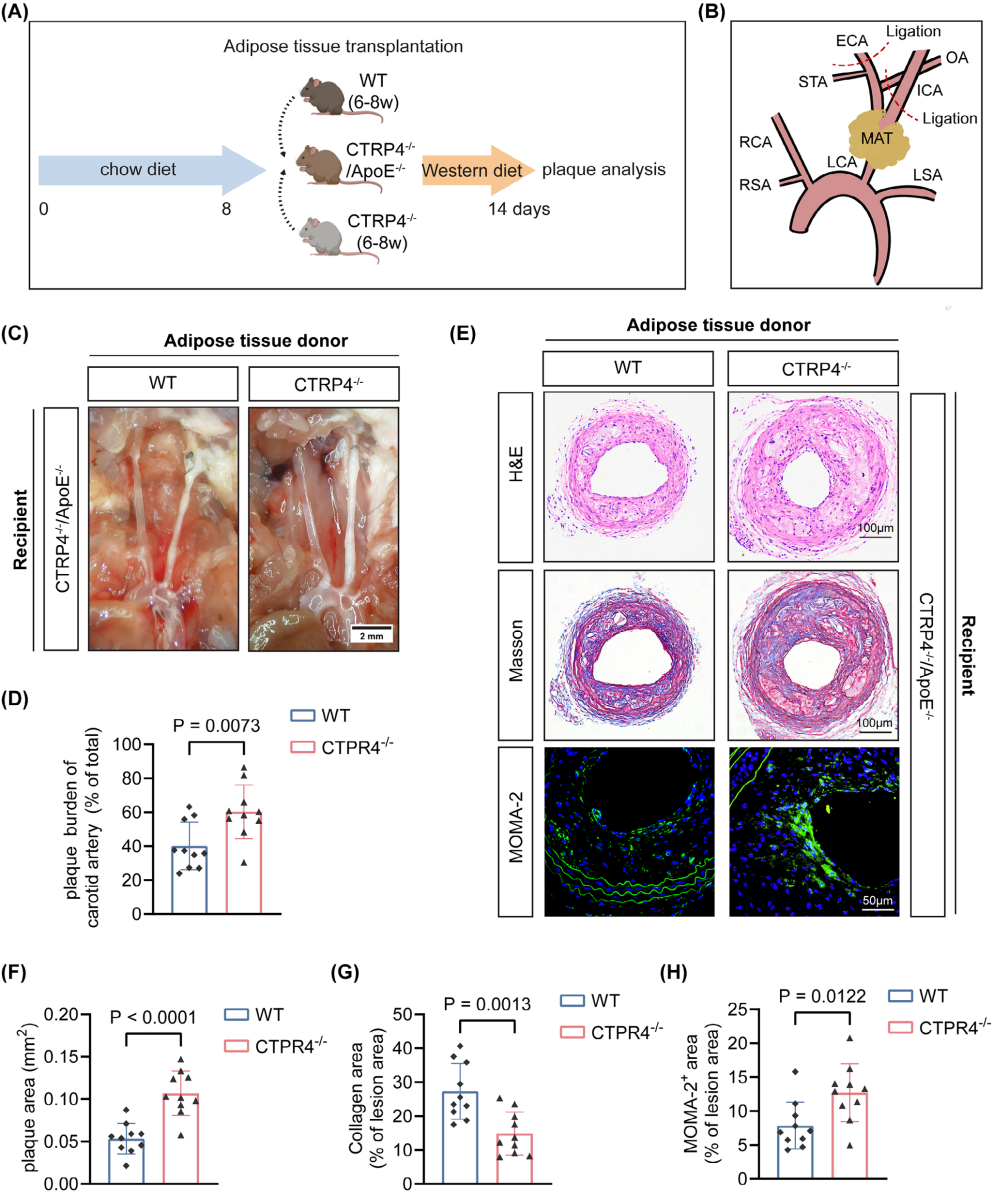
Supplementation of C1q/TNF‐related protein 4 (CTRP4) through perivascular adipose tissue transplantation from wide‐type mice attenuates carotid atherosclerosis in CTRP4^−/−^/ApoE^−/−^ mice. (A) Experimental procedure: CTRP4^−/−^/ApoE^−/−^ mice (male) were fed a chow diet for 8 weeks. Then, mesenteric adipose tissue (MAT) of wild‐type and CTRP4^−/−^ mice (6‒8 weeks old) was transplanted onto the carotid artery of these CTRP4^−/−^/ApoE^−/−^ mice, and Western diet was continued for 14 days to establish the carotid atherosclerosis model (*n* = 10 per group). (B) Schematic diagram of adipose tissue transplantation. (C) MAT from wild‐type and CTRP4^−/−^ mice was transplanted onto the carotid artery of CTRP4^−/−^/ApoE^−/−^ mice, and were fed a Western diet for 14 days. Representative images of carotid artery plaques were shown. Scale bar, 2 mm. (D) Quantification of the data in (C) (*n* = 10 per group). (E) Haematoxylin and eosin (H&E), Masson's (scale bar, 100 µm) and macrophages/monocytes (MOMA‐2) (scale bar, 50 µm) staining of carotid artery plaques in CTRP4^−/−^/ApoE^−/−^ mice after transplantation of MAT from wide‐type and CTRP4^−/−^ mice. (F‒H) Quantification of data in (E) (*n* = 10 per group). Data are presented as mean ± SD. Data in (D), (F), (G) and (H) were analysed using unpaired Student's *t*‐test. ApoE, apolipoprotein E.

To determine the paracrine effect of CTRP4 in PVAT on atherogenesis, we transplanted MAT from C57BL/6 and CTRP4^‒/‒^ mice onto the carotid arteries of CTRP4^‒/‒^/ApoE^‒/‒^ mice followed by feeding the recipients with Western diet for 14 days (Figure 4A,B). Notably, CTRP4^‒/‒^/ApoE^‒/‒^ mice receiving wild‐type adipose tissue transplantation exhibited a lower carotid plaque burden after partial carotid ligation than mice receiving CTRP4‐deficient adipose tissue; however, PVAT transplantation from CTRP4^‒/‒^ to CTRP4^‒/‒^/ApoE^‒/‒^ mice did not have such an effect (Figure 4C,D). Histological analysis of carotid atherosclerosis in the two groups showed that, compared to transplantation of wild‐type adipose tissue, transplantation of CTRP4^‒/‒^ adipose tissue into CTRP4^‒/‒^/ApoE^‒/‒^ mice resulted in increased carotid atherosclerotic plaque areas, decreased collagen content, enlarged necrotic core size and increased macrophage infiltration in plaques (MOMA‐2 positive) (for all comparisons, *p* < .05) (Figure 4E‒H). These data indicate that CTRP4 in PVAT affects the ‘outside‐in’ pathway of atherogenesis and attenuates the development of atherosclerotic plaques in ApoE^−/−^ mice.

**FIGURE 5:**
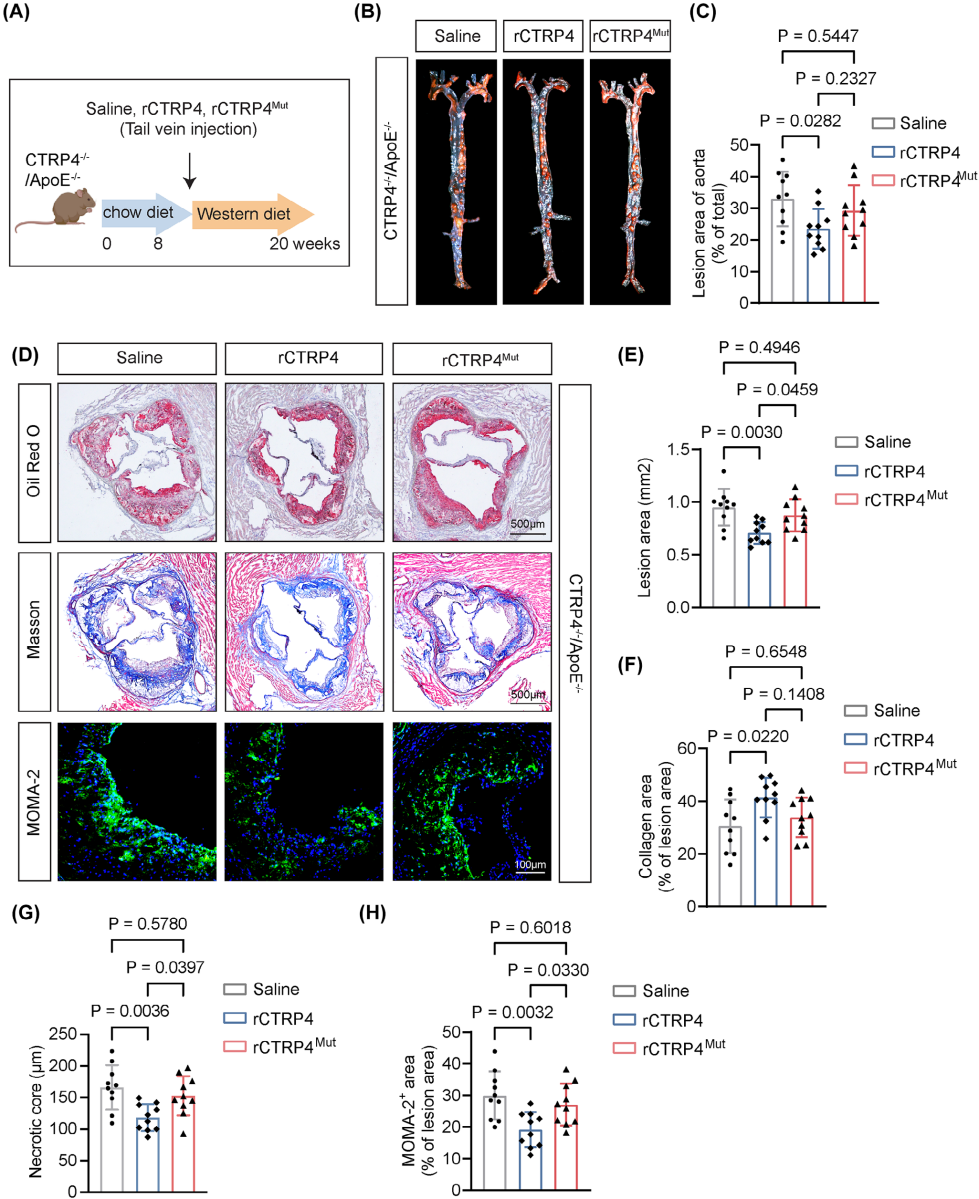
Injection of recombinant C1q/TNF‐related protein 4 (CTRP4) inhibits atherogenesis while its mutant has attenuated anti‐atherogenic effect in CTRP4^−/−^/ApoE^−/−^ mice. (A) Experimental procedure: 8‐week‐old male CTRP4^−/−^/ApoE^−/−^ mice were injected through the tail vein with recombinant protein CTRP4 and CTRP4^Mut^ (10 µg/mouse, once every other day) or saline, and the atherosclerosis models were constructed after 12 weeks of Western diet (*n* = 10 per group). (B) En face analysis of aortic plaque area in CTRP4^−/−^/ApoE^−/−^ mice injected with saline, recombinant CTRP4 or CTRP4^Mut^. (C) Quantification of the data in (B) (*n* = 10 per group). (D) Atherosclerotic plaque area, collagen content, necrotic core diameter (scale bar, 500 µm) and macrophage infiltration (scale bar, 100 µm) were evaluated by Oil Red O, Masson's and macrophages/monocytes (MOMA‐2) staining of aortic roots in CTRP4^−/−^/ApoE^−/−^ mice injected with saline or recombinant proteins CTRP4 and CTRP4^Mut^ (*n* = 10). (E‒H) Quantification of the data in (D) (*n* = 10 per group). Data are presented as mean ± SD. Data in (C), (E), (F), (G) and (H) were analysed by using one‐way ANOVA followed by Bonferroni post hoc tests. ApoE, apolipoprotein E.

Considering that adipokine mainly influence atherogenesis through ‘inside‐out’ pathway from intima, we tested whether intravenous administration of CTRP4 exert a protective effect against atherogenesis in ApoE^−/−^ mice. We injected recombinant CTRP4 protein or saline via tail vein in both CTRP4^−/−^/ApoE^−/−^ and ApoE^‒/‒^ mice, and afterwards, fed them with 12‐week Western diet to establish atherosclerosis models (Figures 5A and S5A). The en face analysis revealed a smaller aortic plaque area in the CTRP4‐treated mice than in saline‐injected controls (Figures 5B,C and S5B,D), with a consistent decrease in the atherosclerotic area in aortic roots (Figures 5D,E and S5C,E). Moreover, Masson's staining showed an increase in collagen amount and a decrease in the size of necrotic core in the plaques of mice treated with CTRP4 (Figures 5D,F,G and S5C,F,G). Reduced macrophage infiltration in the plaques was also observed in these mice, indicating decreased inflammation following CTRP4 administration (Figure 5D,H).

### 3.3 CTRP4 inhibits LPS‐triggered inflammatory cytokine induction in macrophages in vitro

**FIGURE 6:**
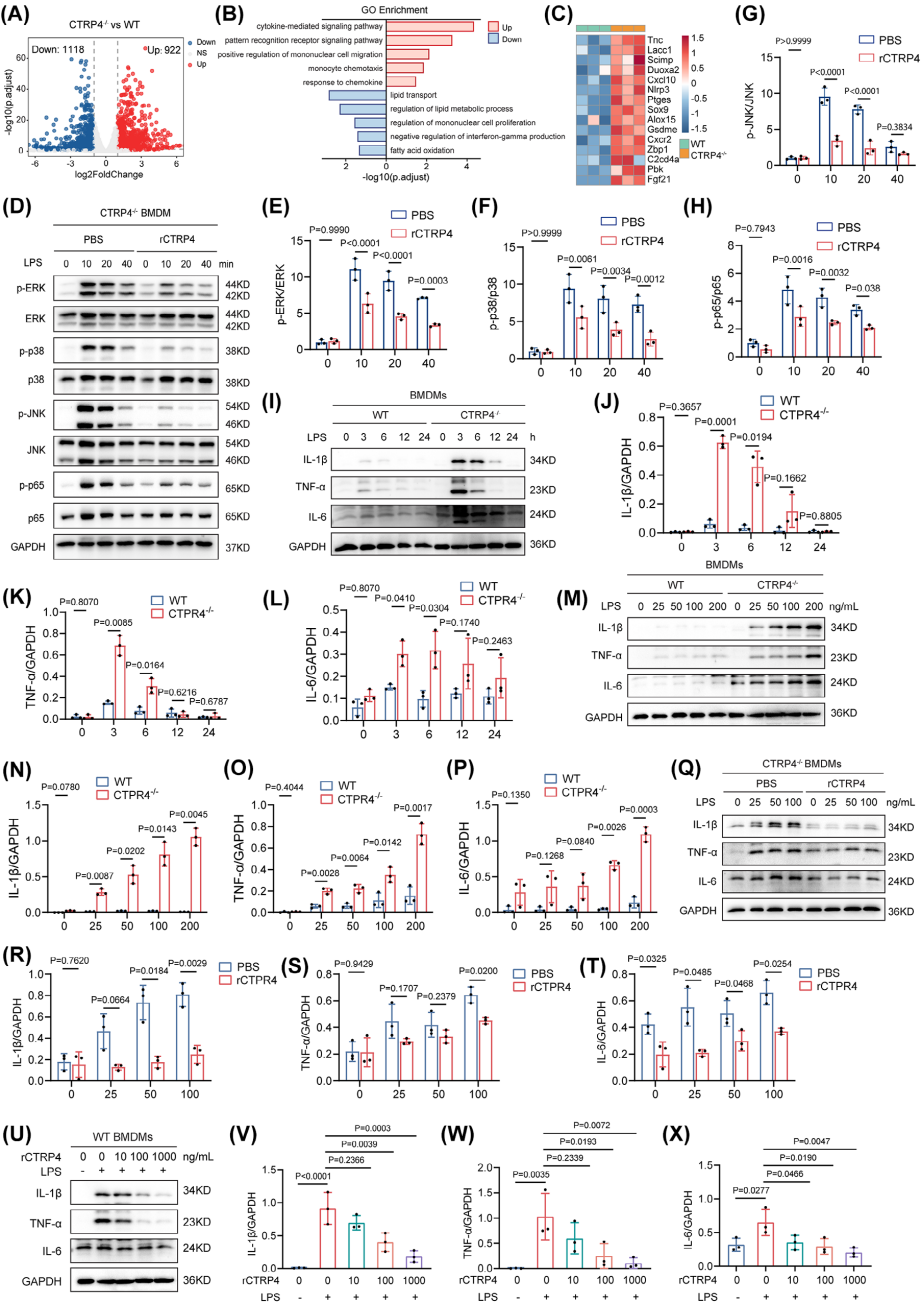
C1q/TNF‐related protein 4 (CTRP4) inhibits inflammatory pathways in macrophages. (A) Volcano plot of differentially expressed genes in bulk RNA sequencing of mesenteric adipose tissue (MAT) of wide‐type (*n* = 3) and CTRP4^−/−^ mice (*n* = 3). Adjusted *p*‐value < .05, and |log2(fold change)| > 1. (B) Gene Ontology (GO) enrichment analysis of differentially expressed genes. (C) Heatmap of differentially expressed genes in inflammatory pathways. (D) Bone marrow‐derived macrophages (BMDMs) of CTRP4^−/−^ mice were treated with phosphate‐buffered saline (PBS) or CTRP4 recombinant protein (1 µg/mL) for 3 h, and then stimulated with lipopolysaccharide (LPS) (50 ng/mL) for 0, 10, 20 and 40 min, respectively. The phosphorylation levels of ERK, p38, JNK and p65 were detected by Western blot. (E‒H) Quantification of data in (D) (*n* = 3). (I) BMDMs from CTRP4^−/−^ and wild‐type (WT) mice were treated with LPS (50 ng/mL) for 0, 3, 6, 12 and 24 h. Western blot was used to detect the levels of inflammatory factors interleukin (IL)‐1β, tumour necrosis factor‐alpha (TNF‐α) and IL‐6. (J‒L) Quantitative analysis of data in (I) (*n* = 3). (M) BMDMs from CTRP4^−/−^ and WT mice were treated with LPS of increasing concentration (25, 50, 100 and 200 ng/mL) for 3 h. Western blot was used to detect the levels of inflammatory factors IL‐1β, TNF‐α and IL‐6. (N‒P) Quantitative analysis of the data in (M) (*n* = 3). (Q) BMDMs from CTRP4^−/−^ mice were pre‐treated with CTRP4 recombinant protein (1 µg/mL) or PBS for 3 h, followed by stimulation with LPS of increasing concentration (25, 50 and 100 ng/mL) for 20 min. Western blot was used to detect the levels of inflammatory factors IL‐1β, TNF‐α and IL‐6. (R‒T) Quantitative analysis of data in (Q) (*n* = 3). (U) BMDMs from WT mice were pre‐treated with rCTRP4 of increasing concentration (0, 10, 100 and 1000 ng/mL) or PBS for 3 h, followed by stimulation with LPS (50 ng/mL) for 20 min. Western blot was used to detect the levels of inflammatory factors IL‐1β, TNF‐α and IL‐6. (V‒X) Quantitative analysis of the data in (E) (*n* = 3). Data are presented as mean ± SD. Data in (E) to (H), (J) to (L), (N) to (P), and (R) to (T) were analysed using unpaired Student's *t*‐test. Data in (V) to (X) were analysed using one‐way ANOVA followed by Bonferroni post hoc tests. p‐ERK, phosphorylated extracellular signal‐regulated kinase; p‐JNK, phosphorylated c‐Jun, N‐terminal kinase; p‐p38, phosphorylated p38 mitogen‐activated protein kinase; p‐p65, phosphorylated p65 subunit of NF‐κB.

We further explored the role and mechanism of CTRP4 in adipose tissue. RNA‐seq analysis of MAT from CTRP4^−/−^ and wild‐type mice identified 922 upregulated and 1118 downregulated genes in the MAT of CTRP4^−/−^ compared with controls (Figure 6A). GO analysis revealed that those enriched genes in adipose tissue of CTRP4^−/−^ mice included cytokine‐mediated signalling pathway, pattern‐recognition receptor signalling pathway, regulation of mononuclear cell migration, monocyte chemotaxis, response to chemokines, lipid transport and lipid metabolic processes (Figure 6B). Inflammation‐related genes were remarkably upregulated and lipid metabolism‐promoting genes were downregulated upon CTRP4 knockout (Figure 6C).

To verify CTRP4 effect on inflammatory cells in vitro, BMDMs from CTRP4^−/−^ mice were exposed to LPS (50 ng/mL) for different time (0, 10, 20 and 40 min) in the presence or absence of recombinant CTRP4 protein. We observed that treatment with CTRP4 attenuated LPS‐induced activation of ERK, p38, JNK and NF‐κB pathways (Figure 6D‒H). Next, BMDMs from wild‐type and CTRP4^−/−^ mice were stimulated with LPS (50 ng/mL) for 0, 3, 6, 12 and 24 h. Western blot revealed that levels of inflammatory cytokine (including IL‐1β, TNF‐α and IL‐6) were significantly higher in BMDMs from CTRP4^−/−^ mice than in those from C57BL/6 mice (for all comparison, *p* < .05) (Figure 6I‒L). Moreover, stimulation with LPS of increasing concentration (0, 25, 50, 100 and 200 ng/mL) greatly upregulated IL‐1β, TNF‐α and IL‐6 levels in CTRP4^−/−^ BMDMs than in wild‐type BMDMs (for all comparison, *p* < .05) (Figure 6M‒P). Furthermore, supplementation of recombinant CTRP4 protein (1 µg/mL) reversed the increased expression of these inflammatory cytokines by LPS in CTRP4^−/−^ BMDMs (for all comparisons, *p* < .05) (Figure 6Q‒T). Recombinant CTRP4 protein reduced the LPS‐induced expression of these inflammatory cytokines in a concentration‐dependent manner (for all comparisons, *p* < .05) (Figure 6U‒X). These data suggested that CTRP4 antagonises inflammation in macrophages.

Since LPS activates TLR4, RAGE, IL‐6R and their downstream pathways,^29^, ^30^ and given prior evidence that CTRP4 influences the pattern‐recognition receptor pathway (Figure 6B), and acts as an endogenous regulator of the IL‐6R‐signalling pathway,^31^ we used siRNA to knockdown the expression of RAGE, TLR4 or IL‐6R in BMDMs, followed by treatment of these cells with LPS and CTRP4 protein. We found that knockdown of RAGE, TLR4 or IL‐6R reduced the levels of phospho‐ERK (phosphorylated extracellular signal‐regulated kinase [p‐ERK]), phospho‐p38 (phosphorylated p38 mitogen‐activated protein kinase [p‐p38]), phospho‐JNK (phosphorylated c‐Jun, N‐terminal kinase [p‐JNK]) and phospho‐p65 (phosphorylated p65 subunit of NF‐κB [p‐p65]), and that the addition of recombinant CTRP4 protein further attenuated the phosphorylation of these proteins in cells treated with siRNA targeting RAGE, TLR4 or IL‐6R, respectively (Figure S6A‒H). These findings indicate a potential anti‐inflammatory role of CTRP4 in macrophages, possibly through antagonising RAGE and TLR4 signalling.

### 3.4 CTRP4 functions through engagement of RAGE and TLR4

**FIGURE 7:**
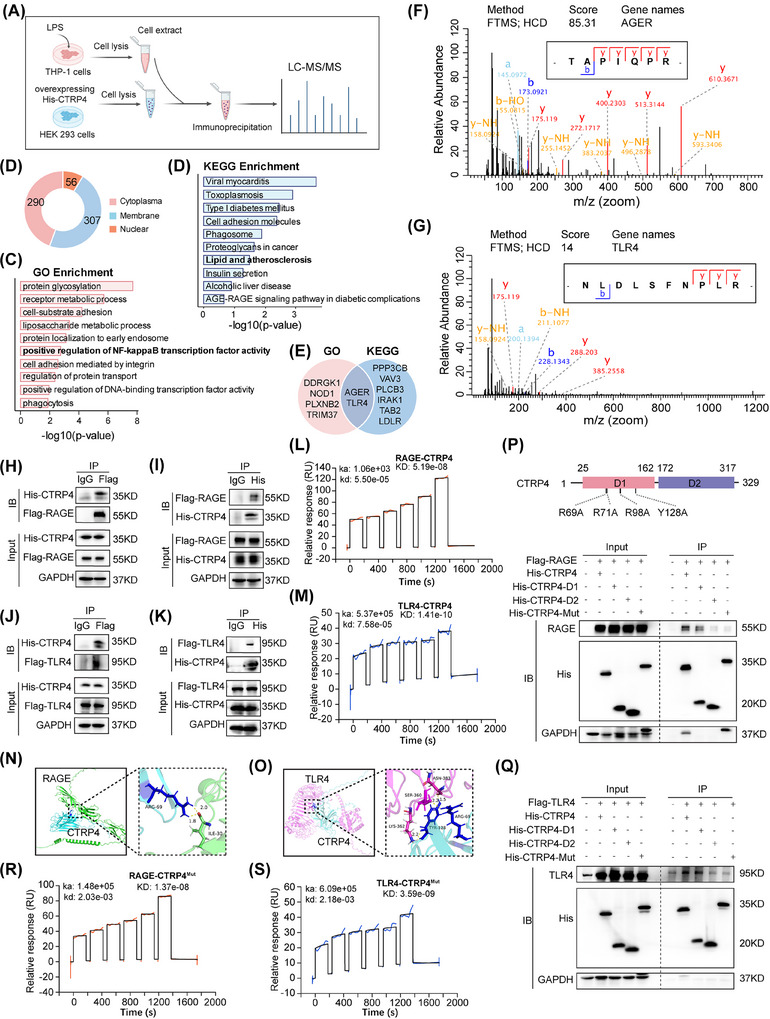
C1q/TNF‐related protein 4 (CTRP4) binds and interacts with receptor for advanced glycation end products (RAGE) and Toll‐like receptor 4 (TLR4). (A) Experimental procedure: HEK293 cells were transfected with His‐CTRP4 overexpression plasmids. After 36 h, the cell lysate was incubated with the extract of lipopolysaccharide (LPS)‐stimulated THP‐1 cells, along with an anti‐His antibody and magnetic beads, to enrich CTRP4. The eluted proteins, including potential CTRP4‐associated proteins, were subsequently analysed using mass spectrometry. (B) Cellular localisation of proteins interacting with CTRP4. Blue indicates membrane proteins, red indicates nuclear proteins, and orange indicates cytoplasmic proteins. (C and D) Gene Ontology (GO) and KEGG enrichment analysis of proteins interacting with CTRP4. (E) Intersection of the genes in the positive regulation of NF‐κB transcription factor activity pathway (GO enrichment) and lipids and atherosclerosis pathway (KEGG enrichment). (F and G) Characteristic peptide spectra of RAGE and TLR4 pulled down by CTRP4. (H‒K) HEK293 cells were co‐transfected with His‐CTRP4 and Flag‐RAGE or Flag‐TLR4, and co‐immunoprecipitation experiments were performed with anti‐Flag or anti‐His antibodies, followed by Western blot analysis. (L and M) Surface plasmon resonance (SPR) analysis of recombinant human CTRP4 binding to RAGE and TLR4. (N and O) Molecular docking predicted the formation of complexes and the binding conformations of CTRP4 to RAGE and TLR4. (P) Flag‐RAGE was co‐transfected with His‐CTRP4 and its mutants His‐CTRP4^D1^, His‐CTRP4^D2^ and His‐CTRP4^Mut^ in HEK293 cells, and immunoprecipitation (IP) experiments were performed using an anti‐His antibody. (Q) Flag‐TLR4 was co‐transfected with His‐CTRP4 and its mutants His‐CTRP4^D1^, His‐CTRP4^D2^ and His‐CTRP4^Mut^ in HEK293 cells, and immunoprecipitation experiments were performed using an anti‐His antibody. (R and S) SPR analysis of recombinant CTRP4 mutant binding to RAGE and TLR4.

To decipher the proteins interacting with CTRP4, THP‐1 macrophages were stimulated with LPS, and the cells lysates were incubated with overexpressed His‐tagged CTRP4 (Figure 7A). We examined CTRP4‐binding proteins using immunoprecipitation and mass spectrometry. The results showed that CTRP4 pulled down 307 membrane proteins (Figure 7B). Proteins interacting with CTRP4 were significantly enriched in protein glycosylation, receptor metabolic process and regulation of NF‐κB activity by GO analysis; and in diabetes, cell adhesion molecules and lipid‒atherosclerosis pathways by (Kyoto Encyclopedia of Genes and Genomes) KEGG analysis (Figure 7C,D). To identify the proteins interacting with CTRP4, we first overlapped the GO‐enriched inflammatory pathway with the KEGG‐enriched atherosclerosis pathway, given their shared involvement in NF‐κB signalling. We then compared the overlapping proteins with peak‐annotated proteins identified in CTRP4 pull‐down assays and revealed RAGE and TLR4 as potential target proteins (Figure 7E‒G). It has been known that RAGE and TLR4 were two membrane receptor proteins closely involved in innate immune reactions.^32^, ^33^, ^34^ Co‐IP analysis revealed that CTRP4 bound to RAGE and TLR4 (Figure 7H‒K). SPR analysis further verified that CTRP4 was interacted with RAGE and TLR4 in a dose‐dependent manner (Figure 7L,M). Molecular docking analysis suggested that CTRP4 had a strong binding affinity for RAGE and TLR4, and formed complexes with these two receptors via multiple hydrogen bonds (Figure 7N,O). To map the CTRP4 region mediating interactions with RAGE and TLR4, two truncated constructs, His‐CTRP4^D1^ (aa 25‒162) and His‐CTRP4^D2^ (aa 172‒317), were generated. In addition, guided by docking‐predicted interface ‘hotspot’ residues within C1q head domain 1, a binding‐deficient point mutant (His‐CTRP4^Mut^) was constructed by alanine substitution of R69, R71, R98 and Y128 (R69A/R71A/R98A/Y128A) to disrupt putative receptor‐contact sites (Figure S7A,B).

Co‐IP assays showed that the binding of CTRP4^D1^ to RAGE and TLR4 was almost comparable to that of the wild‐type CTRP4, whereas CTRP4^D2^ and CTRP4^Mut^ exhibited reduced interactions (Figure 7P,Q). SPR analysis further confirmed the decreased binding of CTRP4^Mut^ to RAGE and TLR4, consistent with the Co‐IP results (Figure 7R,S).

Concurrently, to place CTRP4‐receptor binding in a broader CTRP‐family context, we compared the domain architecture of representative CTRPs and found that CTRP4 uniquely contains two C1q globular head domains. Notably, the predicted interaction sites mapped for CTRP4 binding to RAGE and TLR4 were confined to C1q head domain 1 (D1, aa 25‒162) (Figure S8A), a structural feature that is not shared by the other CTRP members examined and may contribute to the distinct structural and functional properties of CTRP4. To further test whether other atherosclerosis‐related CTRPs engage these receptors, we selected CTRP1 (pro‐atherogenic) and CTRP3 (anti‐atherogenic) as representative family members for validation. Co‐IP assays showed that RAGE physically associated with CTRP1 and CTRP3, while only weak interactions between either CTRP1 or CTRP3 and TLR4 were detected (Figure S8B‒G). Molecular docking predicted potential RAGE‐interacting residues, including K154 and E262/E263 in CTRP1 and N130 and E173 in CTRP3, all of which localised to their respective C1q globular head domains (Figure S8A,H). Together, these data suggest that the C1q head domain represents as a key structural module mediating receptor engagement among CTRP family proteins, while the unique two‐C1q‐head architecture of CTRP4 may underlie its distinct receptor‐interaction profile.

Next, we explored whether the different domains of CTRP4 affected RAGE and TLR4 pathway activities. We used several RAGE and TLR4 ligands (LPS, HMGB1 and S100A12) to induce pathway activation in CTRP4^−/−^ BMDMs with the concomitant addition of wild‐type CTRP4, CTRP4^D2^ and CTRP4^Mut^. The results showed that, as expected, CTRP4 inhibited LPS‐induced increment of p‐ERK, p‐JNK, p38 and NF‐κB activity, with a consistent reduction of inflammatory factor IL‐1β and TNF‐α levels. However, CTRP4^D2^ or CTRP4^Mut^ treatment did not suppress these pathways or inflammatory cytokines production (Figure S9A‒G). Similar results were observed when employing HMGB1 and S100A12 to stimulate CTRP4^−/−^ BMDMs (Figures S10A‒G and S11A‒G). These data indicate that the engagement of CTRP4 with RAGE and TLR4 antagonises the activation of RAGE and TLR4‐related inflammatory signalling, whereas RAGE‐ and TLR4‐binding incompetent CTRP4 mutations reduce its anti‐inflammatory effects.

### 3.5 RAGE‐ and TLR4‐binding incompetent CTRP4 mutant exhibits attenuated anti‐atherogenic effect in vivo

These above results demonstrated that the adipokine CTRP4 can inhibit inflammation and exert anti‐atherogenic effects. Mechanistically, CTRP4 exerts an impact through the engagement of RAGE and TLR4 and inhibits their downstream pathways. Therefore, we tested whether the anti‐atherogenic effect of CTRP4 was dependent on its interaction with these receptors.

**FIGURE 8:**
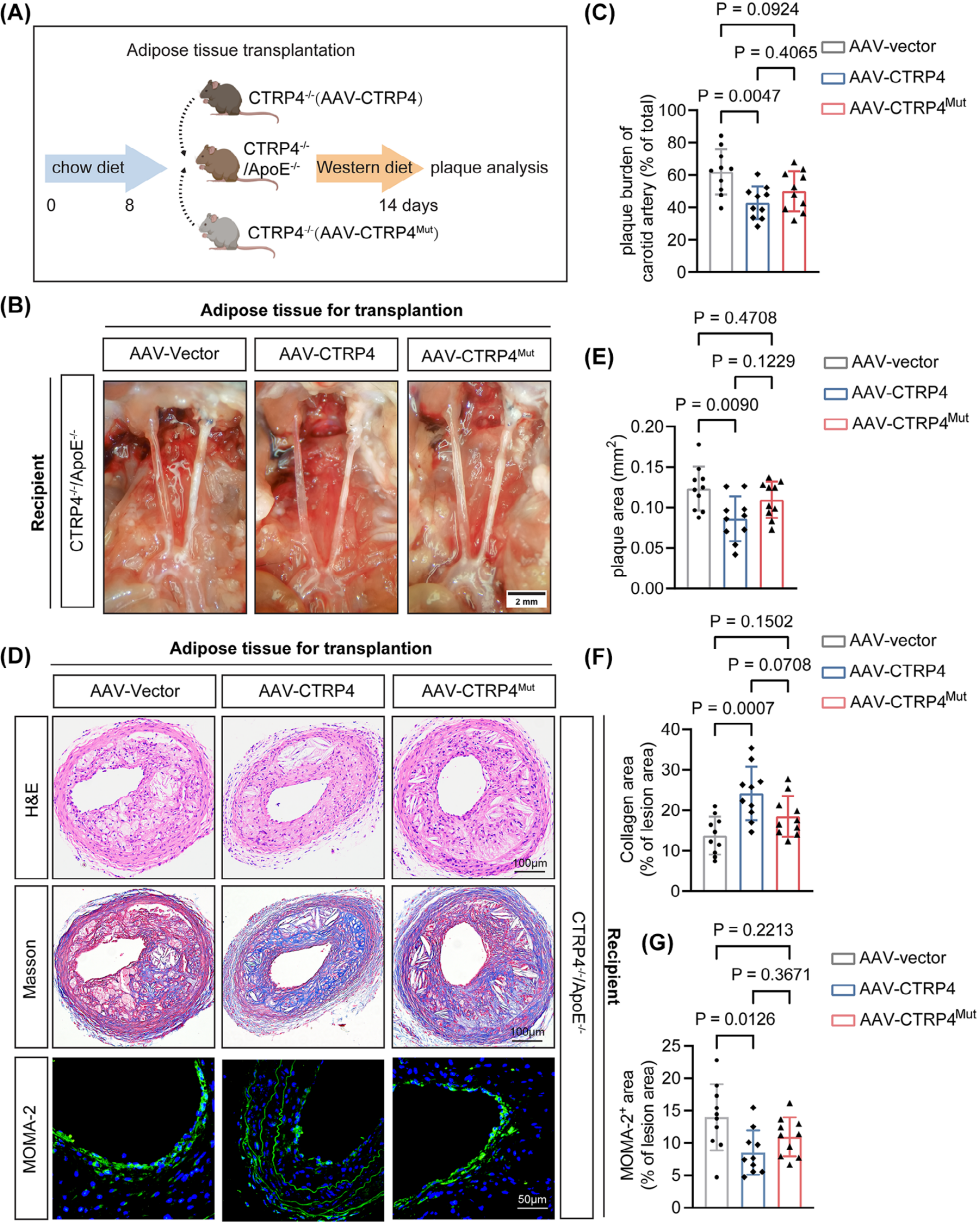
Perivascular adipose tissue transplantation from mice with C1q/TNF‐related protein 4 (CTRP4) mutants fails to inhibit carotid atherosclerosis in CTRP4^−/−^/ApoE^−/−^ mice. (A) Experimental procedure: adeno‐associated viruses overexpressing wild‐type CTRP4 (AAV‐CTRP4) and CTRP4^Mut^ (AAV‐CTRP4^Mut^) and control virus (AAV‐Vector) were injected intraperitoneally into male CTRP4^−/−^ mice (6‒8 weeks old) for 2 weeks. Then, mesenteric adipose tissue (MAT) of these mice was transplanted onto the carotid arteries of 8‐week‐old male CTRP4^−/−^/ApoE^−/−^ mice. Western diet was given for 14 days to induce a carotid atherosclerosis model (*n* = 10 per group). (B) Representative images of carotid atherosclerotic plaques after transplantation of MAT from CTRP4^−/−^ mice infected with AAV‐Vector, AAV‐CTRP4 or AAV‐CTRP4^Mut^ onto the carotid artery of CTRP4^−/−^/ApoE^−/−^ mice. The recipients were fed a Western diet for 14 days. Scale bar, 2 mm. (C) Quantification of the data in (B). (D) Haematoxylin and eosin (H&E), Masson's (scale bar, 100 µm) and macrophages/monocytes (MOMA‐2) (scale bar, 50 µm) staining of carotid atherosclerotic plaques in CTRP4^−/−^/ApoE^−/−^ mice receiving adipose tissue transplantation from CTRP4^−/−^ mice infected with AAV‐vector, AAV‐CTRP4 and AAV‐CTRP4^Mut^. (E‒G) Quantification of the data in (D). Data are presented as mean ± SD. Data in (C), (E), (F) and (G) were analysed using one‐way ANOVA followed by Bonferroni post hoc tests. AAV, adeno‐associated virus; ApoE, apolipoprotein E.

We first examined whether the anti‐atherogenic effect of PVAT‐secreted CTRP4 through paracrine pathway was mediated by the inhibition of RAGE or TLR4. We infected CTRP4^−/−^ mice with adeno‐associated virus overexpressing wild‐type CTRP4, CTRP4^Mut^ or the control virus (AAV‐Vector) via intraperitoneal injection. After 2 weeks, the MAT of these mice was transplanted onto the carotid artery of CTRP4^−/−^/ApoE^−/−^ mice, respectively. The carotid atherosclerotic plaque was analysed after 14 days of Western diet (Figure 8A). Compared to the control group, CTRP4^−/−^/ApoE^−/−^ mice transplanted with adipose tissue overexpressing wild‐type CTRP4 had less carotid artery plaque lesion area, whereas mice transplanted with CTRP4^Mut^‐overexpressing adipose tissue did not show such an effect (Figures S12A,B and 8B,C). Pathological analysis of carotid artery plaques using H&E, Masson's and MOMA‐2 staining revealed that the mice transplanted with wild‐type CTRP4‐overexpressing adipose tissue had reduced plaque thickness, increased collagen content and decreased macrophage infiltration compared with the control group. However, carotid artery plaques of mice transplanted with CTRP4^Mut^‐overexpressing adipose tissue were similar to those of the control groups (Figure 8D‒G).

Next, we tested whether the anti‐atherogenic effect of circulating CTRP4 on ‘inside‐out’ pathway was related to RAGE and TLR4‐inhibiting mechanisms. We injected CTRP4 and CTRP4^Mut^ recombinant proteins via the tail vein of CTRP4^−/−^/ApoE^−/−^ mice, followed by a Western diet was given for 12 weeks to establish atherosclerosis models (Figure 5A). Unlike CTRP4, CTRP4^Mut^ failed to confer a detectable reduction in plaque burden or enhance collagen content (Figure 5B‒F). Moreover, plaques from the CTRP4^Mut^ group exhibited larger necrotic cores and more macrophage infiltration (Figure 5D,G,H), supporting a specific, receptor‐dependent mechanism of CTRP4 action.

The above results indicate that supplementation of PVAT‐secreted CTRP4 through adipose tissue transplantation or circulatory CTRP4 by vein injection can alleviate carotid artery or aortic plaques in CTRP4^−/−^/ApoE^−/−^ mice; however, CTRP4^Mut^ failed to achieve such an effect, suggesting that CTRP4 has an inhibitory effect on atherosclerosis and this effect is mediated by binding and inhibiting of RAGE and TLR4.

## 4 DISCUSSION

**FIGURE 9:**
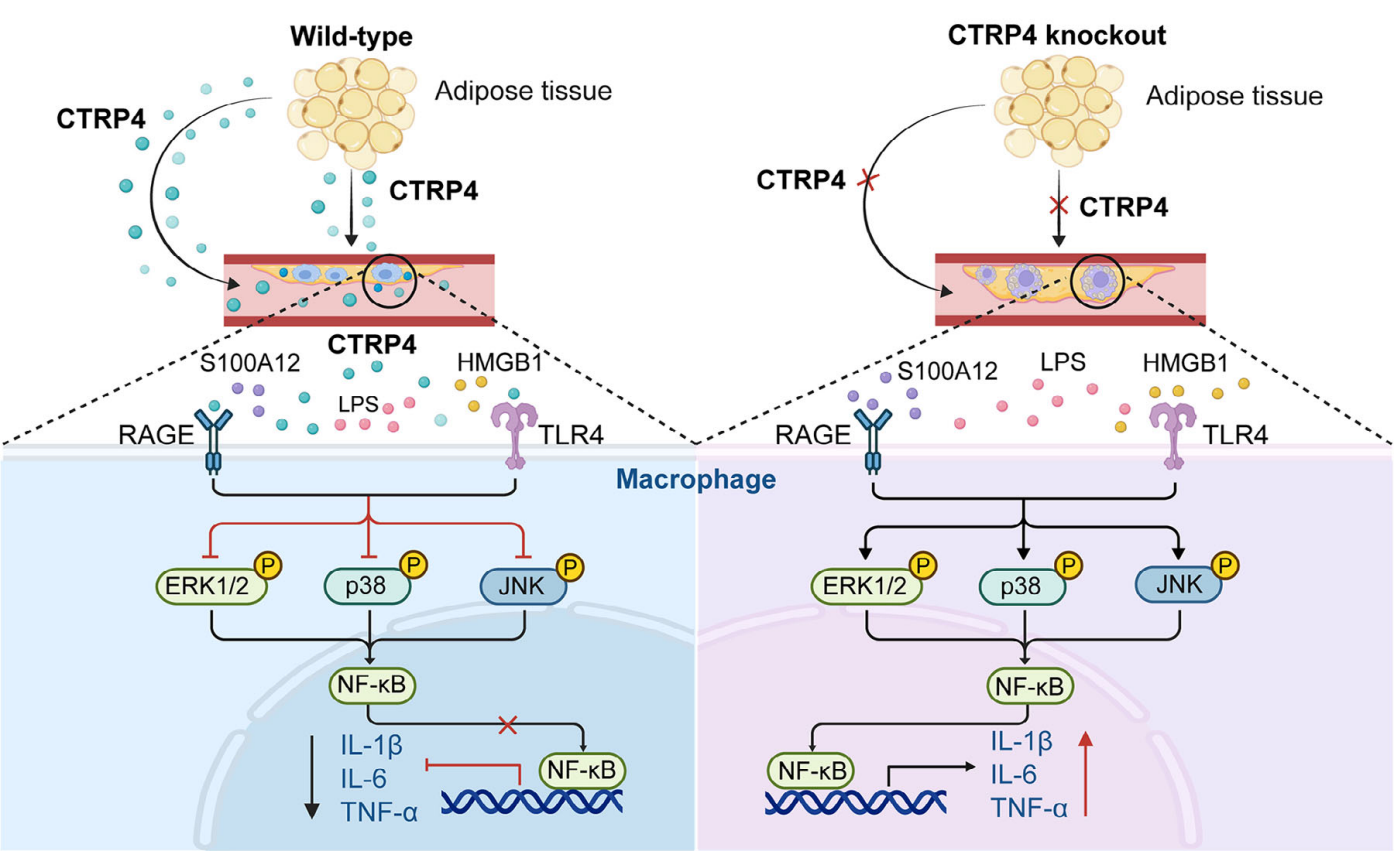
C1q/TNF‐related protein 4 (CTRP4) exerts anti‐inflammatory effects by engaging and inhibiting receptor for advanced glycation end‐products (RAGE) and toll‐like receptor 4 (TLR4) signalling pathways. Adipokine CTRP4, secreted by adipose tissue, inhibits the expression of inflammatory cytokines by binding and inhibition of RAGE and TLR4 in macrophages. This may lead to a reduction in inflammation and atherosclerosis.

The CTRPs family is involved in inflammation and atherosclerosis. The present study demonstrated that CTRP4 levels were lower in the serum and epicardial adipose tissue of patients with CAD compared with non‐CAD controls. CTRP4 knockout promoted atherosclerosis in ApoE^−/−^ mice. Supplementation of CTRP4 through adipose tissue transplantation from wild‐type mice or recombinant protein injection into the tail vein attenuated atherosclerosis in CTRP4^−/−^/ApoE^−/−^ mice. In vitro, CTRP4 inhibits the expression of LPS‐induced inflammatory cytokines in macrophages. The CTRP4 effects were mediated by engagement and inhibition of RAGE and TLR4 activation. These results suggest that CTRP4 is a protective adipokine that antagonises atherosclerosis (Figure 9).

CTRPs family members have different functions and activities. Some members have pro‐inflammatory effects, while others have anti‐inflammatory effects.^4^ Previous studies have shown that CTRPs family members differentially regulate the pathophysiology of atherosclerosis. CTRP3, CTRP9, CTRP12, CTRP13 and CTRP15 have been proven to play a protective role against atherosclerosis, whereas CTRP1, CTRP5 and CTRP7 exert pro‐atherosclerotic effects.^5^, ^6^, ^7^, ^8^ The present study showed that CTRP4 had anti‐inflammatory and anti‐atherogenic functions. Global knockout of CTRP4 promoted the development of atherosclerosis in ApoE^−/−^ mice. However, the carotid artery atherosclerosis in CTRP4^−/−^/ApoE^−/−^ mice was significantly reduced after transplantation of adipose tissue overexpressing CTRP4 to inhibit the ‘outside‐in’ pathway of atherogenesis. On the other hand, the supplementation of CTRP4 by tail vein injection attenuated atherosclerosis in CTRP4^−/−^/ApoE^−/−^ mice by antagonising ‘inside‐out’ pathway of atherogenesis. Our findings on the anti‐inflammatory role of CTRP4 align with those of Cao et al., who highlighted CTRP4's ability to modulate inflammation in macrophages and its protective effect against endotoxic shock.^35^

Based on the accumulated evidence, including ours, we propose that a dynamic equilibrium between pro‐inflammatory and anti‐inflammatory adipokines, including CTRPs, plays a crucial role in regulating vascular homeostasis. Beyond local adipose‒vascular crosstalk, systemic remodeling of circulating factors and the plasma proteome has been shown to reprogram monocyte/macrophage inflammatory states and thereby modulate atherogenesis.^36^ When severe atherosclerotic risk factors dominate, the levels of some protective anti‐inflammatory adipokines decrease, resulting in impaired vascular homeostasis, which eventually leads to the development of atherosclerosis.^1^, ^37^, ^38^ In this study, the levels of CTRP4 were reduced in the serum and epicardial adipose tissue of patients with CAD. CTRP4 levels in mouse adipose tissue decreased after a Western diet, accompanied by a consistent increase in inflammation in vivo. Pro‐atherogenic cues suppressed CTRP4 expression in adipocytes and macrophages. Collectively, these observations support the notion that CTRP4 downregulation is coupled to a pro‐inflammatory, metabolically stressed milieu, aligning with the emerging view that metabolic reprogramming is tightly coupled to inflammatory tone across metabolic disorders.^39^, ^40^

Our experiments demonstrated that after treating LPS‐induced macrophages with CTRP4, CTRP4 pulled down proteins, including RAGE and TLR4, as shown by immunoprecipitation‒mass spectrometry analysis. Co‐IP confirmed the binding of CTRP4 to RAGE and TLR4. SPR revealed that CTRP4 bound RAGE and TLR4 in a concentration‐dependent manner. Notably, previous studies have also reported that CTRP4 inhibits IL‐6‐induced signalling through its interaction with the IL‐6R.^31^ Consistently, even in the absence of RAGE, TLR4 or IL‐6R, CTRP4 was still able to attenuate inflammation signalling to varying degrees, suggesting that CTRP4 may regulate inflammation through several pathways. We further simulated the spatial conformation of CTRP4 bound to RAGE and TLR4 using molecular docking. In addition, CTRP4 mutation analysis and Co‐IP experiments revealed that CTRP4 interacted with RAGE and TLR4 via its D1 domain, where the CTRP4^Mut^ mutation sites were located. In vitro and in vivo experiments demonstrated that the anti‐inflammatory and anti‐atherogenic functions of RAGE‐ and TLR4‐binding incompetent CTRP4 mutants (CTRP4^D2^ and CTRP4^Mut^) were weakened compared with wild‐type CTRP4. These results indicate that CTRP4 is an endogenous inhibitor of inflammation and atherosclerosis, and its targets are RAGE and TLR4. This study also suggests that exogenous CTRP4 supplement or strategies to increase CTRP4 expression may be viable tools to antagonise the development of CAD.

To assess whether other CTRPs engage RAGE and TLR4 in a manner analogous to CTRP4, we examined CTRP1 and CTRP3 as representative pro‐ and anti‐atherogenic adipokines. In our study, both CTRP1 and CTRP3 bound RAGE, consistent with previous reports identifying CTRP1 as a novel RAGE ligand.^41^ However, only weak interactions between either CTRP1 or CTRP3 and TLR4 were detected in this study. Previous work has shown that CTRP1 modulates post‐myocardial infarction cardiac dysfunction via macrophage TLR4 signalling,^42^ and that CTRP3 functions as an endogenous LPS antagonist by sequestering LPS and preventing its engagement with the TLR4/MD‐2 complex, thereby attenuating TLR4‐dependent inflammatory responses.^43^ Taken together, these data suggest that CTRP1 and CTRP3 can act, at least in part, through TLR4 signalling while also directly engaging RAGE. Structural comparison further revealed that CTRP4 uniquely contains two globular C1q head domains, whereas other CTRPs harbour only one, which may contribute to the distinct receptor‐binding profile of CTRP4. Notably, our docking analysis mapped the putative RAGE‐interacting residues of CTRP1 and CTRP3 to their C1q head domains, supporting the notion that the C1q head domain is a critical structural module for receptor engagement across the CTRP family.

We acknowledge the limitations of this study. Regarding the investigations of CAD, this study was cross‐sectional, allowing us to detect only an association between CTRP4 and CAD. The effects of CTRP4 will be further investigated in future prospective studies. Second, it would be better to decipher whether CTRP4 family members maintain a balance between pro‐ and anti‐inflammation in physiological status, and how this balance is impaired in disease conditions. And does this inflammatory balance of CTRP family is related to RAGE or TLR4. Finally, the cell type‐specific contribution of CTRP4 to atherogenesis also warrants further investigation.

In conclusion, this study has demonstrated that the anti‐inflammatory and anti‐atherogenic effects of CTRP4 are mediated by engagement and inhibition of RAGE and TLR4.

## AUTHOR CONTRIBUTIONS

Xinyi Shu, Feifei Li, Fenghua Ding, Ying Shen, Qiujing Chen, Weifeng Shen, Ruiyan Zhang, Yang Dai, Xinrui Wu and Lin Lu conceived and designed the study. Xinyi Shu, Feifei Li, Jiawei Chen and Xinrui Wu conducted the experiments. Shuai Chen, Jinwei Quan, Jingmeng Liu, Yipaerguli Maimati, Leyuan Tao and Abulikemu Amuti analysed the data. Xinyi Shu and Feifei Li drafted the manuscript. Xiaoqun Wang and Lin Lu revised the article before the final approval.

## CONFLICT OF INTEREST STATEMENT

The authors declare they have no conflicts of interest.

## ETHICS STATEMENT

All human samples were obtained and processed in accordance with the principles of the Declaration of Helsinki (2013) and the protocols approved by the Institutional Review Committee of Shanghai Jiao Tong University (approval no. RJ‐CAMPUS‐2022177). Written informed consent was obtained from all participants prior to the inclusion in this study. All animal experiments were approved by the Animal Care Committee of Shanghai Jiao Tong University (approval no. RJ2023027).

## Supporting information



Supporting Information

Supporting Information

Supporting Information

## Data Availability

The data that support the findings of this study are available from the corresponding author upon reasonable request.
