# Supplementary material for: Decreased adipokine CTRP4 in CAD patients: CTRP4 attenuates atherosclerosis via inhibition of RAGE and TLR4
Source: Clin Transl Med. 2026 Feb 18;16(2):e70624. doi: 10.1002/ctm2.70624 (PMC12914348; doi:10.1002/ctm2.70624)
Supplement: Supplementary file 1 — Supporting Information [file CTM2-16-e70624-s002.docx]

**Major Resources Table**

**Animals**

| **Species** | **Genotype** | **Vendor or Source** | **Background Strain** | **Strain NO.** | **Sex** | **Persistent ID / URL** |
| --- | --- | --- | --- | --- | --- | --- |
| Mouse | Wild-type | GemPharmatech | C57BL/6JGpt | N000013 | Male | https://cn.gempharmatech.com/shop/productDetails/12402.html |
| Mouse | Apoe KO | Cyagen | C57BL/6JCya | C001507 | Male | https://www.cyagen.com/cn/zh-cn/business/drug-filter-evaluate-mouse/metabolism-medicinal/B6J-Apoe-KO.html |

**Antibodies**

| **Target antigen** | **Vendor or Source** | **Catalog #** | **Working concentration** | **Persistent ID / URL** |
| --- | --- | --- | --- | --- |
| Rabbit anti- CTRP4 | Sigma-Aldrich | PRS3567 | 1:100 (IHC/IF)  1:1000 (WB) | https://www.sigmaaldrich.cn/CN/zh/product/sigma/prs3567 |
| Rabbit anti- Phospho-p44/42 MAPK (Erk1/2) (Thr202/Tyr204) | Cell Signaling Technology | 9101L | 1:1000 | https://www.cellsignal.cn/products/primary-antibodies/phospho-p44-42-mapk-erk1-2-thr202-tyr204-antibody/9101 |
| Rabbit anti- p44/42 MAPK (Erk1/2) | Cell Signaling Technology | 9102L | 1:1000 | https://www.cellsignal.cn/products/primary-antibodies/p44-42-mapk-erk1-2-antibody/9102 |
| Rabbit anti- Phospho-p38 MAPK (Thr180/Tyr182) | Cell Signaling Technology | 9211S | 1:1000 | https://www.cellsignal.cn/products/primary-antibodies/phospho-p38-mapk-thr180-tyr182-antibody/9211 |
| Rabbit anti- p38 MAPK | Cell Signaling Technology | 9212S | 1:1000 | https://www.cellsignal.cn/products/primary-antibodies/p38-mapk-antibody/9212 |
| Rabbit anti- Phospho-SAPK/JNK (Thr183/Tyr185) | Cell Signaling Technology | 9251L | 1:1000 | https://www.cellsignal.cn/products/primary-antibodies/phospho-sapk-jnk-thr183-tyr185-antibody/9251 |
| Rabbit anti- SAPK/JNK Antibody | Cell Signaling Technology | 9252L | 1:1000 | https://www.cellsignal.cn/products/primary-antibodies/sapk-jnk-antibody/9252 |
| Rabbit anti-Phospho-NF-κB p65 (Ser536) | Cell Signaling Technology | 3033L | 1:1000 (WB) | https://www.cellsignal.cn/products/primary-antibodies/phospho-nf-kb-p65-ser536-93h1-rabbit-mab/3033 |
| Rabbit anti- NF-κB p65 | Abmart | T55034 | 1:3000 (WB) | https://www.ab-mart.com.cn/page.aspx?node=%2077%20&id=%201338 |
| Mouse anti-RAGE | Santa Cruz | Sc-365154 | 1:500 (WB) | https://www.scbt.com/zh/p/rage-antibody-a-9 |
| Mouse anti-TLR4 | Santa Cruz | sc-293072 | 1:500 (WB) | https://www.scbt.com/zh/p/tlr4-antibody-25 |
| Mouse anti-FLAG-Tag | Sigma-Aldrich | F1804 | 1:400 (IP)  1:1000 (WB) | https://www.sigmaaldrich.cn/CN/zh/product/sigma/f1804 |
| Mouse anti-His-tag | Sigma-Aldrich | SAB2702218 | 1:500 (IP)  1:1000 (WB) | https://www.sigmaaldrich.cn/CN/zh/product/sigma/sab2702218 |
| Rabbit anti-IL-1 beta | Abcam | ab9722 | 1:1000 | https://www.abcam.cn/products/primary-antibodies/il-1-beta-antibody-ab9722.html |
| Rabbit anti-TNF alpha | Abcam | Ab183218 | 1:500 | https://www.abcam.cn/products/primary-antibodies/tnf-alpha-antibody-ab66579.html |
| Mouse anti-  IL-6 | Santa Cruz | sc-57315 | 1:500 | https://www.scbt.com/zh/p/il-6-antibody-10e5 |
| Rabbit anti-CTRP1 | Proteintech | 30289-1-AP | 1:1000 | https://www.ptgcn.com/products/CTRP1-Antibody-30289-1-AP.htm |
| Rabbit anti-CTRP3 | Signalway Antibody | 42952 | 1:1000 | https://www.sabbiotech.cn/products/42952 |
| Rabbit anti-IL-6R alpha | Proteintech | 23457-1-AP | 1:1000 | https://www.ptgcn.com/products/IL6R-Antibody-23457-1-AP.htm |
| Rabbit anti-  β-Tubulin | Proteintech | 10068-1-AP | 1:1000 | https://www.ptgcn.com/products/TUBB3-Antibody-10068-1-AP.htm |
| Mouse anti-  GAPDH | Proteintech | 60004-1-Ig | 1:5000 | https://www.ptgcn.com/products/GAPDH-Antibody-60004-1-Ig.htm |
| Rat anti-MOMA-2 | Bio-Rad | MCA519G | 1:50 (IF) | https://www.bio-rad-antibodies.com/monoclonal/mouse-macrophages-monocytes-antibody-moma-2-mca519.html?f=purified |
| Goat anti-rabbit IgG HRP | Cell Signaling Technology | 7074 | 1:5000 (WB) | https://www.cellsignal.cn/products/secondary-antibodies/anti-rabbit-igg-hrp-linked-antibody/7074 |
| Horse anti-mouse IgG HRP | Cell Signaling Technology | 7076 | 1:5000 (WB) | https://www.cellsignal.cn/products/secondary-antibodies/anti-rabbit-igg-hrp-linked-antibody/7074 |
| Donkey anti-rabbit IgG (Alexa Fluor™ 555) | Invitrogen | A-31572 | 1:1000 (IF) | https://www.thermofisher.cn/cn/zh/antibody/product/Donkey-anti-Rabbit-IgG-H-L-Highly-Cross-Adsorbed-Secondary-Antibody-Polyclonal/A-31572 |
| Goat anti-mouse IgG (Alexa Fluor™ 488) | Invitrogen | A-11001 | 1:1000 (IF) | https://www.thermofisher.cn/cn/zh/antibody/product/Goat-anti-Mouse-IgG-H-L-Cross-Adsorbed-Secondary-Antibody-Polyclonal/A-11001 |
| Donkey anti-rat IgG (Alexa Fluor™ 488) | Invitrogen | A-21208 | 1:1000 (IF) | https://www.thermofisher.cn/cn/zh/antibody/product/Donkey-anti-Rat-IgG-H-L-Highly-Cross-Adsorbed-Secondary-Antibody-Polyclonal/A-21208 |

**Cultured Cells**

| **Name** | **Vendor or Source** | **Sex** | **Catalog**  **#** | **Persistent ID / URL** |
| --- | --- | --- | --- | --- |
| HEK 293 cell line | China Center for Type  Culture Collection (Wuhan  University) | unknown | GDC0067 | http://cctcc.whu.edu.cn/portal/no_center/cell_detail?id=53 |
| Mouse bone marrow-derived macrophages (BMDMs) | This paper | Male | NA | NA |
| THP-1 cells | Ethephon Biotechnology (Shanghai) Co., Ltd. | Male | YCL-0351 | https://yzfbio.com/Product/details.html?id=498 |
| 3T3-L1 cells | Ethephon Biotechnology (Shanghai) Co., Ltd. | NA | YCL-0009 | https://yzfbio.com/Product/details.html?id=192 |

**Chemicals and assay kits**

| **Description** | **Source / Repository** | **Catalog**  **#** | **Persistent ID / URL** |
| --- | --- | --- | --- |
| RPMI Medium 1640 | Bioagrio | LR1634 | https://www.yulibio.cn/?products_13/184.html |
| Minimum Essential Medium (MEM) | Corning | 10-010-CV | https://ecatalog.corning.com/life-sciences/b2b/US/en/Media,-Sera,-and-Reagents/Classical-Media/Minimum-Essential-Medium/Corning%C2%AE-MEM-(Minimum-Essential-Medium)/p/10-010-CV |
| Fetal bovine serum | Gibco | 10099141C | https://www.thermofisher.cn/order/catalog/product/10099141C?SID=srch-hj-10099141C |
| MEM Non-Essential Amino Acids | Gibco | 11140050 | https://www.thermofisher.cn/order/catalog/product/11140050?SID=srch-srp-11140050 |
| Penicillin Streptomycin | Gibco | 15140-122 | https://www.thermofisher.cn/order/catalog/product/15140122?SID=srch-srp-15140122 |
| 0.25% Tripsin-EDTA | Gibco | 25200-056 | https://www.thermofisher.cn/order/catalog/product/25200056?SID=srch-hj-25200-056 |
| Phosphate buffered saline | Servicebio | G4202 | https://www.servicebio.cn/goodsdetail?id=4118 |
| Lipopolysaccharide | Sigma-Aldrich | L5293 | https://www.sigmaaldrich.cn/CN/zh/product/sigma/l5293 |
| Recombinant Mouse M-CSF | BioLegend | 576406 | https://www.biolegend.com/ja-jp/products/recombinant-mouse-m-csf-carrier-free-7780?GroupID=GROUP577 |
| Control siRNA | Santa Cruz | Sc-37007 | https://www.scbt.com/zh/p/control-sirna-a |
| RAGE siRNA | Santa Cruz | sc-36375 | https://www.scbt.com/zh/p/rage-sirna-m-shrna-and-lentiviral-particle-gene-silencers |
| TLR4 siRNA | Santa Cruz | sc-40261 | https://www.scbt.com/zh/p/tlr4-sirna-m-shrna-and-lentiviral-particle-gene-silencers |
| IL-6Rα siRNA | Santa Cruz | sc-40065 | https://www.scbt.com/zh/p/il-6ralpha-sirna-m-shrna-and-lentiviral-particle-gene-silencers |
| HMGB1 | GenScript | Z02803 | https://www.genscript.com/protein/Z02803-HMGB1_His_Human.html?page_no=1&position_no=1&sensors=googlesearch |
| S100A12 | Novoprotein | C743 | https://www.novoprotein.com.cn/product-detail?productNumber=C743 |
| Phorbol 12-myristate 13-acetate (PMA) | MCE | HY-18739 | https://www.medchemexpress.cn/Phorbol-12-myristate-13-acetate.html |
| Rosiglitazone | MCE | HY-17386 | https://www.medchemexpress.cn/Rosiglitazone.html |
| Dexamethasone phosphate disodium | MCE | HY-B1829A | https://www.medchemexpress.cn/dexamethasone-phosphate-disodium.html |
| Insulin | MCE | HY-P0035 | https://www.medchemexpress.cn/Insulin_human_.html |
| IBMX | MCE | HY-12318 | https://www.medchemexpress.cn/IBMX.html |
| Pierce™ IP lysis buffer | Thermo Scientific | 87788 | https://www.thermofisher.cn/order/catalog/product/87788?SID=srch-srp-87788 |
| Protein A/G Magnetic Beads for IP | Selleck | B23202 | https://www.selleck.cn/bioreagents/protein-a-g-magnetic-beads-for-ip.html |
| Protease and Phosphatase Inhibitor Cocktail | NCM Biotech | P002 | http://www.ncmbio.com/product/show-138.html |
| Oil red O | Servicebio | G1015 | https://www.servicebio.cn/goodsdetail?id=1765 |
| DAPI | Servicebio | G1012 | https://www.servicebio.cn/goodsdetail?id=1762 |
| BCA Protein Assay Kit | CWBIO | CW0014S | https://www.cwbio.com/product/detail/id/10069 |
| Light chemiluminescence kit | EpiZyme | SQ201 | http://www.epizyme.cn//search.aspx?keyword=SQ201 |
| TRIzol reagent | Invitrogen | 15596026CN | https://www.thermofisher.cn/order/catalog/product/15596026CN?SID=srch-srp-15596026CN |
| Lipofectamine 3000 | Invitrogen | L3000015 | https://www.thermofisher.cn/order/catalog/product/L3000015?SID=srch-hj-L3000015 |
| Lipofectamine RNAiMAX | Invitrogen | 13778150 | https://www.thermofisher.cn/order/catalog/product/cn/en/13778150 |
