# Supplementary material for: Decreased adipokine CTRP4 in CAD patients: CTRP4 attenuates atherosclerosis via inhibition of RAGE and TLR4
Source: Clin Transl Med. 2026 Feb 18;16(2):e70624. doi: 10.1002/ctm2.70624 (PMC12914348; doi:10.1002/ctm2.70624)
Supplement: Supplementary file 2 — Supporting Information [file CTM2-16-e70624-s003.docx]

**Supplemental information**

**Decreased Adipokine CTRP4 in CAD Patients: CTRP4 Attenuates Atherosclerosis via Inhibition of RAGE and TLR4**

**Short title**: CTRP4 inhibits atherosclerosis

**Supplementary Table 1. Baseline characteristics and parameters of control subjects and patients with CAD.**

|  | **Health subjects** | | | | **CAD** | | | | | | | **P value** |
| --- | --- | --- | --- | --- | --- | --- | --- | --- | --- | --- | --- | --- |
| **Number of diseased vessels** | **0** | | **1** | | | **2** | | **≥3** | | |  | |
| **n** | **315** | | **155** | | | **101** | | **67** | | |  | |
| Male, n (%) | | 179 (56.8) | | 105 (67.7) | | | 75 (74.2) | | 55 (82.1) | | | <0.001 |
| Age, years | | 61.40 ± 11.72 | | 63.92 ± 11.55 | | | 65.53 ± 11.15 | | 64.78 ± 11.52 | | | 0.004 |
| Body mass index, kg/m^2^ | | 24.93 ± 4.38 | | 26.89 ± 5.90 | | | 25.53 ± 4.78 | | 26.73 ± 5.50 | | | <0.001 |
| Current smoking, n (%) | | 90 (28.6) | | 50 (32.2) | | | 39 (38.6) | | 33 (49.3) | | | 0.008 |
| Diabetes mellitus, n (%) | | 156 (49.5) | | 82 (52.9) | | | 52 (51.4) | | 30 (44.7) | | | 0.714 |
| Hypertension, n (%) | | 150 (47.6) | | 114 (73.5) | | | 75 (74.2) | | 51 (76.1) | | | <0.001 |
| HbA1c, % | | 6.43 ± 1.22 | | 6.59 ± 1.19 | | | 6.71 ± 1.45 | | 7.04 ± 2.84 | | | 0.016 |
| eGFR, mL/min/1.73 m^2^ | | 86.97 ± 61.96 | | 82.07 ± 29.72 | | | 78.63 ± 19.68 | | 80.09 ± 20.39 | | | 0.354 |
| Triglyceride, mmol/L | | 1.56 ± 0.79 | | 1.68 ± 1.07 | | | 1.66 ± 1.34 | | 1.71 ± 1.47 | | | 0.509 |
| Total cholesterol, mmol/L | | 4.04 ± 1.09 | | 4.09 ± 1.32 | | | 3.99 ± 1.28 | | 4.15 ± 1.66 | | | 0.846 |
| LDL-C, mmol/L | | 2.32 ± 0.91 | | 2.39 ± 1.13 | | | 2.21 ± 0.97 | | 2.48 ± 1.43 | | | 0.329 |
| HDL-C, mmol/L | | 1.21 ± 0.45 | | 1.10 ± 0.27 | | | 1.08 ± 0.28 | | | 1.02 ± 0.22 | | <0.001 |
| hsCRP, mg/L | | 1.41 ± 2.96 | | 4.60 ± 18.73 | | | 3.03 ± 8.70 | | | 4.44 ± 11.16 | | 0.011 |
| CTRP4, ng/mL | | 17.30 ± 15.03 | | 12.25 ± 8.41 | | | 10.83 ± 8.57 | | | 8.60 ± 7.69 | | <0.001 |
| Gensini score | | / | | 6.40 ± 3.87 | | | 16.94 ± 10.90 | | | 51.13 ± 27.78 | | <0.001* |
| SYNTAX score | | / | | 6.06 ± 4.11 | | | 15.91 ± 7.14 | | | 28.71 ± 12.60 | | <0.001* |

Values are given as mean ± SD, median (25th–75th percentile), or number (percentage).

CAD, coronary artery disease; HbA1c, glycated hemoglobin; eGFR, estimated glomerular filtration rate; LDL-C, low‐density lipoprotein cholesterol; HDL-C, high‐density lipoprotein cholesterol; hsCRP, high‐sensitivity C‐reactive protein; and CTRP4, C1q/TNF-related protein 4.

*Only between CAD patients

**Supplementary Table 2. Multivariate Logistic Regression Analyses for CAD in the participants.**

| **Models** | **CTRP4** | **OR (95% CI)** | **P value** |
| --- | --- | --- | --- |
| Model 1 | CTRP4 (per SD) | 0.53 (0.43 – 0.66) | <0.001 |
|  | ≥12.22 | Reference | / |
|  | <12.22 | 2.32 (1.69 – 3.19) | <0.001 |
| Model 2 | CTRP4 (per SD) | 0.52 (0.42 – 0.65) | <0.001 |
|  | ≥12.22 | Reference | / |
|  | <12.22 | 2.32 (1.68 – 3.23) | <0.001 |
| Model 3 | CTRP4 (per SD) | 0.55 (0.44 – 0.69) | <0.001 |
|  | ≥12.22 | Reference | / |
|  | <12.22 | 2.20 (1.56 – 3.11) | <0.001 |

Model 1, unadjusted; Model 2, adjusted for age and sex; Model 3, additional adjusted for age, sex, history of hypertension, history of diabetes mellitus, body mass index, smoking, and estimated glomerular filtration rate (eGFR). CTRP4, C1q/TNF-related protein 4; and OR, odds ratio.**Supplementary Table 3. Baseline characteristics of CAD patients providing epicardial adipose tissue for RNA sequencing, Western blot and immunostaining.**

|  | **Control**  **(n=9)** | **CAD**  **(n=9)** | **P value** |
| --- | --- | --- | --- |
| Male, n (%) | 7 (77.8) | 8 (88.9) | >0.999 |
| Age, years | 66.33 ± 9.10 | 68.67 ± 8.69 | 0.586 |
| Body mass index, kg/m^2^ | 23.72 ± 2.70 | 24.92 ± 2.53 | 0.347 |
| Current smoking, n (%) | 5 (55.6) | 6 (66.7) | 0.620 |
| Hypertension, n (%) | 7 (77.8) | 8 (88.9) | >0.999 |
| HbA1c, % | 6.94 ± 1.35 | 7.22 ± 1.56 | 0.691 |
| Triglyceride, mmol/L | 1.32 ± 0.28 | 1.56 ± 0.79 | 0.913 |
| Total cholesterol, mmol/L | 3.89 ± 0.93 | 4.58 ± 0.73 | 0.100 |
| LDL-C, mmol/L | 2.23 ± 0.78 | 2.77 ± 0.79 | 0.160 |
| HDL-C, mmol/L | 1.09 ± 0.17 | 1.05 ± 0.22 | 0.681 |
| hsCRP, mg/L | 2.51 (0.94 – 5.81) | 2.80 (0.66 – 8.49) | 0.749 |
| Gensini score | 0 | 49.22 ± 23.70 | <0.001 |

Values are given as mean ± SD, median (25th–75th percentile), or number (percentage). CAD, coronary artery disease; HbA1c, glycated hemoglobin; eGFR, estimated glomerular filtration rate; LDL-C, low‐density lipoprotein cholesterol; HDL-C, high‐density lipoprotein cholesterol; and hsCRP, high‐sensitivity C‐reactive protein.
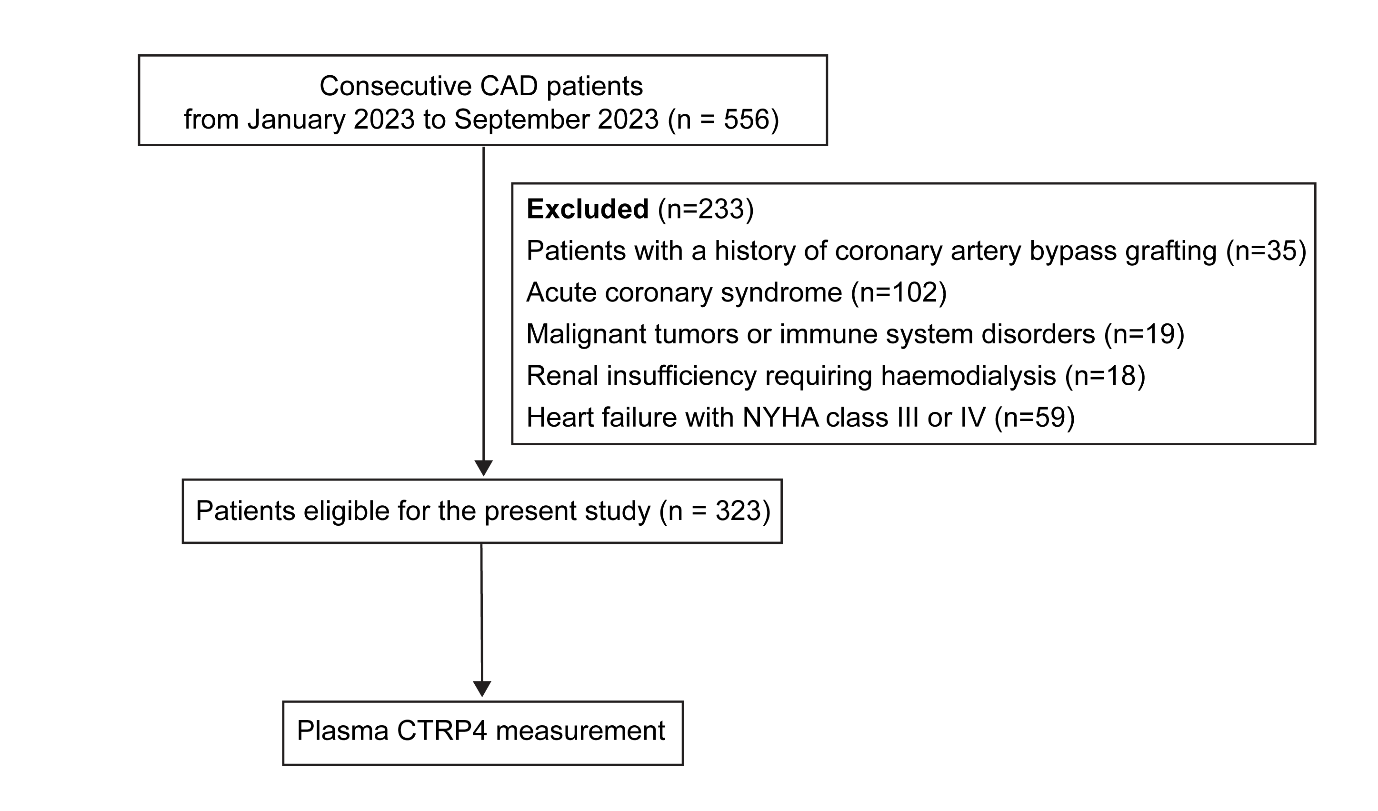


**Supplementary Figure 1**. **Flowchart of patient enrollment**.
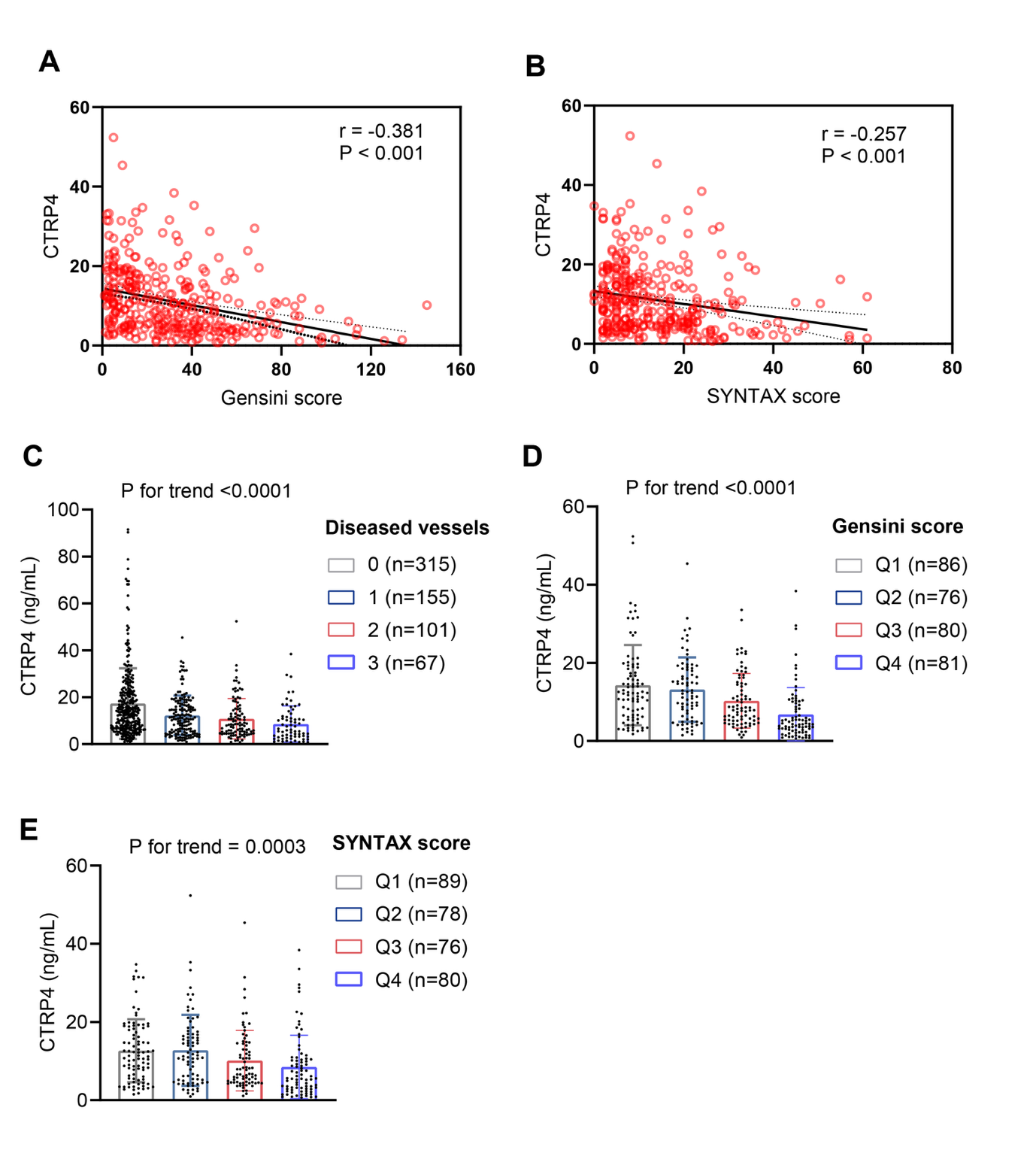


**Supplementary Figure 2. Association of serum CTRP4 levels with the severity of CAD in patients.**

A and B, Correlation analysis between CTRP4 and Gensini or SYNTAX scores, respectively, using the Spearman correlation coefficient.

C, Serum CTRP4 concentration was associated with the number of diseased coronary arteries.

D and E, The association between serum CTRP4 levels and Gensini and SYNTAX scores, stratified by quartiles, was analyzed.

Data are presented as mean ± SD. Data in **C** to **E** were analyzed using one-way ANOVA tests.
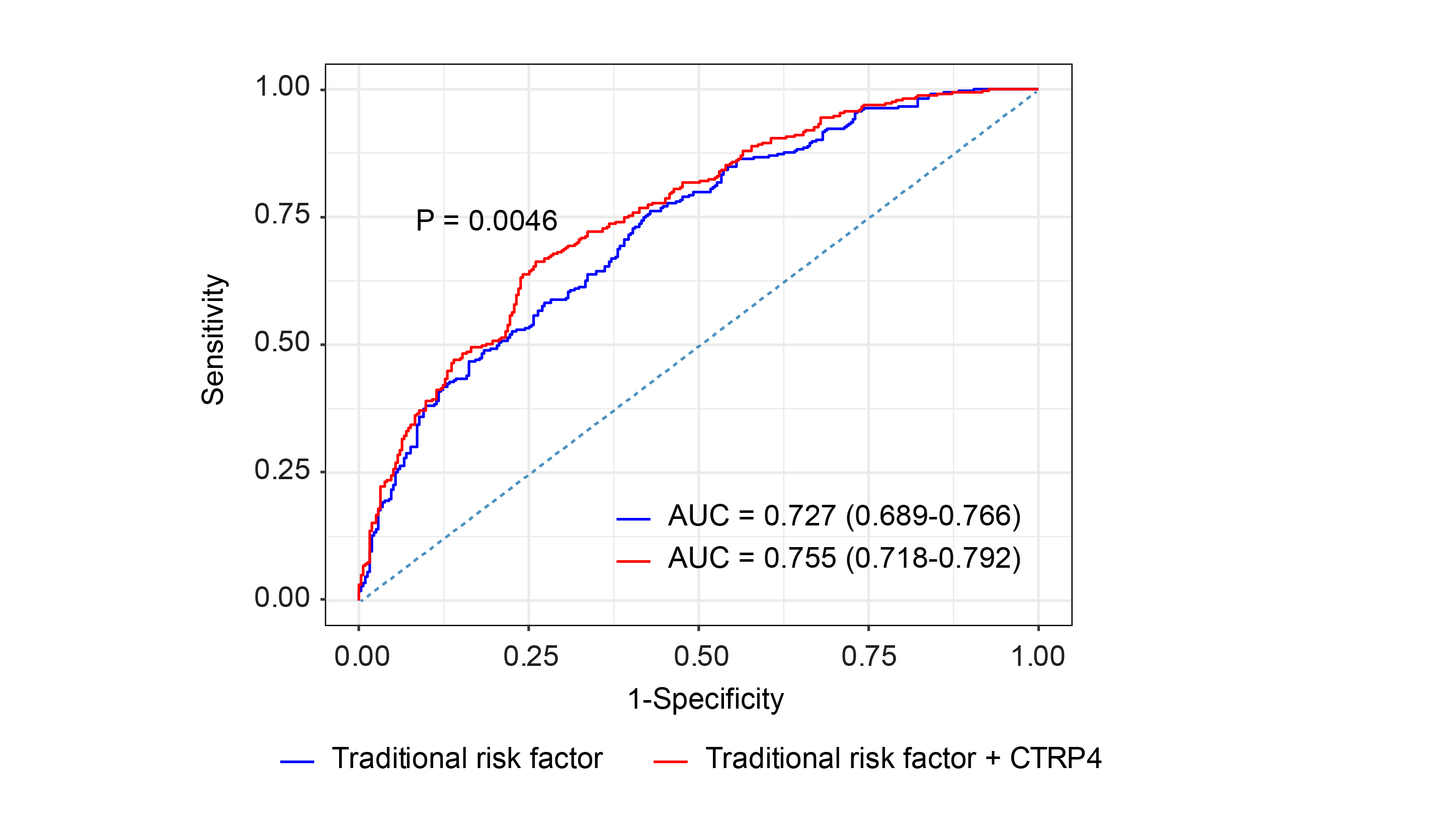
 **Supplementary Figure 3. ROC curve analysis for predicting CAD.**

Predicted probabilities derived from regression models for CAD detection. Traditional risk factors include male sex, age, body mass index, hypertension, glycated hemoglobin, cigarette smoking, LDL-C, HDL-C, eGFR, and hsCRP. P value was calculated using DeLong’s test. LDL-C, low‐density lipoprotein cholesterol; HDL-C, high‐density lipoprotein cholesterol; eGFR, estimated glomerular filtration rate; hsCRP, high‐sensitivity C‐reactive protein; and CTRP4, C1q/TNF-related protein 4.

**
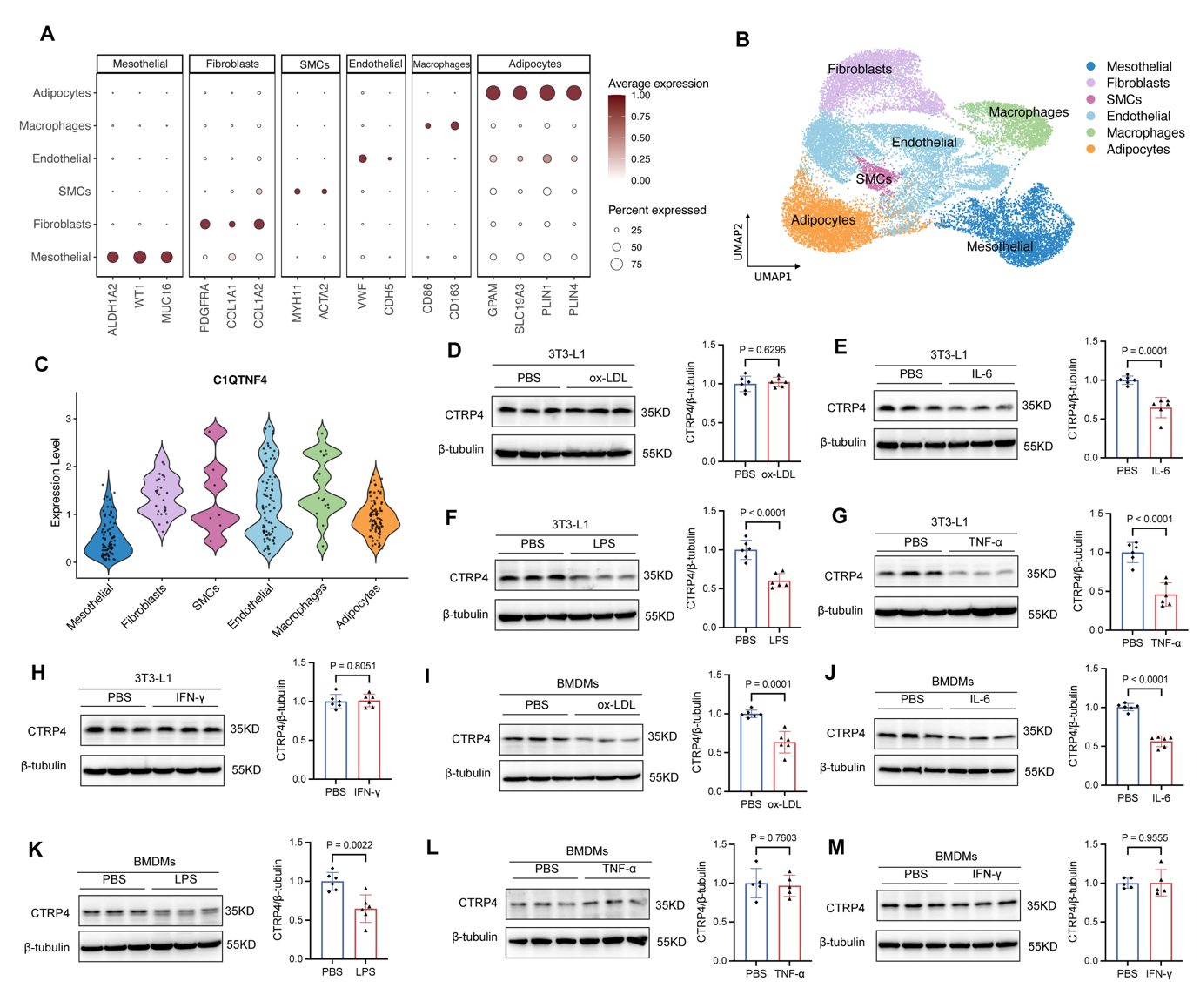
Supplementary Figure 4. CTRP4 expression is reduced by pro-atherogenic stimuli in adipocytes and macrophages.**

A–C, Single-nucleus RNA sequencing (snRNA-seq) analysis of the cellular distribution of C1QTNF4 expression in human perivascular adipose tissue (PVAT) (GSE166355, n=3). Dot plot showing canonical marker genes used for cell-type annotation (A). UMAP visualization of annotated cell clusters (B). Feature plot showing CTRP4 expression across the indicated cell populations (C).

D–M, Differentiated 3T3-L1 adipocytes (D–H) and bone marrow–derived macrophages (BMDMs) from wild-type mice (I–M) were treated with oxLDL (100 µg/mL), IL-6 ( 50 ng/mL), LPS (50 ng/mL), TNF-α (50 ng/mL), or IFN-γ(50 ng/mL) for 24 hours, and CTRP4 protein levels were assessed by Western blot (n=6).

Data are presented as mean ± SD. Data in **D** to **M** were analyzed by using unpaired Student’s t-test. CTRP4, C1q/TNF-related protein 4; IFN-γ, interferon-γ; IL-1β, interleukin-1 beta; IL-6, interleukin-6; LPS, lipopolysaccharide; oxLDL, oxidized low-density lipoprotein; and TNF-α, tumor necrosis factor-alpha.


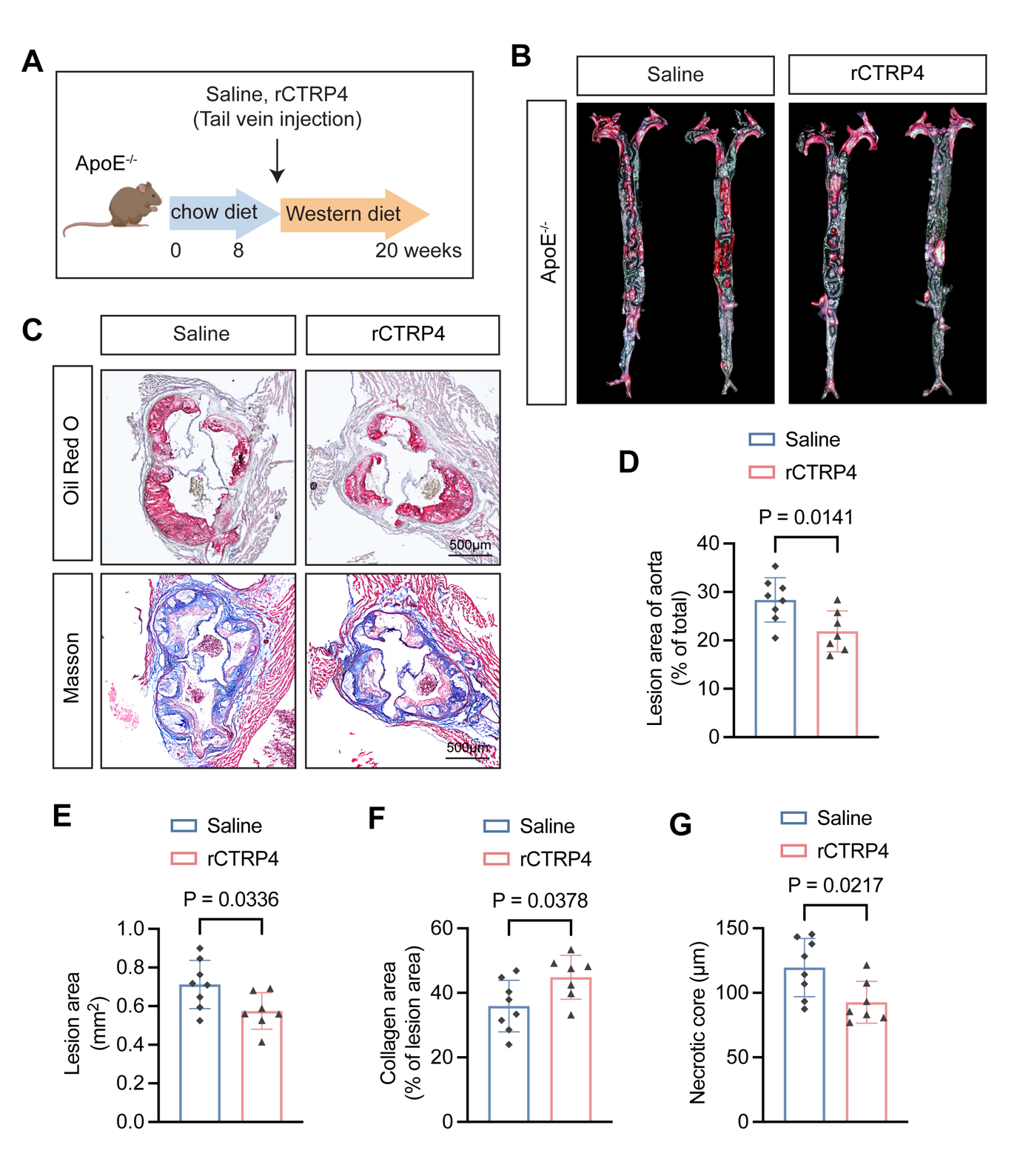


**Supplementary Figure 5. Injection of recombinant CTRP4 attenuated atherogenesis in ApoE^⁻/⁻^ mice.**

A, Experimental procedure: 8-week-old male ApoE^⁻/⁻^ mice were injected through the tail vein with recombinant protein CTRP4 (10 μg/mouse, once every other day) or saline, and the atherosclerosis models were constructed after 12 weeks of Western diet (n=7–8 per group).

B, *En face* analysis of aortic plaque area in ApoE^⁻/⁻^ mice injected with saline, recombinant CTRP4.

C, Atherosclerotic plaque area, collagen content, necrotic core diameter (Scale bar, 500 μm) were evaluated by Oil Red O, and Masson’s staining of aortic roots in ApoE^⁻/⁻^ mice injected with saline or recombinant CTRP4 protein (n=7–8 per group).

D, Quantification of the data in B (n=7–8 per group).

E-G, Quantification of the data in C (n=7–8 per group).

Data are presented as mean ± SD. Data in **D** to **G** were analyzed by using unpaired Student’s t-test. ApoE, apolipoprotein E; and CTRP4, C1q/TNF-related protein 4.

**
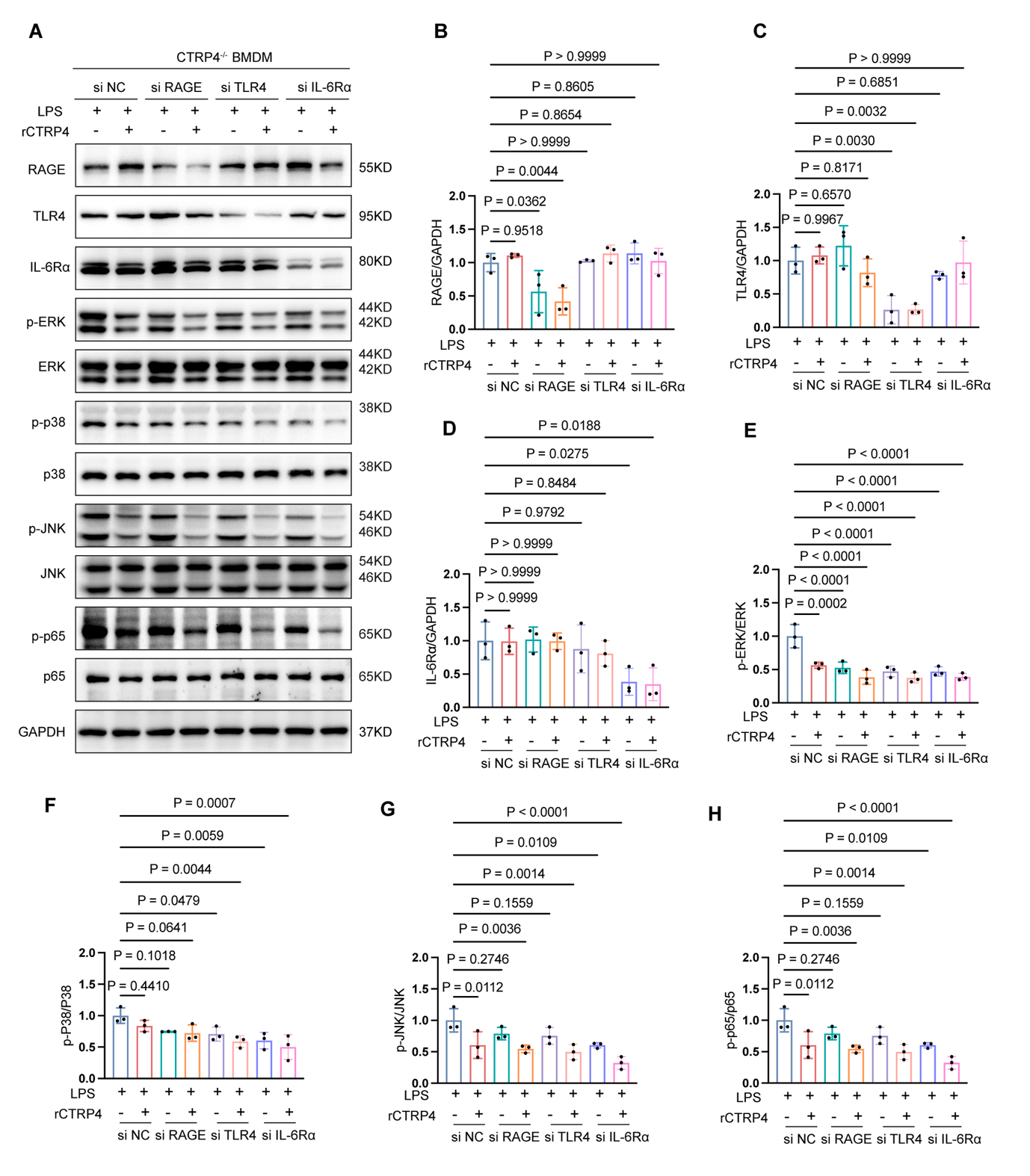
Supplementary Figure 6. CTRP4 recombinant protein inhibits LPS-induced inflammatory reaction in CTRP4^−/−^ macrophages with RAGE, TLR4 or IL-6R knockdown.**

A, BMDMs from CTRP4^−/−^ mice were transfected with siRNA-RAGE (si RAGE), siRNA-TLR4 (si TLR4), and siRNA-IL-6Rα (si IL-6Rα), respectively. These cells were later treated with LPS (50 ng/mL) for 40 min in the presence or absence of CTRP4 recombinant protein (1 μg/mL). The protein levels of RAGE, TLR4 or IL-6Rα, and phosphorylation levels of ERK, p38, JNK, and p65 were detected using Western blot.

B–H, Quantification of the data in A (n=3).

Data are presented as mean ± SD. Data in **B** to **H** were analyzed by using one-way ANOVA followed by Bonferroni post hoc tests. BMDMs, bone marrow-derived macrophages; CTRP4, C1q/TNF-related protein 4; IL-6Rα, IL-6 alpha-receptor; LPS, lipopolysaccharide; NC, negative control; p-ERK, phosphorylated extracellular signal-regulated kinase; p-JNK, phosphorylated c-Jun N-terminal kinase; p-p38, phosphorylated p38 mitogen-activated protein kinase; p-p65, phosphorylated p65 subunit of NF-κB; RAGE, receptor for advanced glycation end products; and TLR4, Toll-like receptor 4.**
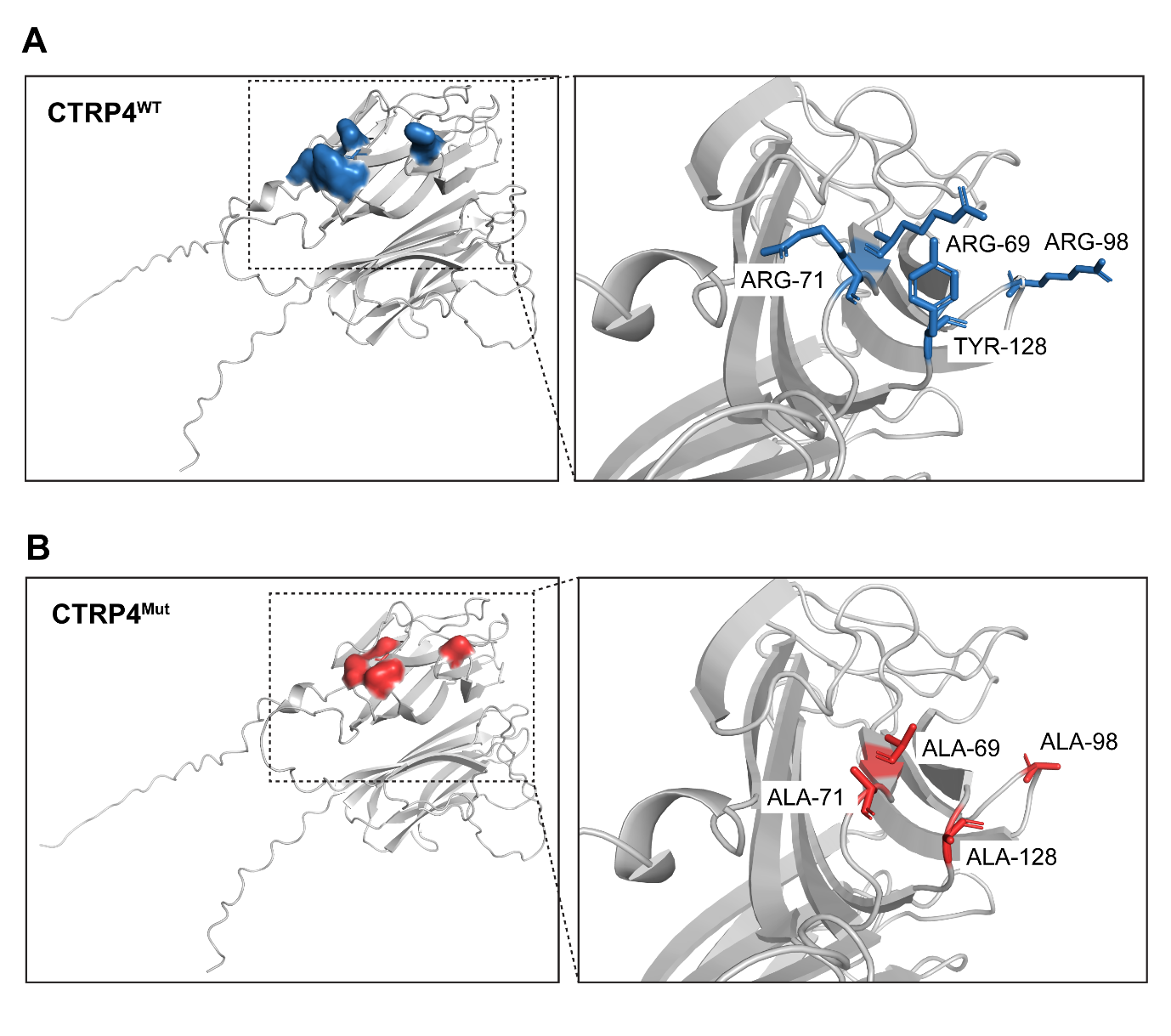
 Supplementary Figure 7. The construction of mutant CTRP4 protein.**

A, Structure of the wild-type CTRP4 (CTRP4^WT^).

B, Structure of the CTRP4 mutant (CTRP4^Mut^), in which ARG69, ARG71, ARG98, and TYR128 were mutated to ALA.

ALA, alanine; ARG, arginine; and TYR, tyrosine.

**
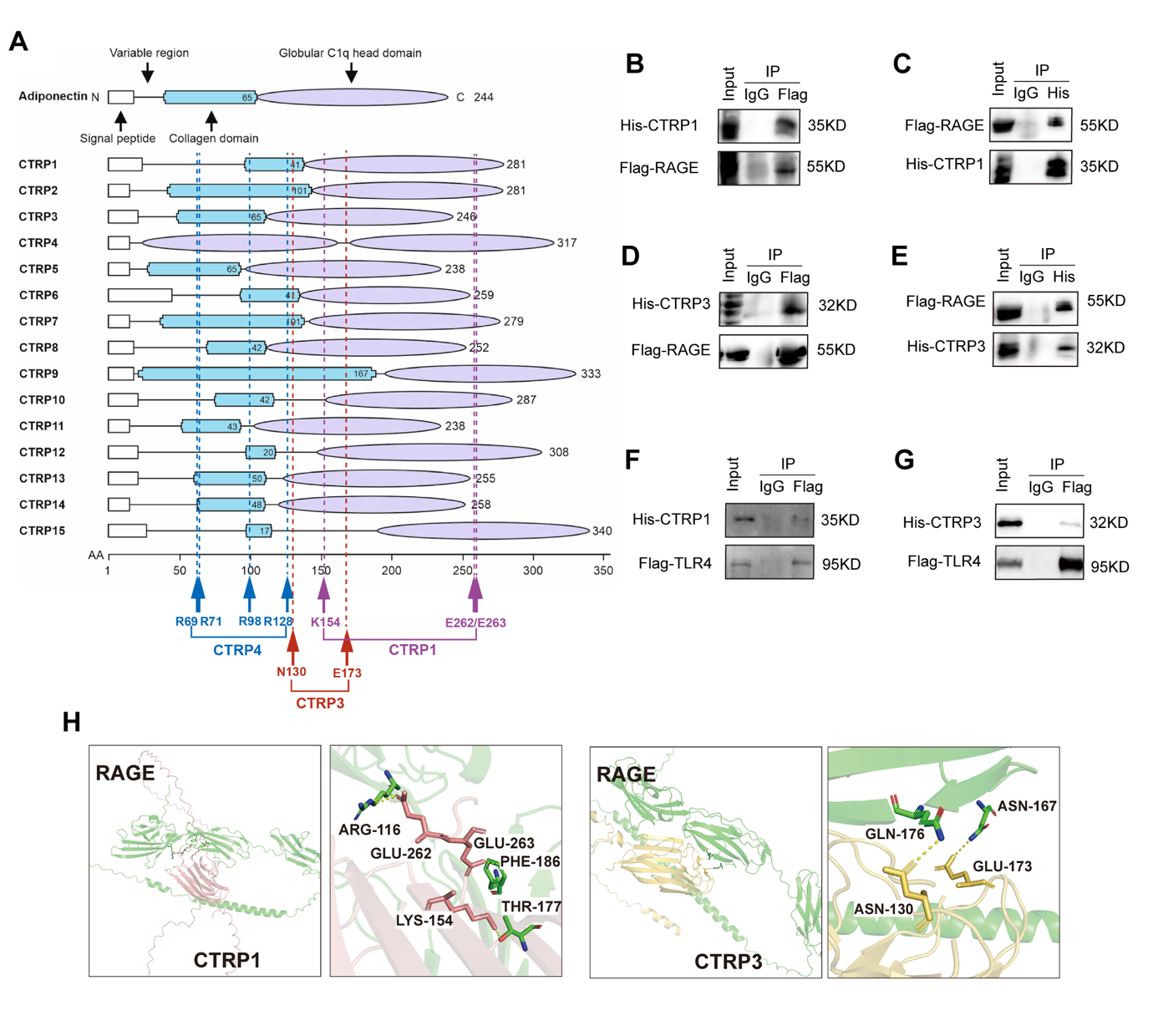
Supplementary Figure 8. CTRP1 and CTRP3 interact with RAGE and TLR4.**

A, Schematic representation of domain architecture of the CTRP family members (CTRP1–CTRP15), including the N-terminal signal peptide, variable region, collagen-like domain, and the C-terminal globular C1q head domain. CTRP4 is distinguished by the presence of two globular C1q head domains. Dashed lines indicate the positions of residues implicated in receptor engagement. Blue dashed lines denote CTRP4 residues R69, R71, R98, and Y128 within C1q head domain 1 (D1; aa 25–162), purple dashed lines denote CTRP1 residues K154 and E262/E263, and red dashed lines denote CTRP3 residues N130 and E173, which were predicted by molecular docking to contribute to RAGE interaction.

B–G, HEK293 cells were co-transfected with His-CTRP1 or His-CTRP3 and Flag-RAGE or Flag-TLR4, and co-immunoprecipitation experiments were performed with anti-Flag or anti-His antibodies, followed by Western blot analysis.

H, Potential RAGE-interacting residues in CTRP1 and CTRP3 were predicted using molecular docking.

ARG (R), arginine; ASN (N), asparagine; GLU (E), glutamic acid; LYS (K), lysine; CTRP, C1q/TNF-related protein; PHE, phenylalanine; RAGE, receptor for advanced glycation end products; THR, threonine; and TLR4, Toll-like receptor 4.

**
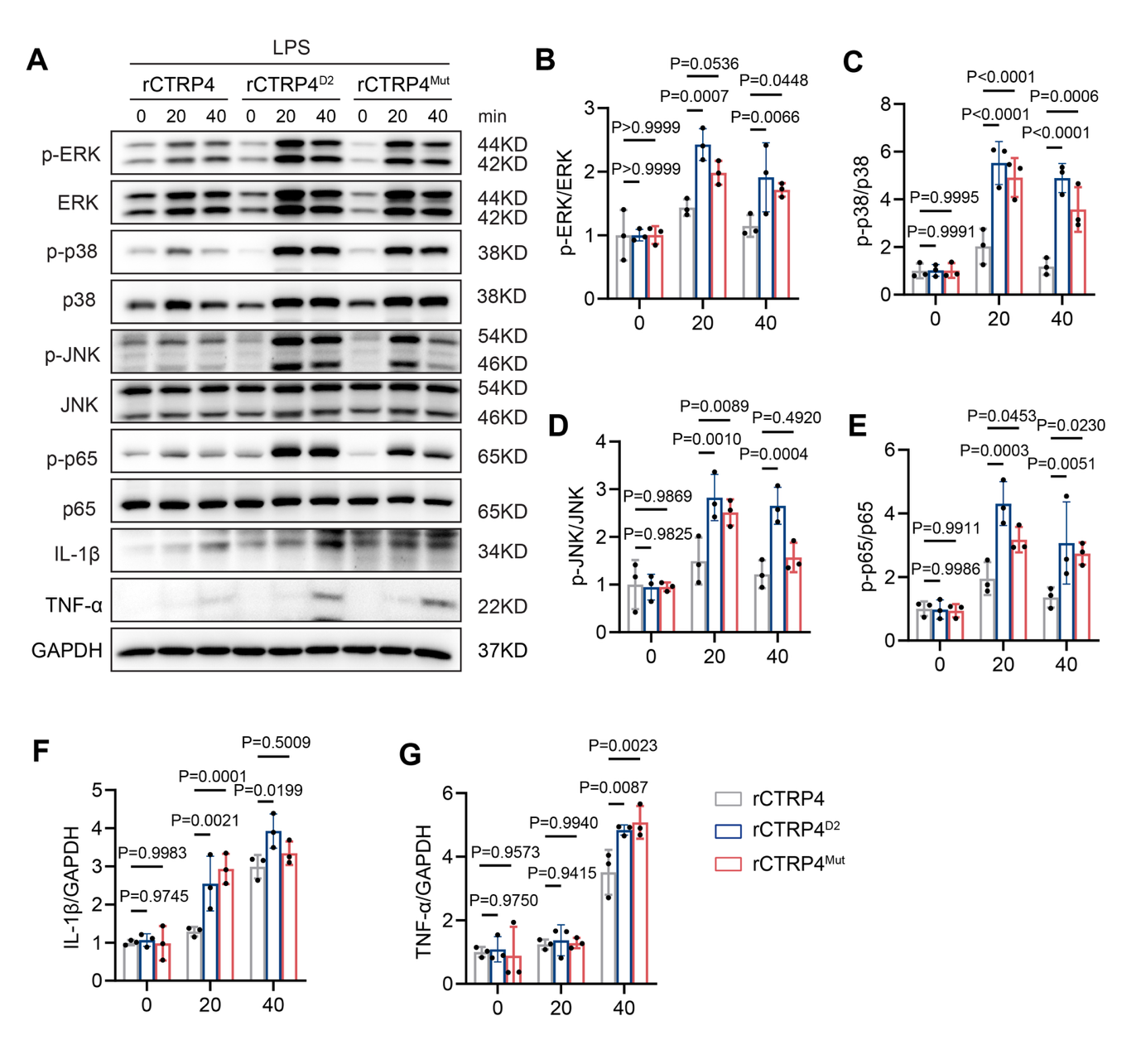
Supplementary Figure 9. Compared with wide-type CTRP4, CTRP mutants CTRP4^D2^ and CTRP4^Mut^ fail to inhibit LPS-induced inflammation in macrophages.**

A, BMDMs from CTRP4^−/−^ mice were pretreated with recombinant proteins CTRP4, CTRP4^D2^ (1 μg/mL), and CTRP4^Mut^ (1 μg/mL) for 3 hours, followed by stimulation with LPS (50 ng/mL) for 0, 20, and 40 minutes. Western blot was used to detect the phosphorylation levels of ERK, p38, JNK, and p65, as well as the protein levels of IL-1β and TNF-α.

B-G. Quantitative analysis of the data in A (n=3).

Data are presented as mean ± SD. Data in **B** to **G** were analyzed by using one-way ANOVA followed by Bonferroni post hoc tests. CTRP4, C1q/TNF-related protein 4; LPS, lipopolysaccharide; p-ERK, phosphorylated extracellular signal-regulated kinase; p-JNK, phosphorylated c-Jun N-terminal kinase; p-p38, phosphorylated p38 mitogen-activated protein kinase; p-p65, phosphorylated p65 subunit of NF-κB; IL-1β, interleukin-1 beta; and TNF-α, tumor necrosis factor-alpha.**
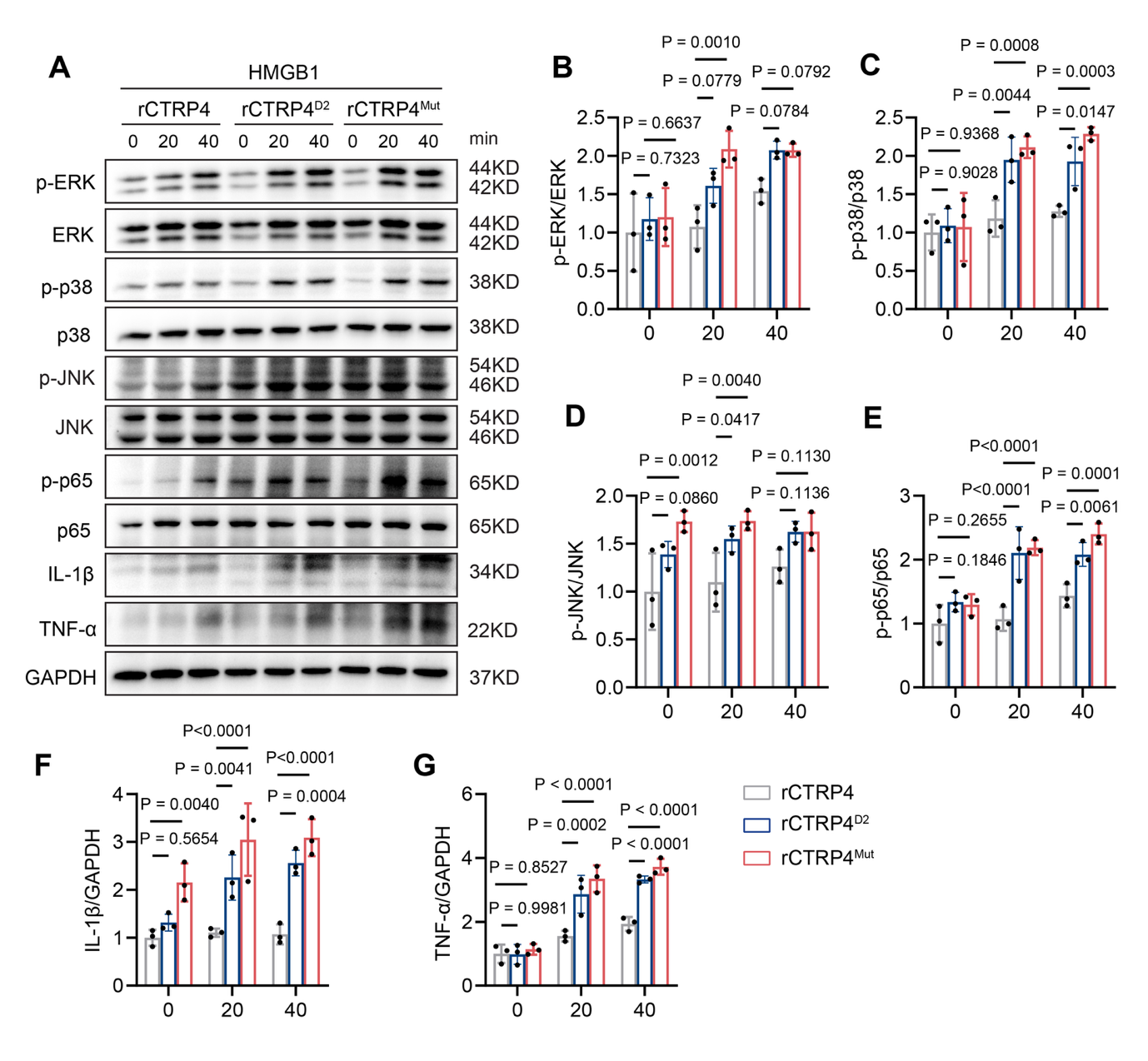
 Supplementary Figure 10. Compared with wide-type CTRP4, CTRP mutant CTRP4^D2^ and CTRP4^Mut^ fail to inhibit HMGB1-induced inflammation in macrophages.**

A, BMDMs from CTRP4^−/−^ mice were pretreated with recombinant proteins CTRP4, CTRP4^D2^, and CTRP4^Mut^ (1 μg/mL) for 3 hours, followed by stimulation with HMGB1 (5 μg/mL) for 0, 20, and 40 minutes. Western blot was used to detect the phosphorylation levels of ERK, p38, JNK, and p65, as well as the protein levels of IL-1β and TNF-α.

B-G. Quantitative analysis of the data in A (n=3).

Data are presented as mean ± SD. Data in **B** to **G** were analyzed by using one-way ANOVA followed by Bonferroni post hoc tests. CTRP4, C1q/TNF-related protein 4; HMGB1, high mobility group box 1; IL-1β, interleukin-1 beta; p-ERK, phosphorylated extracellular signal-regulated kinase; p-JNK, phosphorylated c-Jun N-terminal kinase; p-p38, phosphorylated p38 mitogen-activated protein kinase; p-p65, phosphorylated p65 subunit of NF-κB; and TNF-α, tumor necrosis factor-alpha.

**
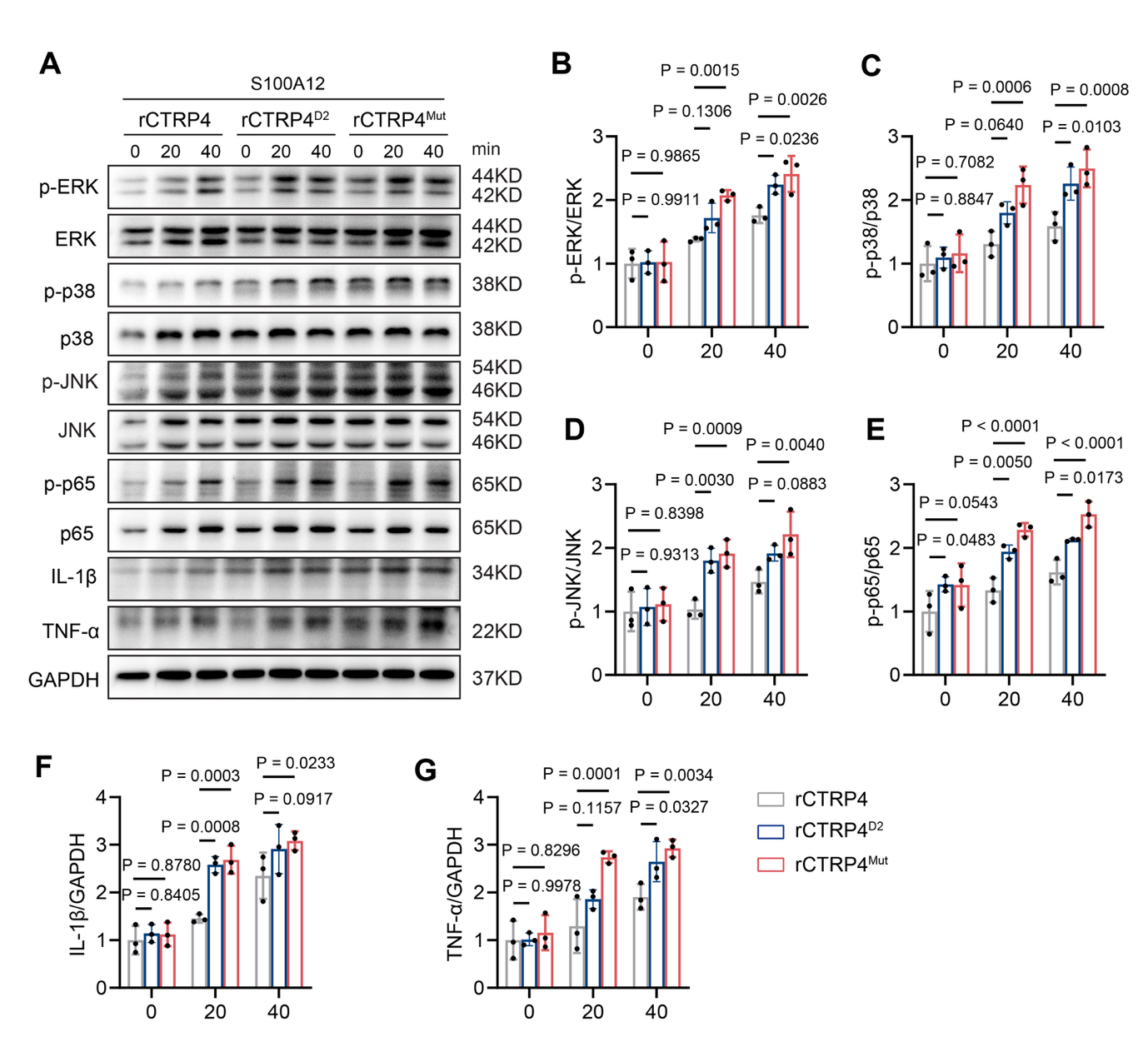
 Supplementary Figure 11. Compared with wide-type CTRP4,** **CTRP4 mutant CTRP4^D2^ and CTRP4^Mut^ fail to inhibit S100A12-induced inflammation in macrophages.**

A, BMDMs from CTRP4^−/−^ mice were pretreated with recombinant proteins CTRP4, CTRP4^D2^, and CTRP4^Mut^ (1 μg/mL) for 3 hours, followed by stimulation with S100A12 (50 μg/mL) for 0, 20, and 40 minutes. Western blot was used to detect the phosphorylation levels of ERK, p38, JNK, and P65, as well as the protein levels of IL-1β and TNF-α.

B-G. Quantitative analysis of the data in A (n=3).

Data are presented as mean ± SD. Data in **B** to **G** were analyzed by using one-way ANOVA followed by Bonferroni post hoc tests. CTRP4, C1q/TNF-related protein 4; IL-1β, interleukin-1 beta; p-ERK, phosphorylated extracellular signal-regulated kinase; p-JNK, phosphorylated c-Jun N-terminal kinase; p-p38, phosphorylated p38 mitogen-activated protein kinase; p-p65, phosphorylated p65 subunit of NF-κB; S100A12, S100 calcium-binding protein A12; and TNF-α, tumor necrosis factor-alpha.


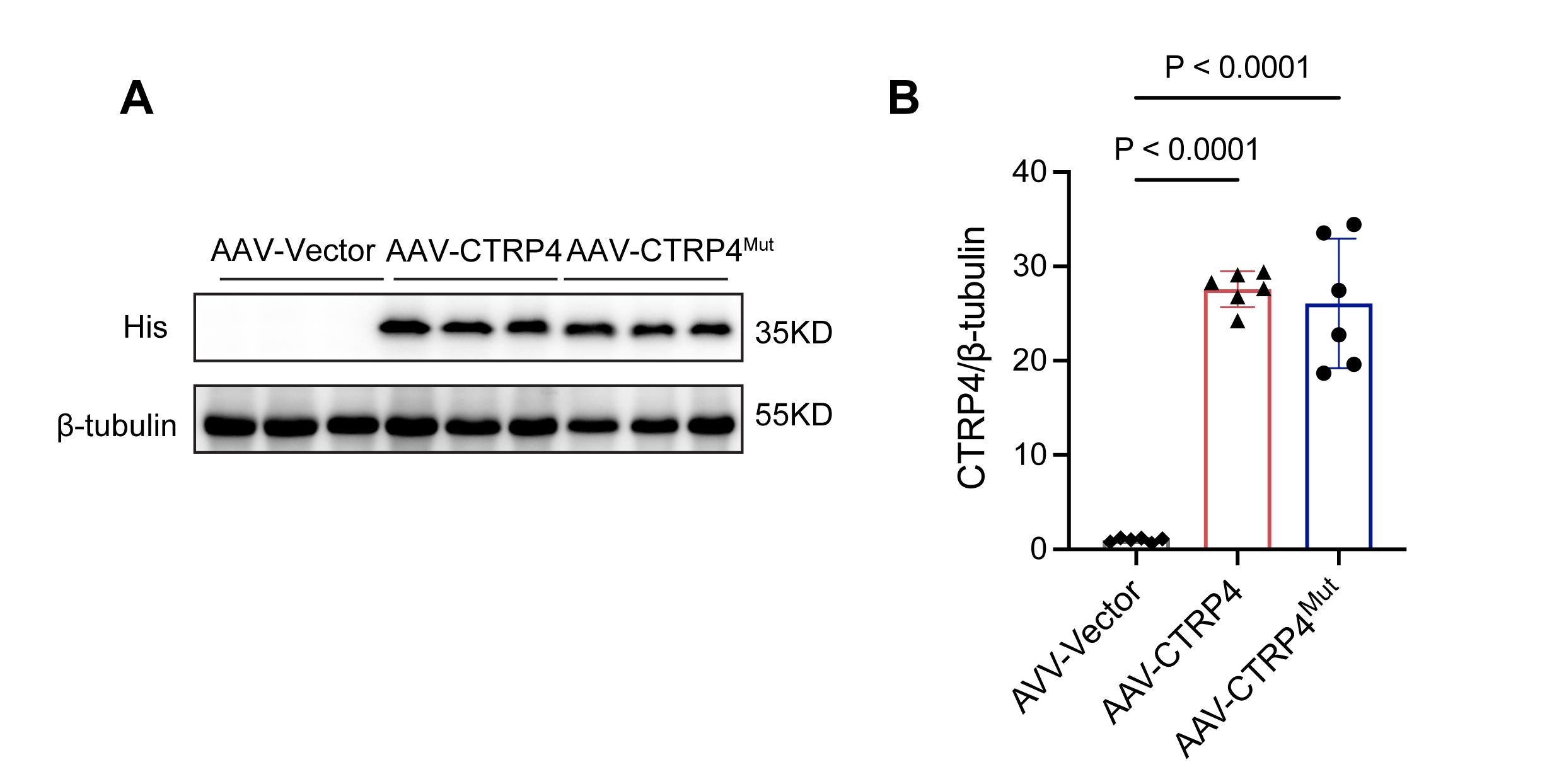


**Supplementary Figure 12. Adeno-associated viruses (AAV)-mediated overexpression of CTRP4 in adipose tissue.**

A, Male CTRP4⁻/⁻ mice (6–8 weeks old) were injected intraperitoneally with adeno-associated viruses overexpressing wild-type CTRP4 (AAV-CTRP4), mutant CTRP4 (AAV-CTRP4^Mut^), or control vector (AAV-Vector) for 2 weeks. CTRP4 protein levels in mesenteric adipose tissue (MAT) were assessed by Western blot.

B, Quantitative analysis of the data in A (n=6).

Data are presented as mean ± SD. Data in **B** was analyzed by using one-way ANOVA followed by Bonferroni post hoc tests. CTRP4, C1q/TNF-related protein 4
