# Supplementary material for: Decreased adipokine CTRP4 in CAD patients: CTRP4 attenuates atherosclerosis via inhibition of RAGE and TLR4
Source: Clin Transl Med. 2026 Feb 18;16(2):e70624. doi: 10.1002/ctm2.70624 (PMC12914348; doi:10.1002/ctm2.70624)

Full unedited gel of immunoblots (1/7)

Figure 1B

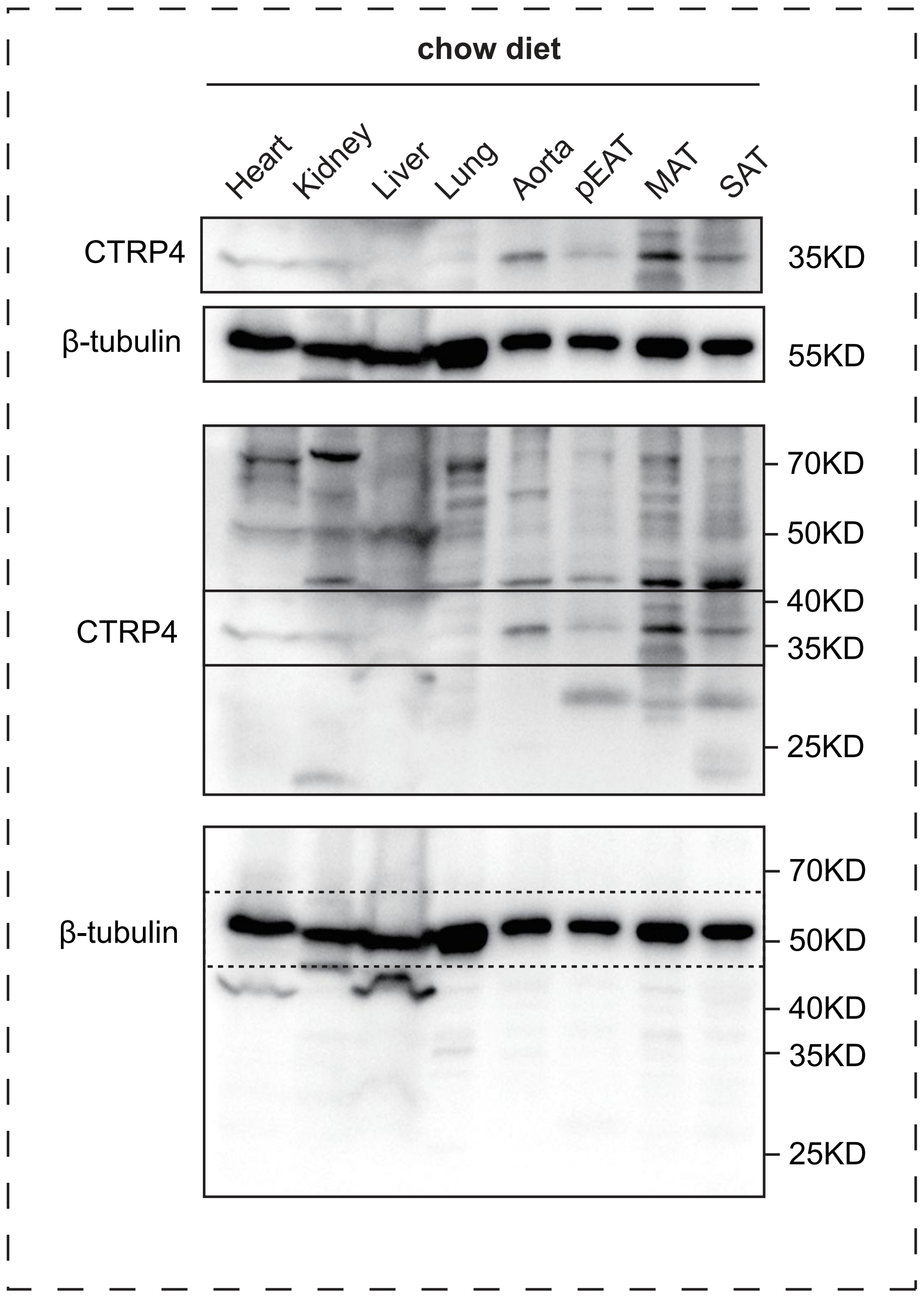

Figure 1D

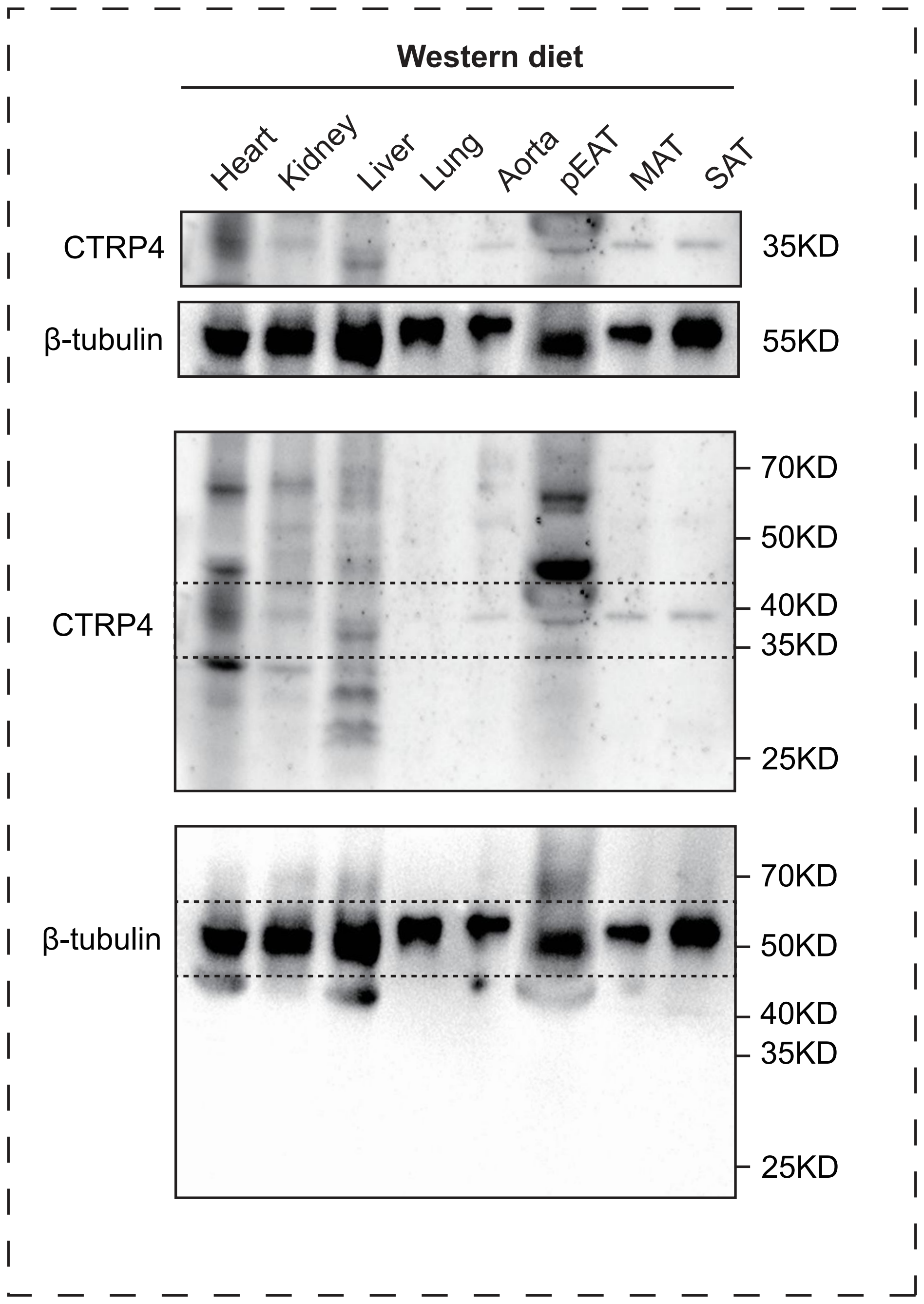

Figure 1F

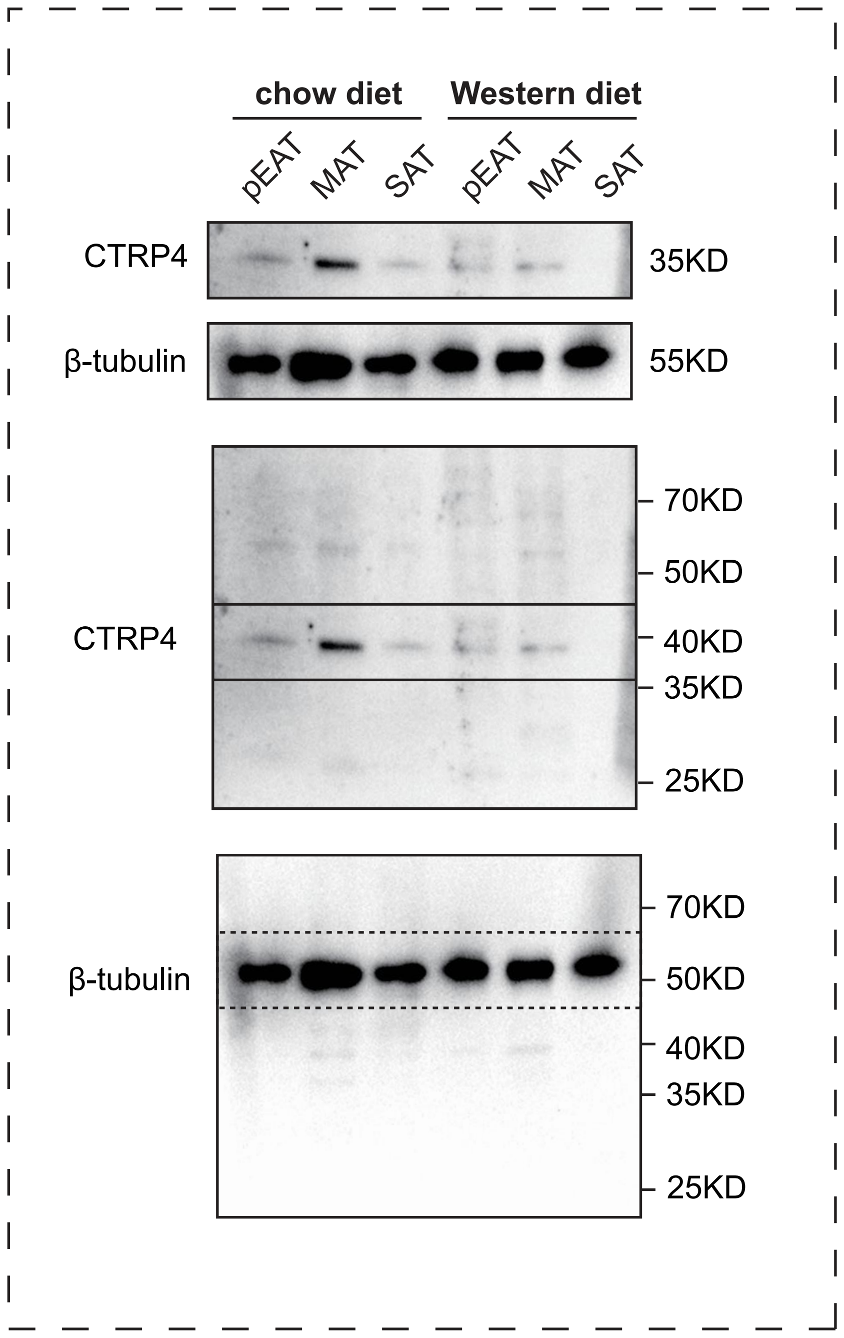

Figure 2D

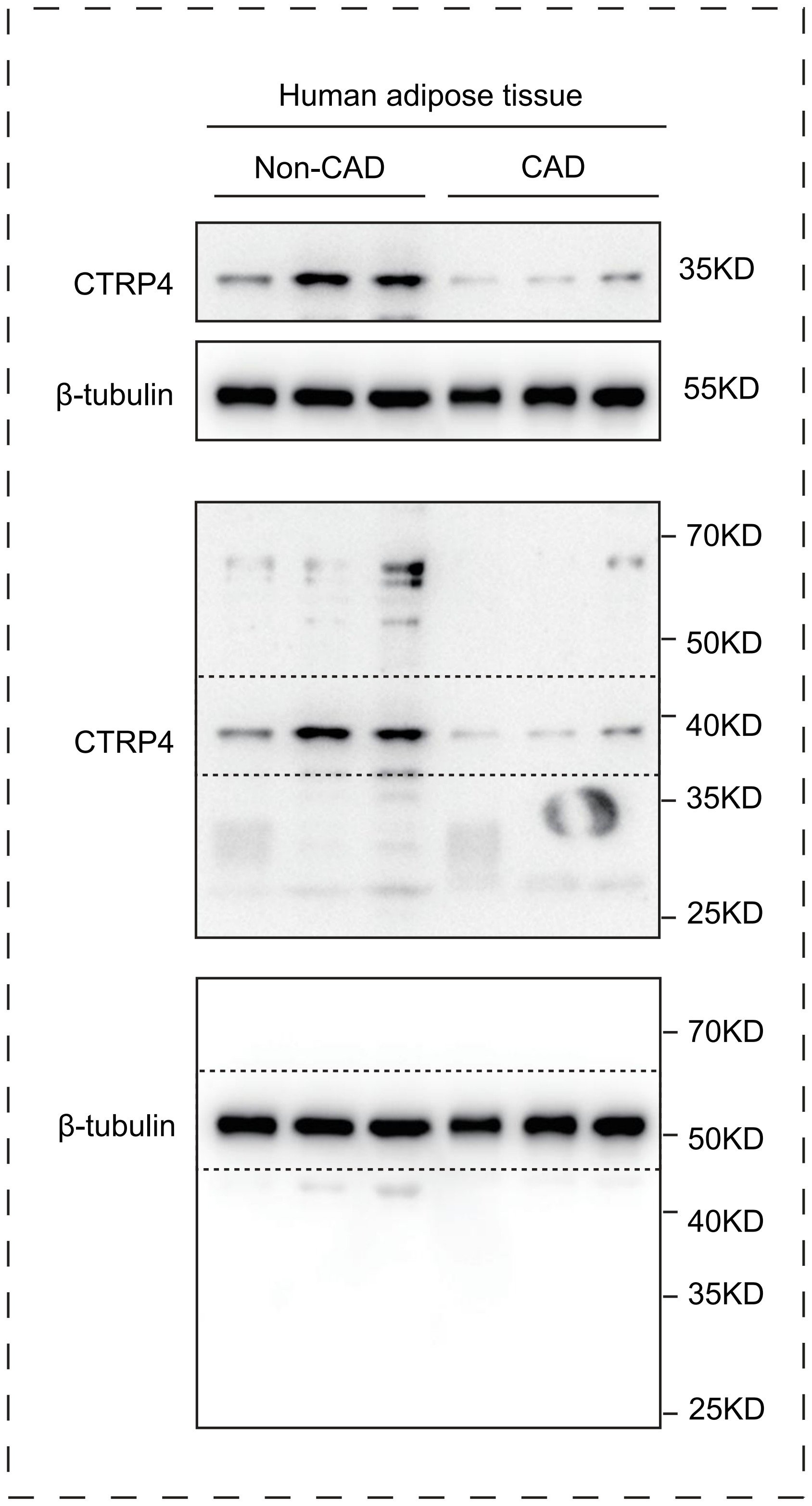

Figure 6D

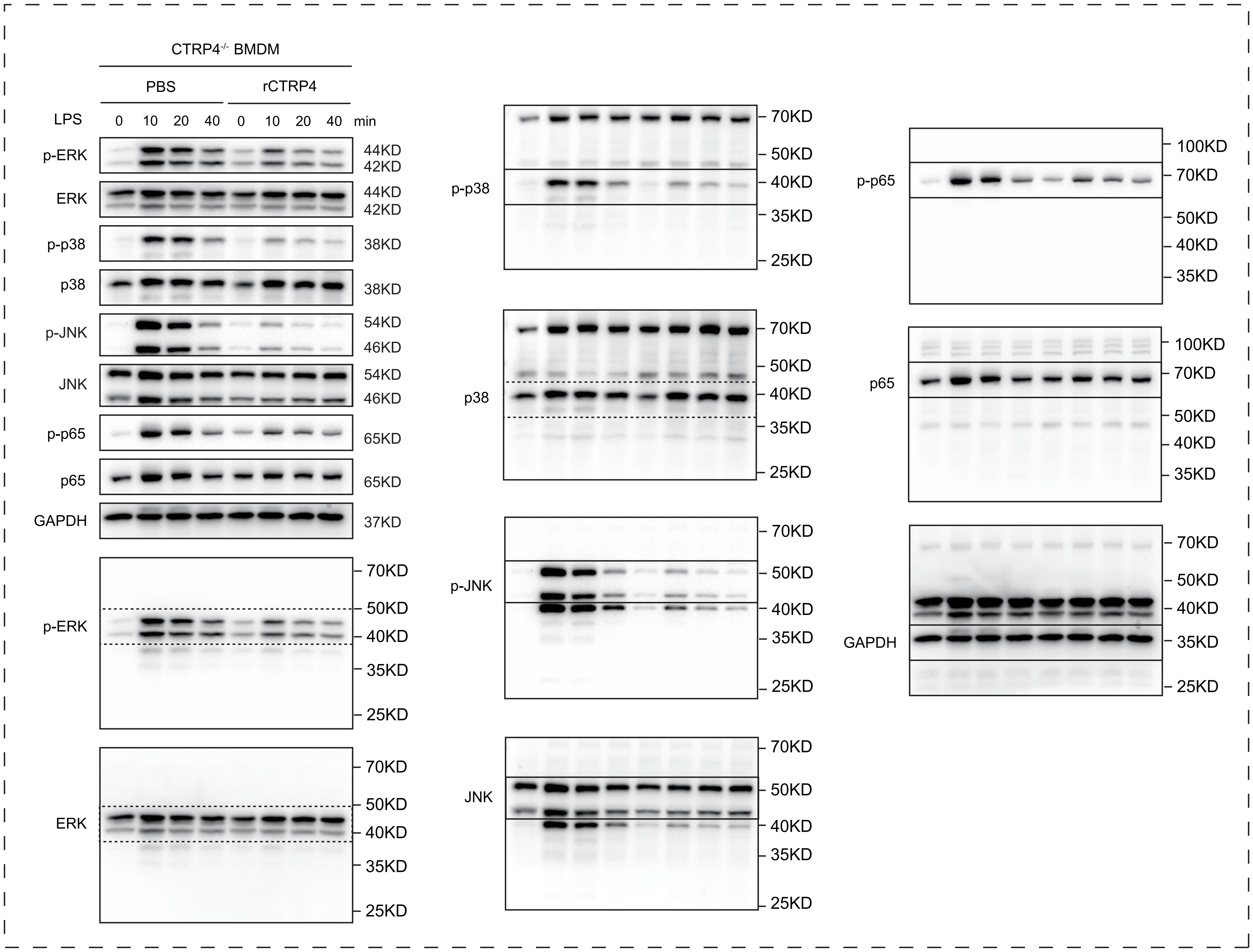

Full unedited gel of immunoblots (2/7)

Figure 6I

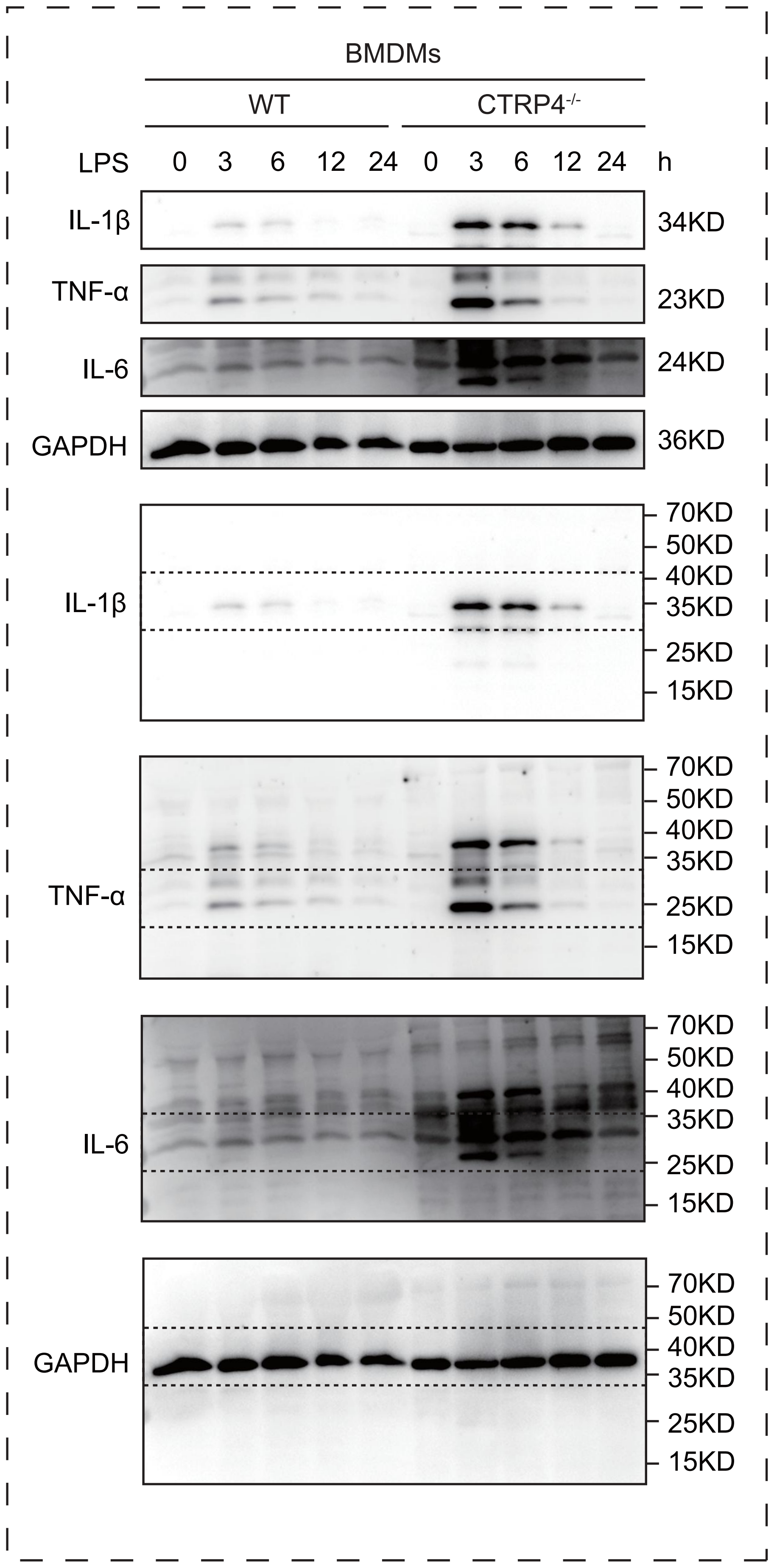

Figure 6M

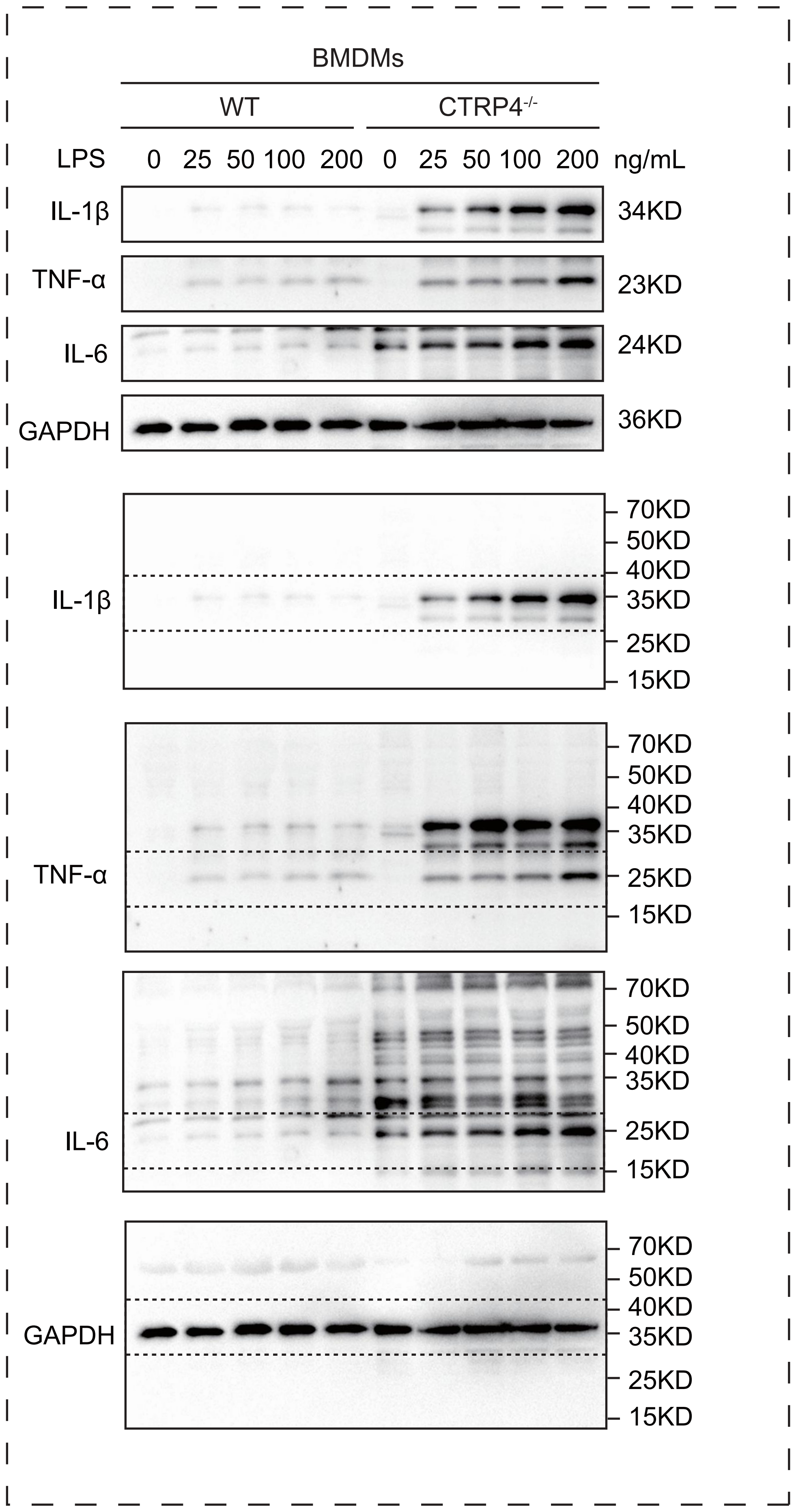

Figure 6Q

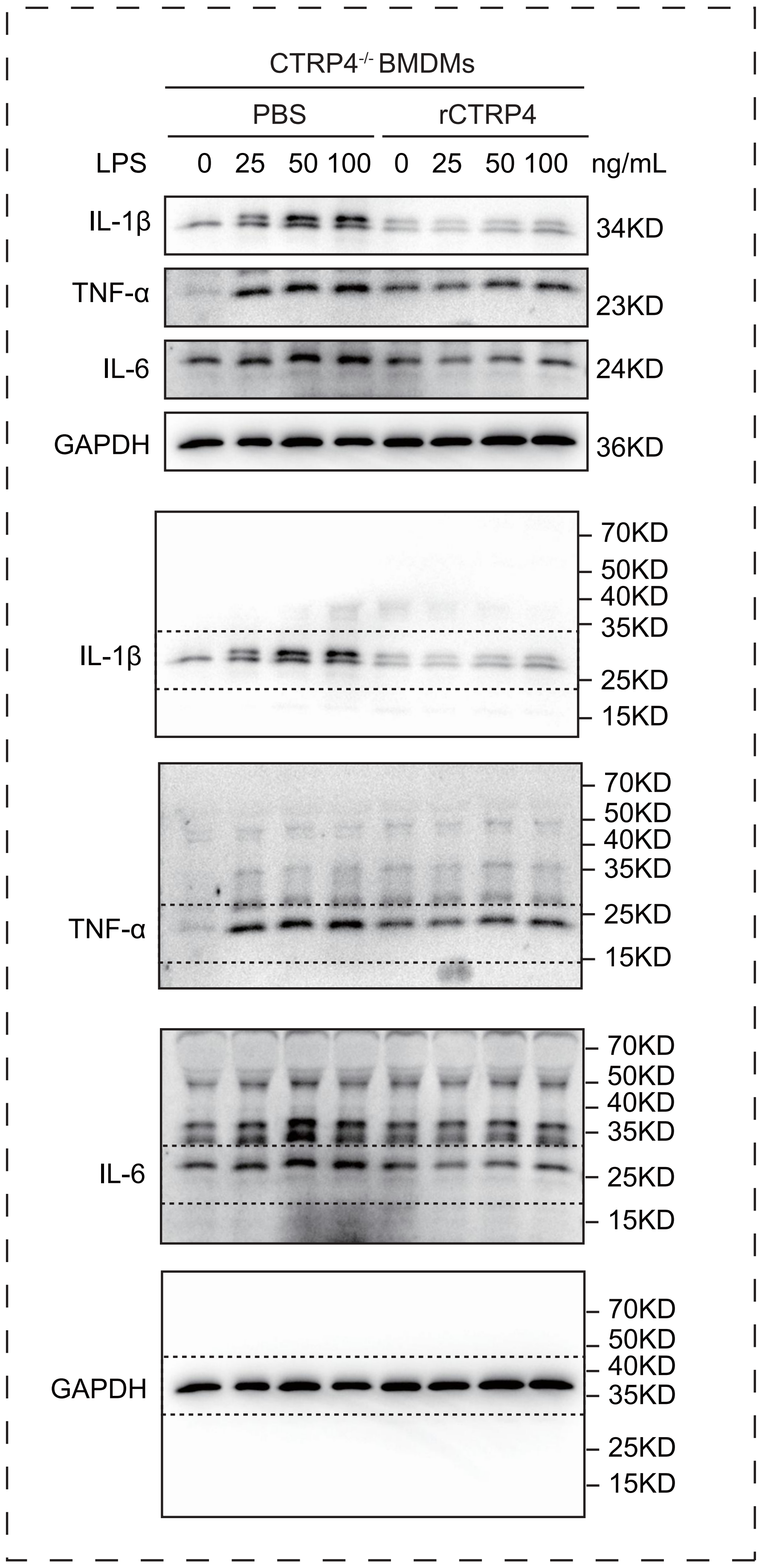

Figure 6U

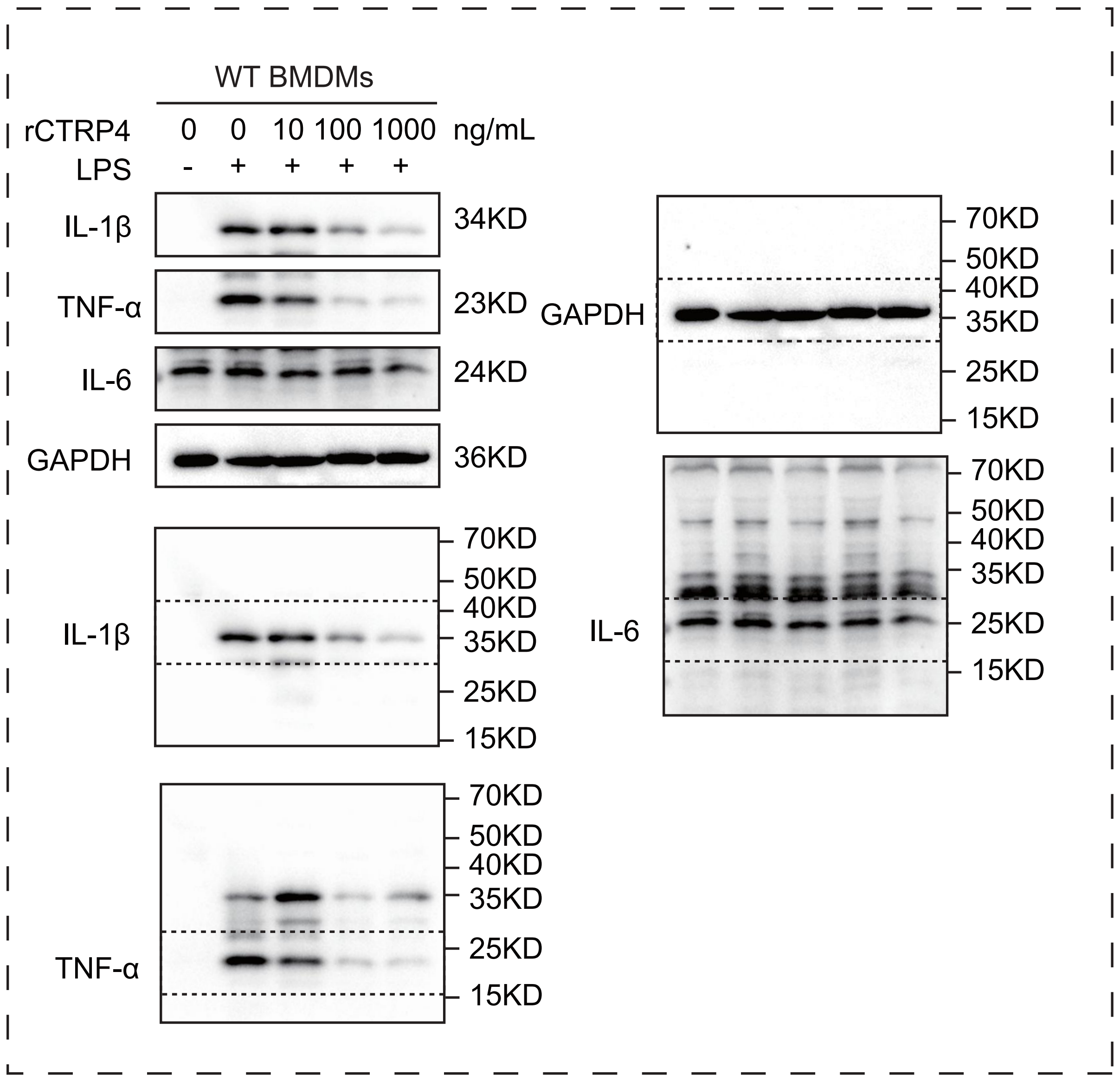

Figure 7H

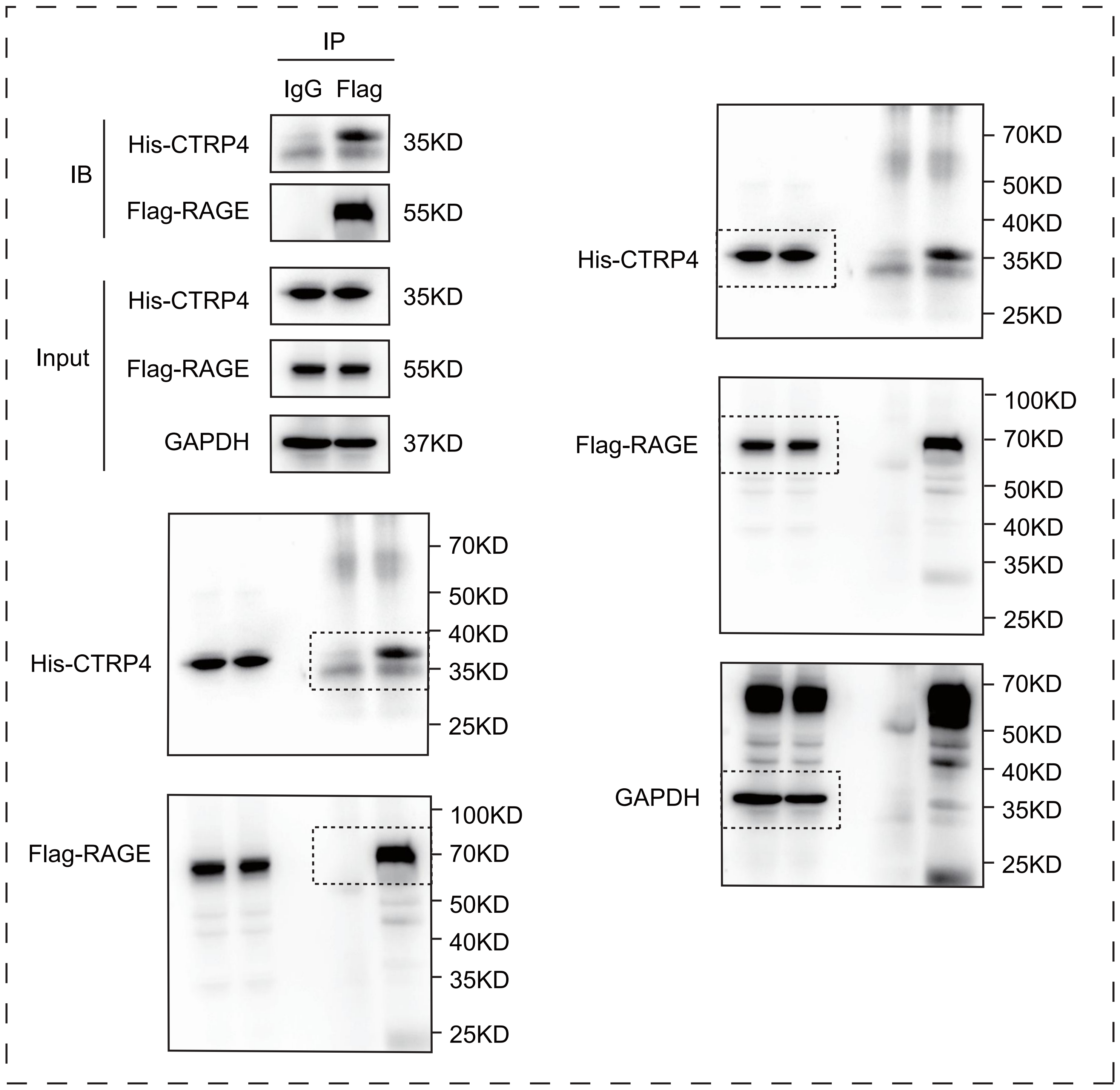

Full unedited gel of immunoblots (3/7)

Figure 7I

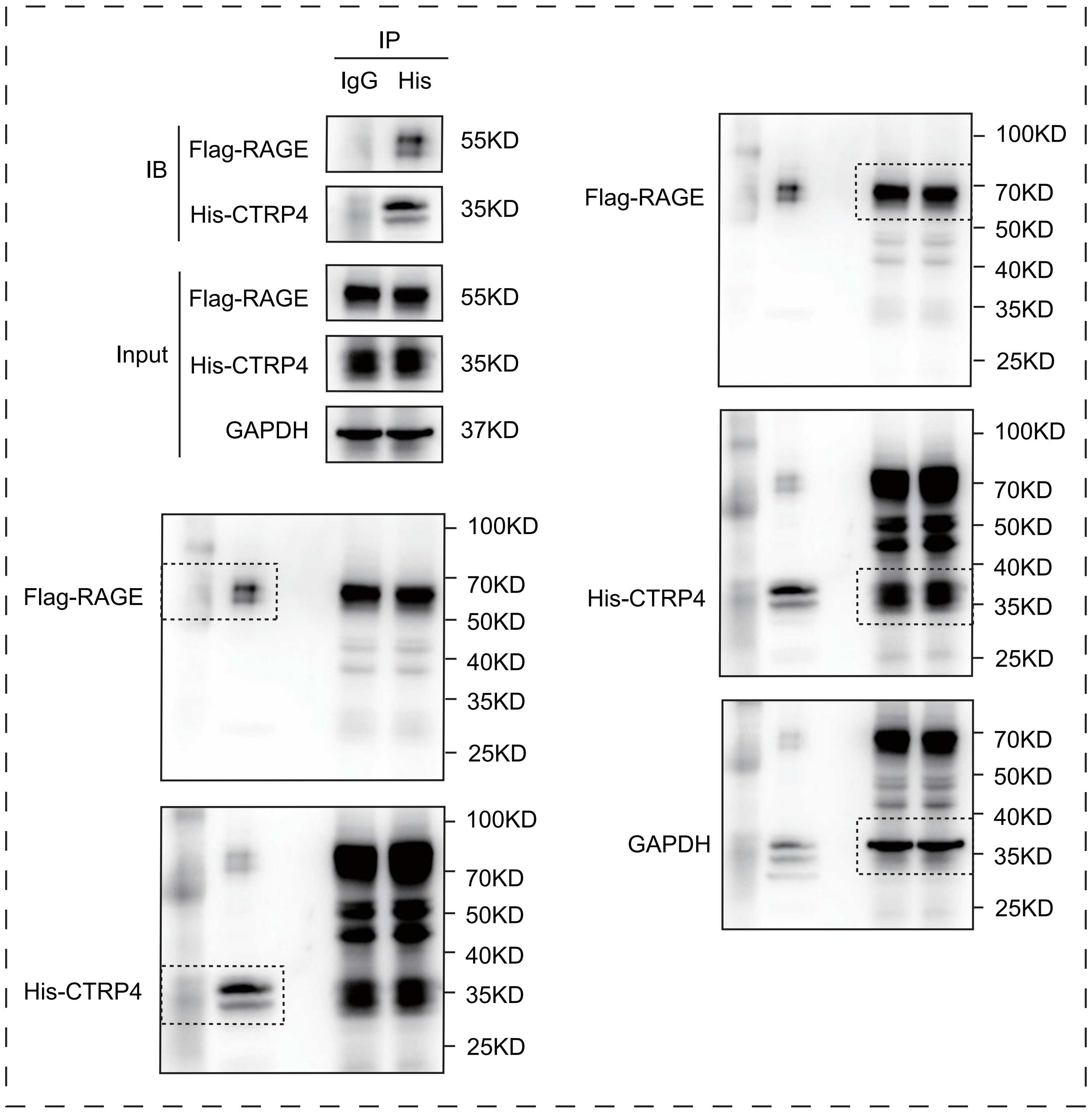

Figure 7J

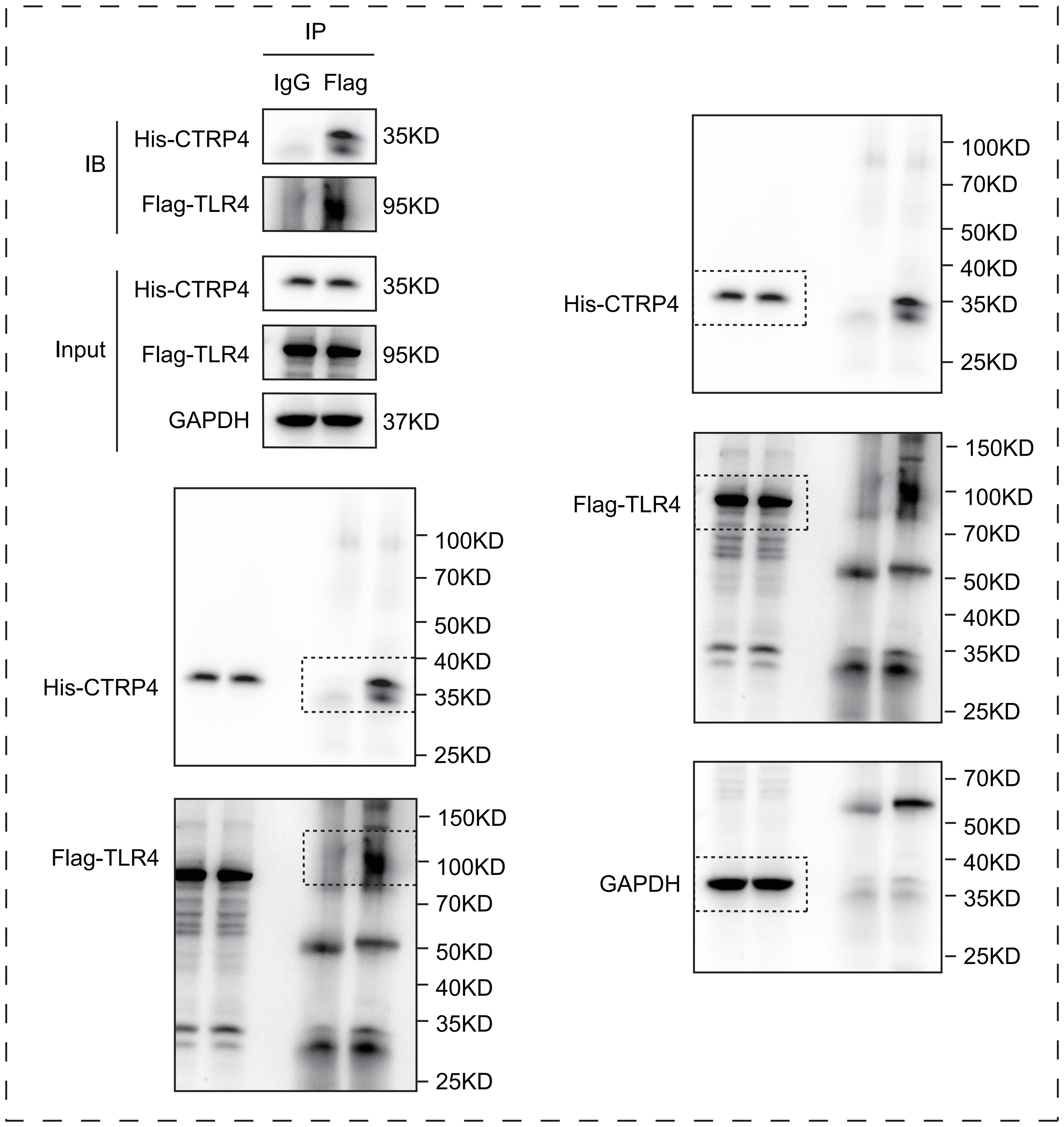

Figure 7K

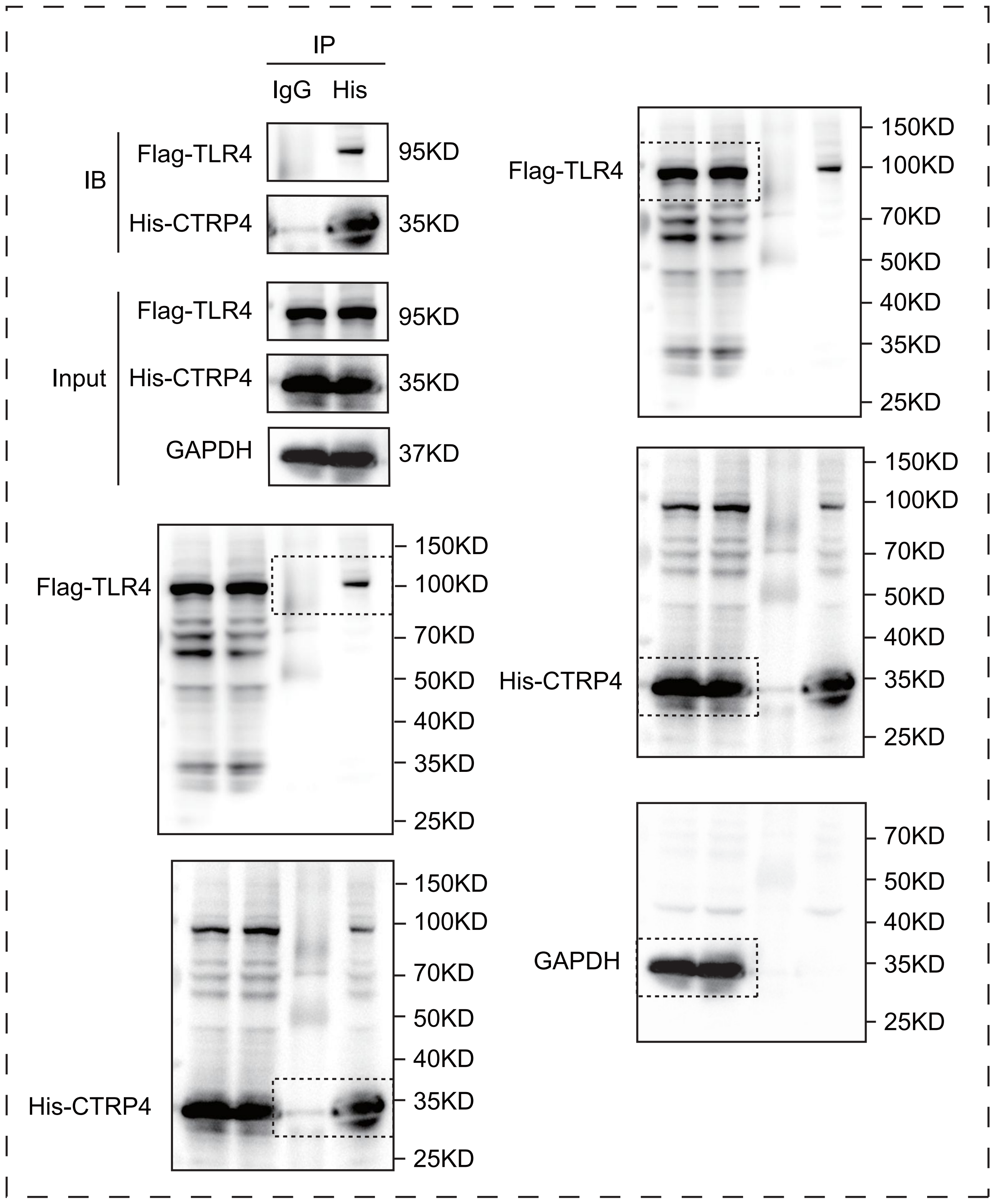

Figure 7P

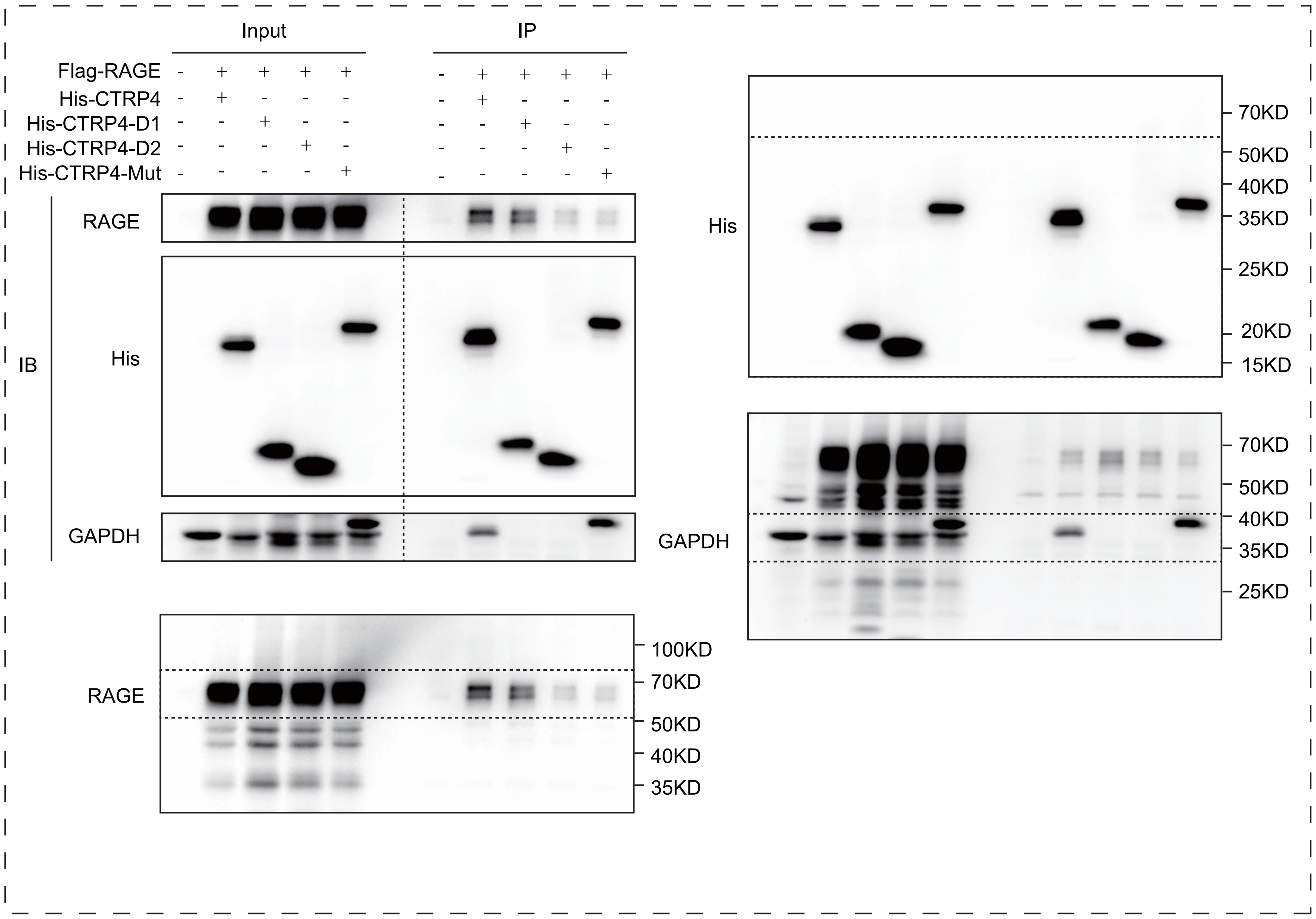

Full unedited gel of immunoblots (4/7)

Figure 7Q

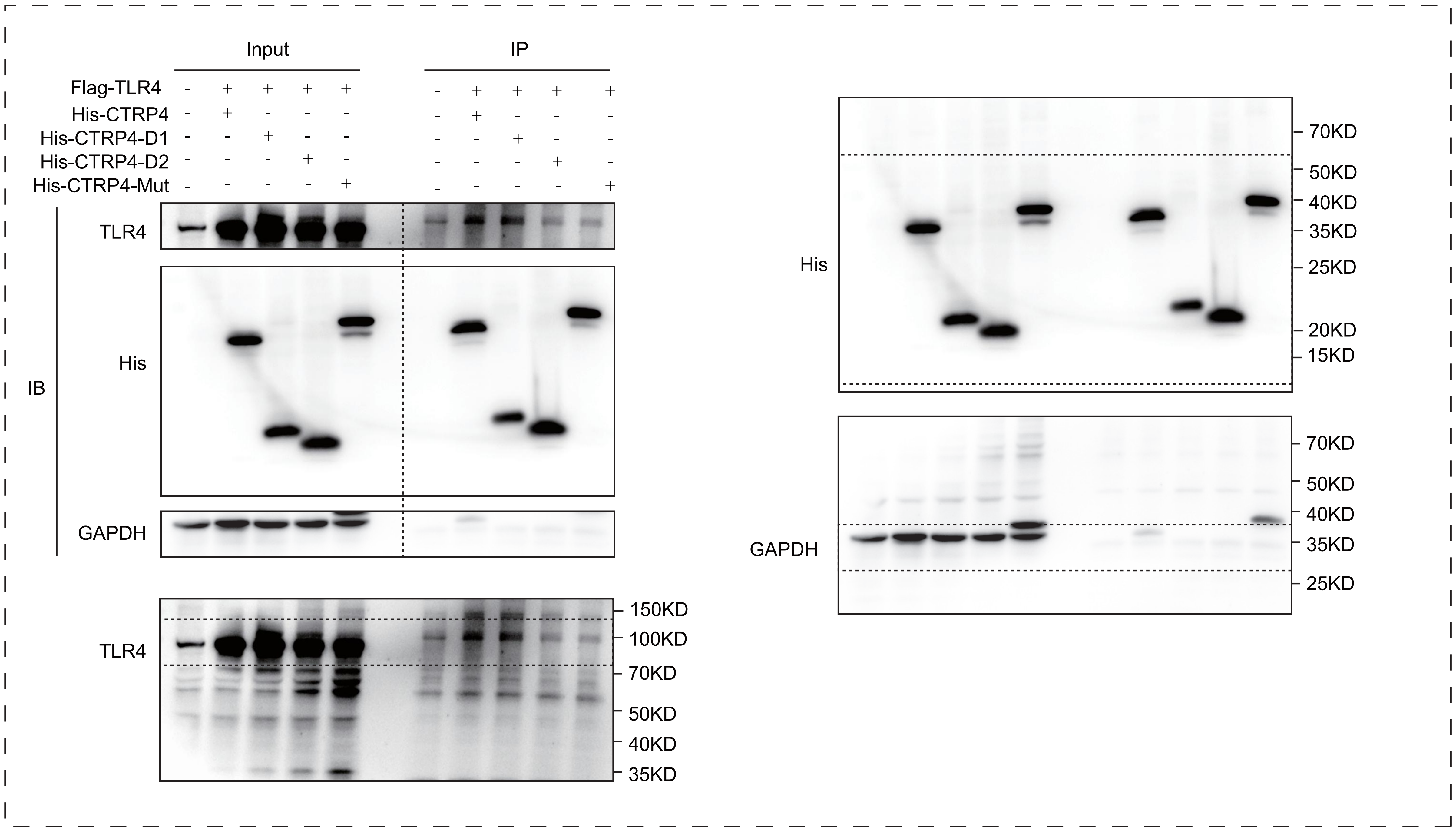

Figure S4D

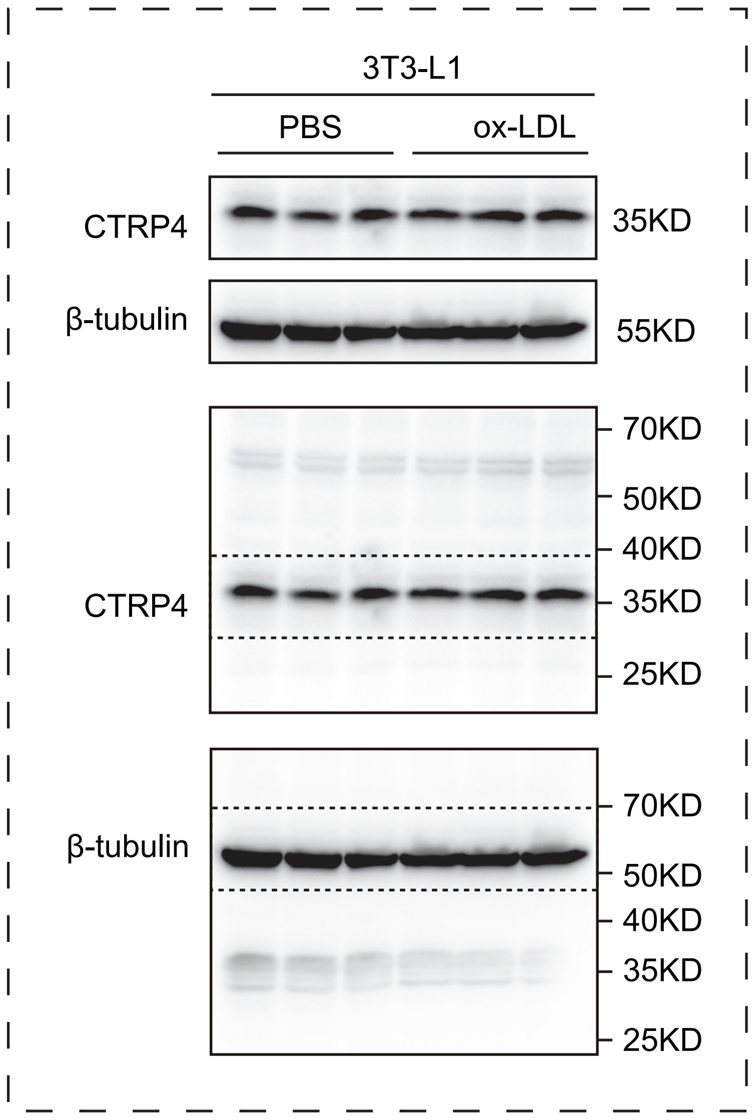

Figure S4E

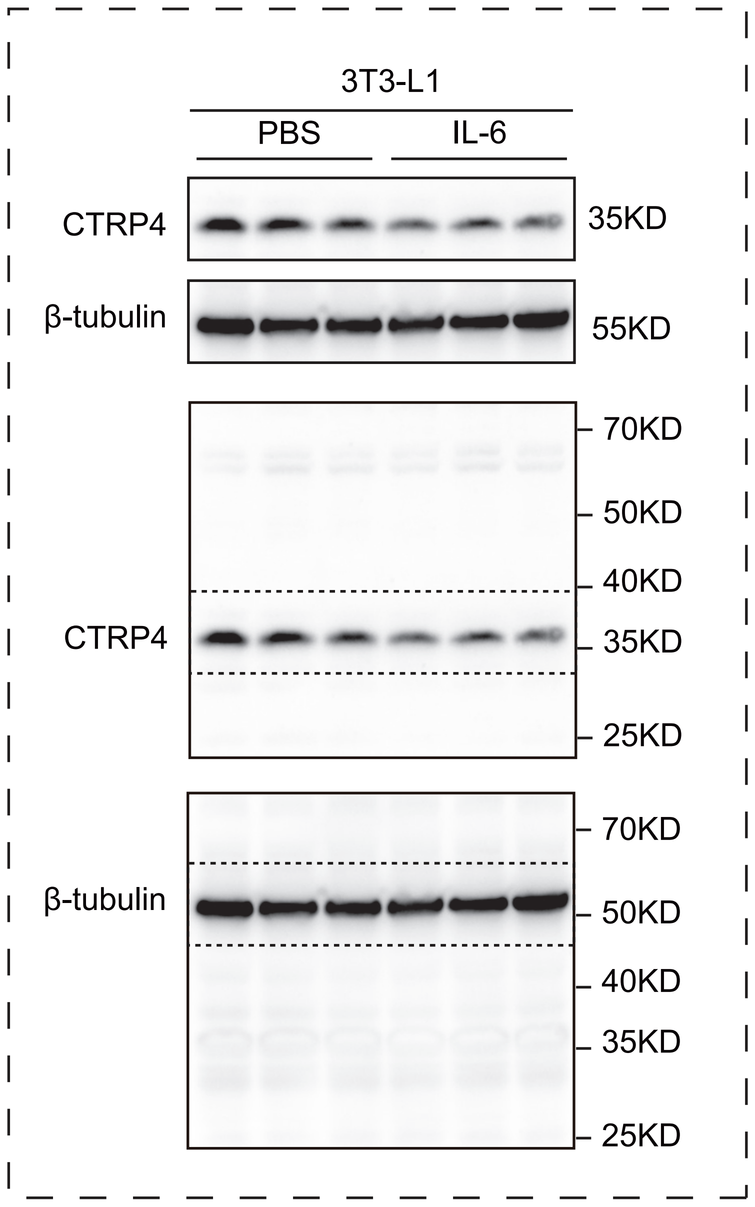

Figure S4F

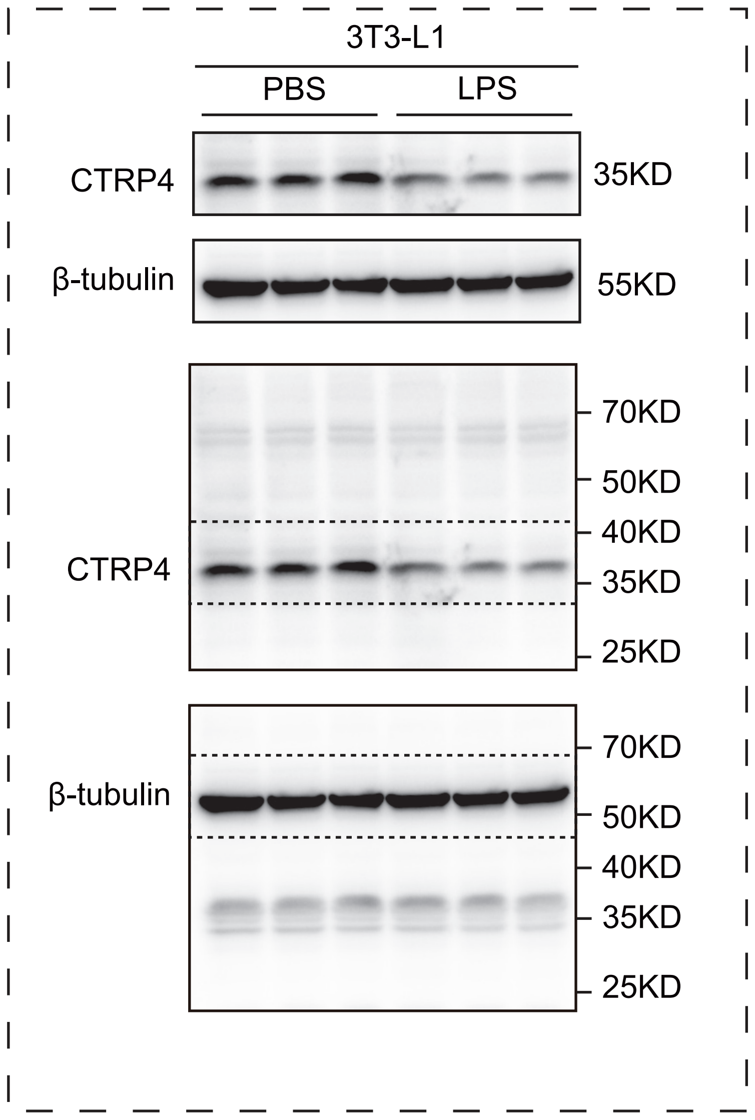

Figure S4G

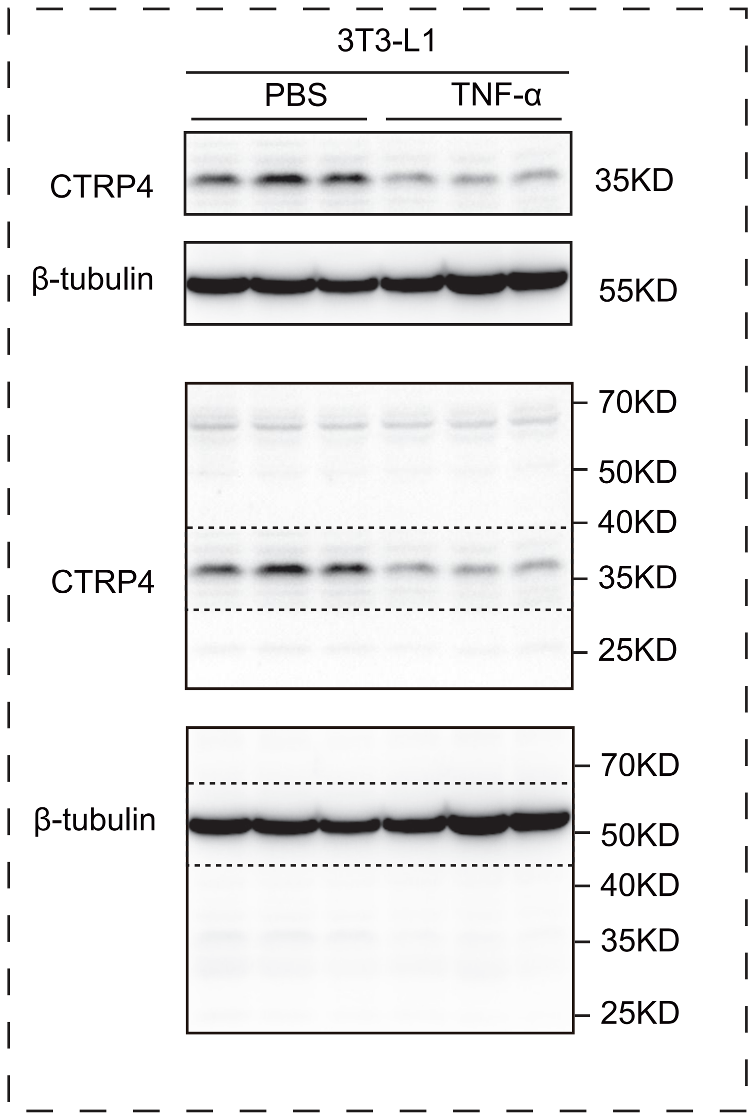

Figure S4H

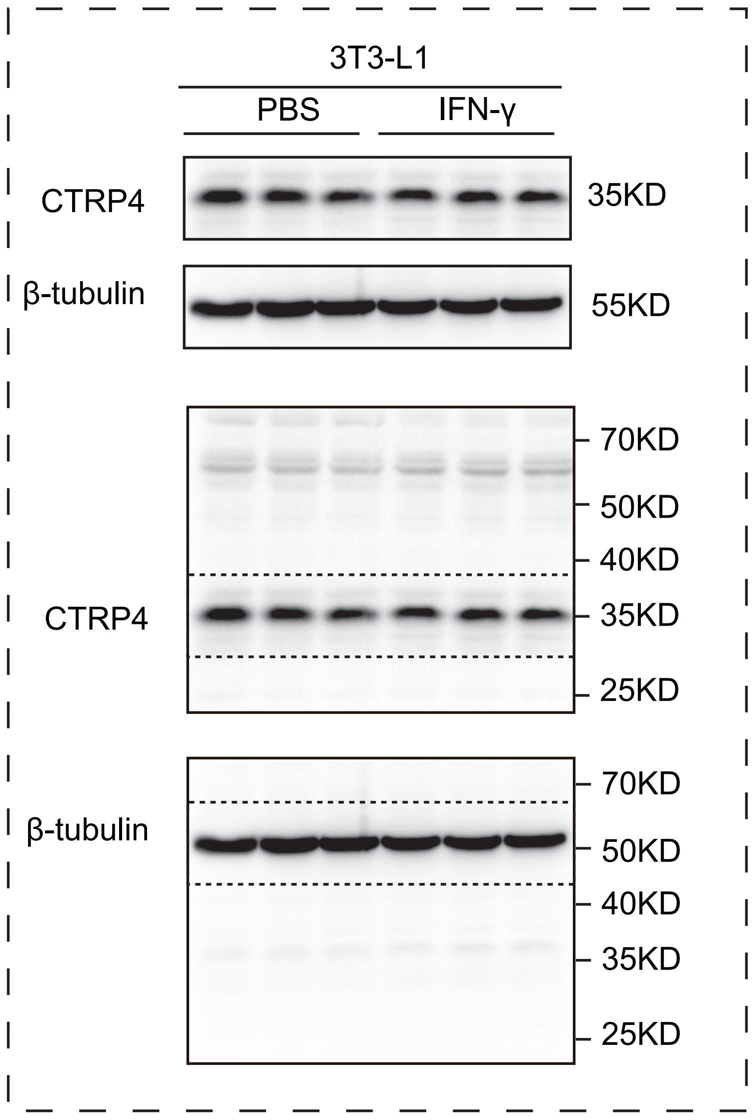

Figure S4I

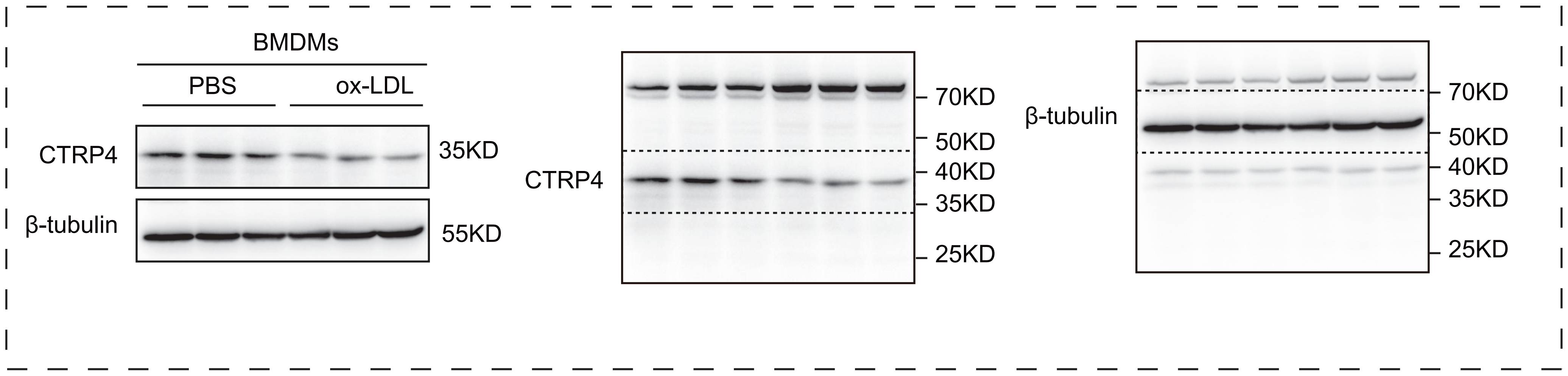

Full unedited gel of immunoblots (5/7)

Figure S4J

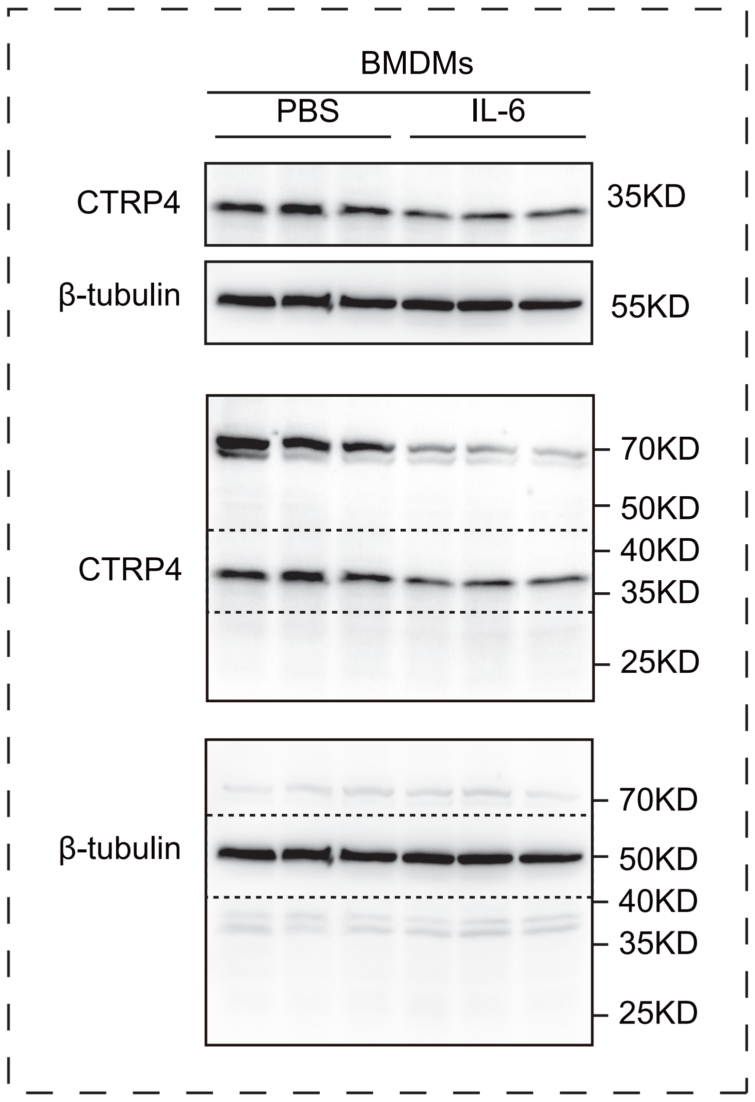

Figure S4K

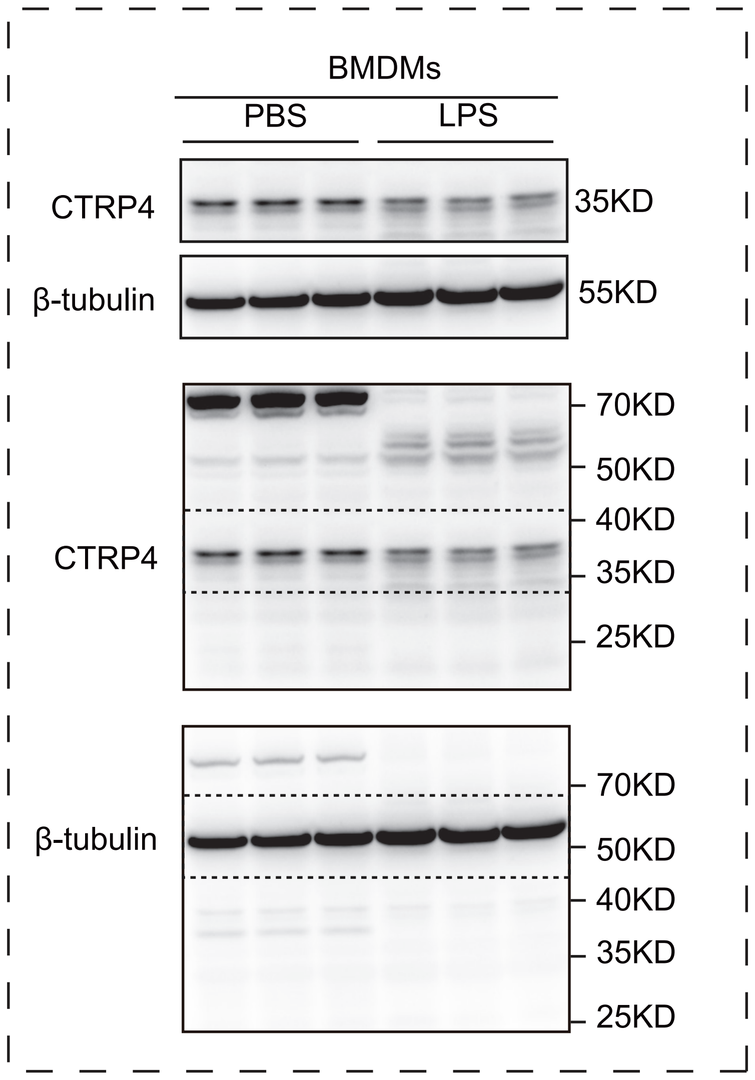

Figure S4L

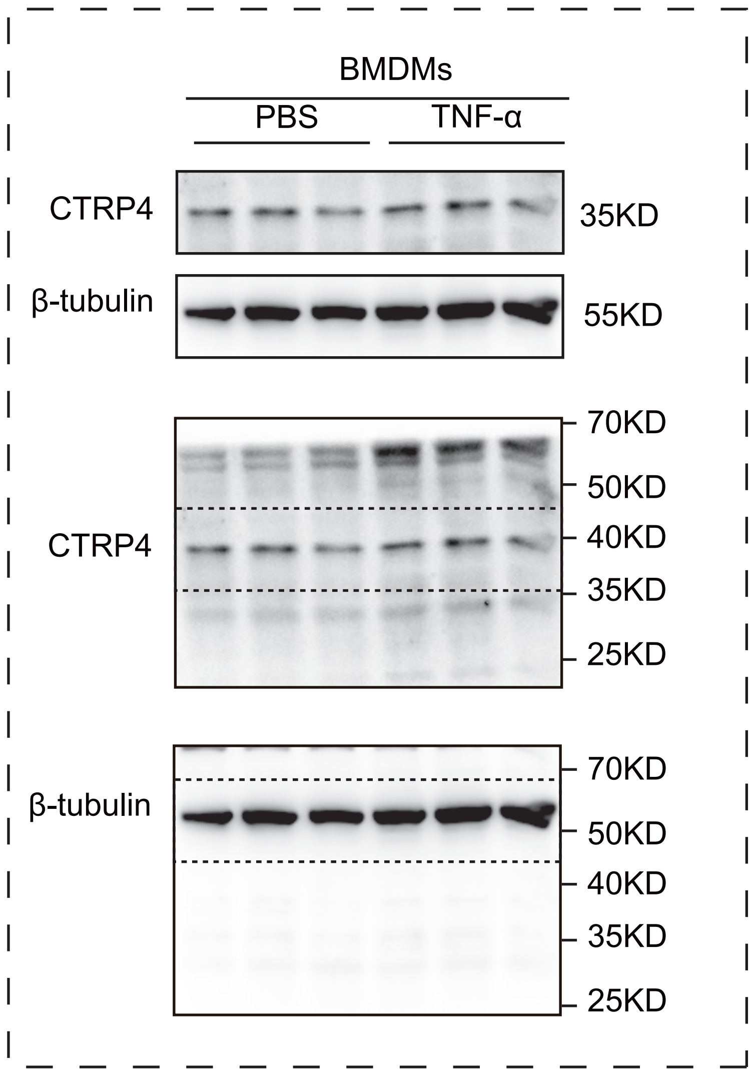

Figure S4M

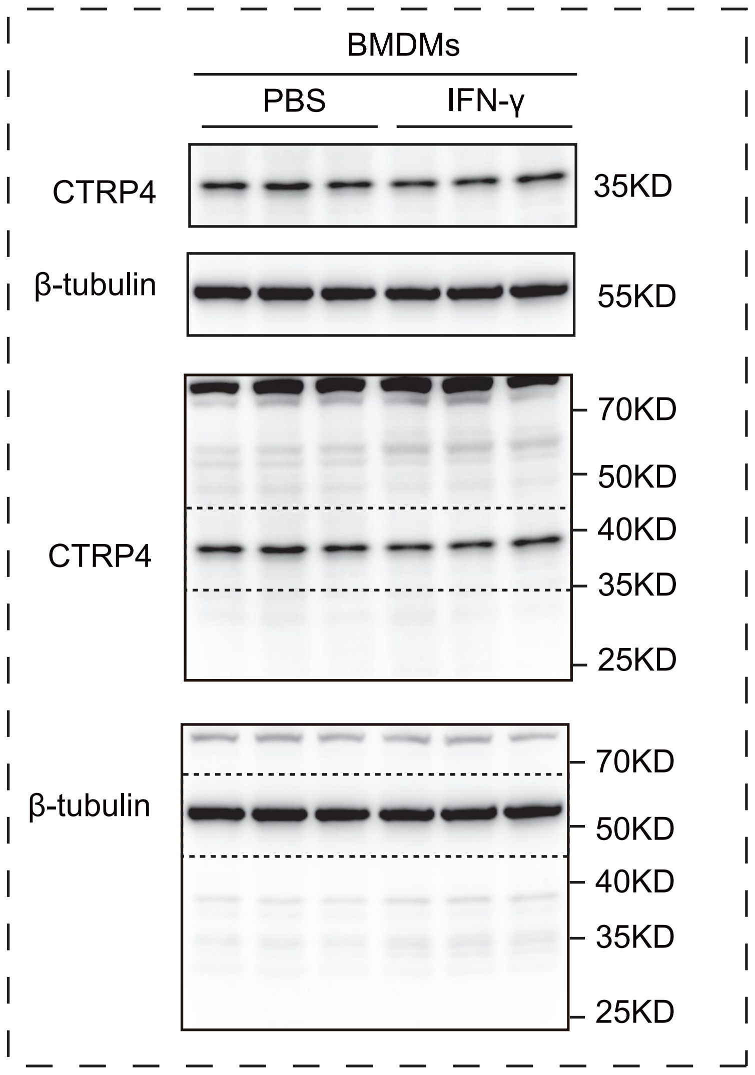

Figure S6A

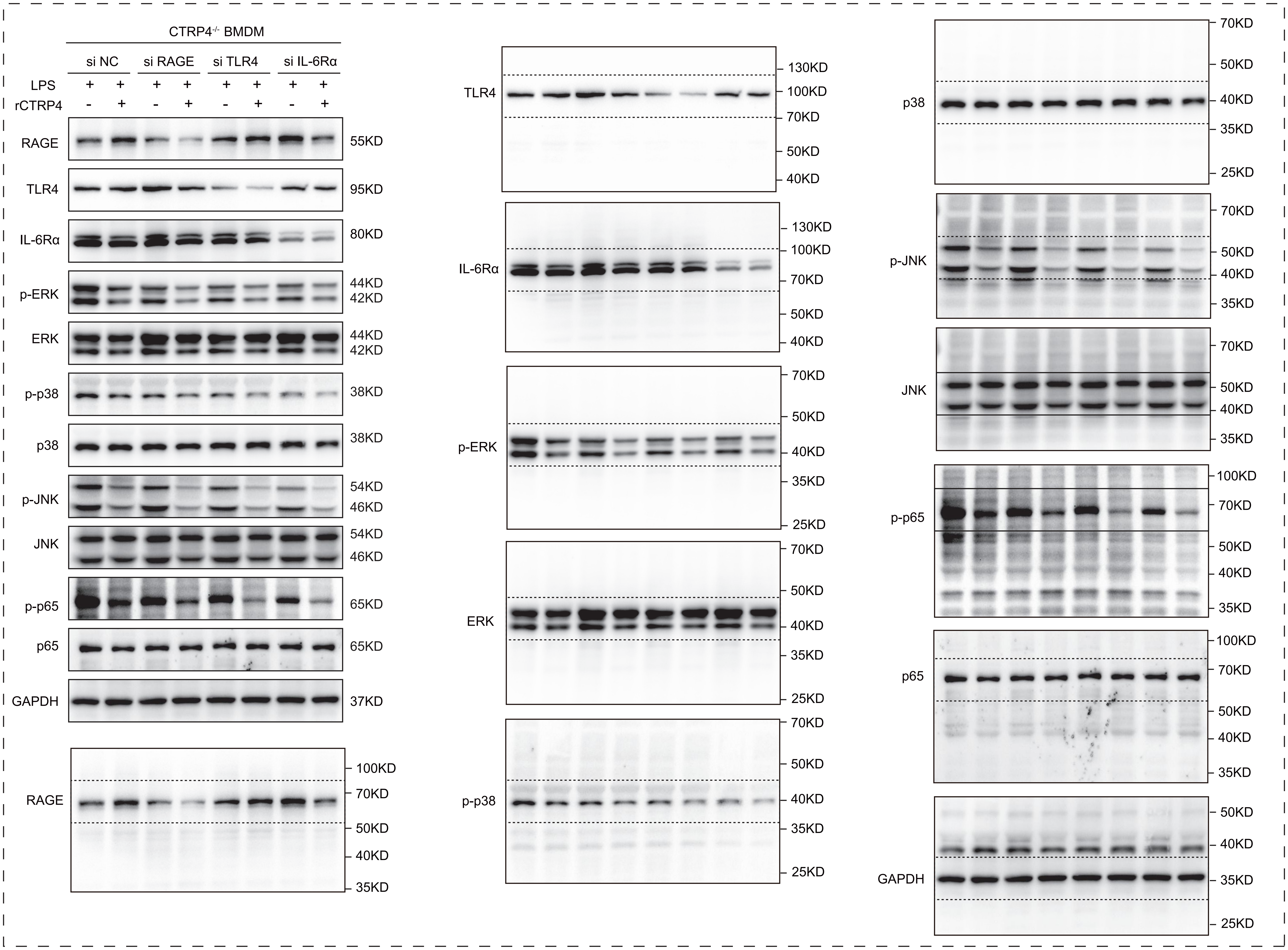

Full unedited gel of immunoblots (6/7)

Figure S8B

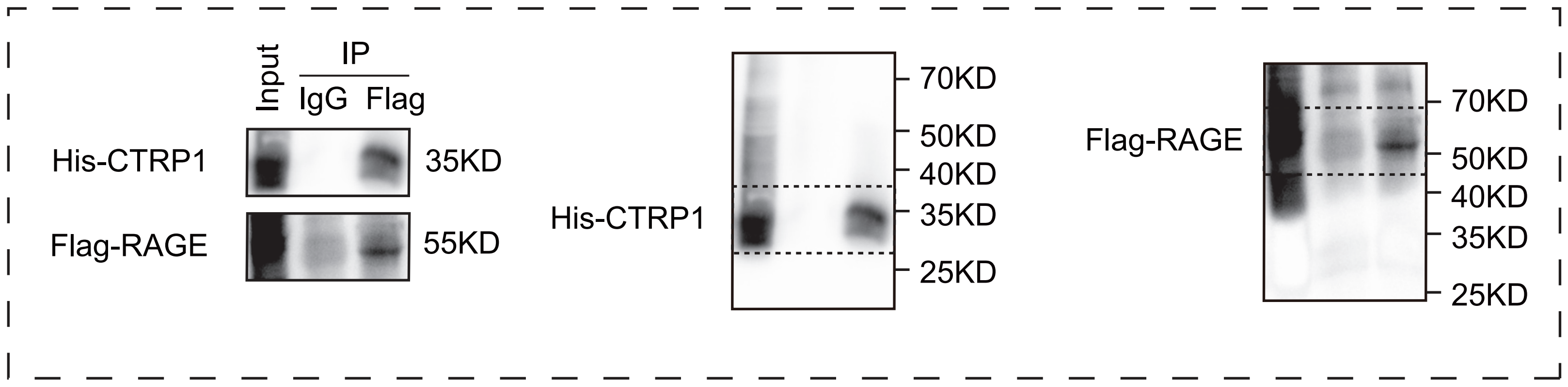

Figure S8C

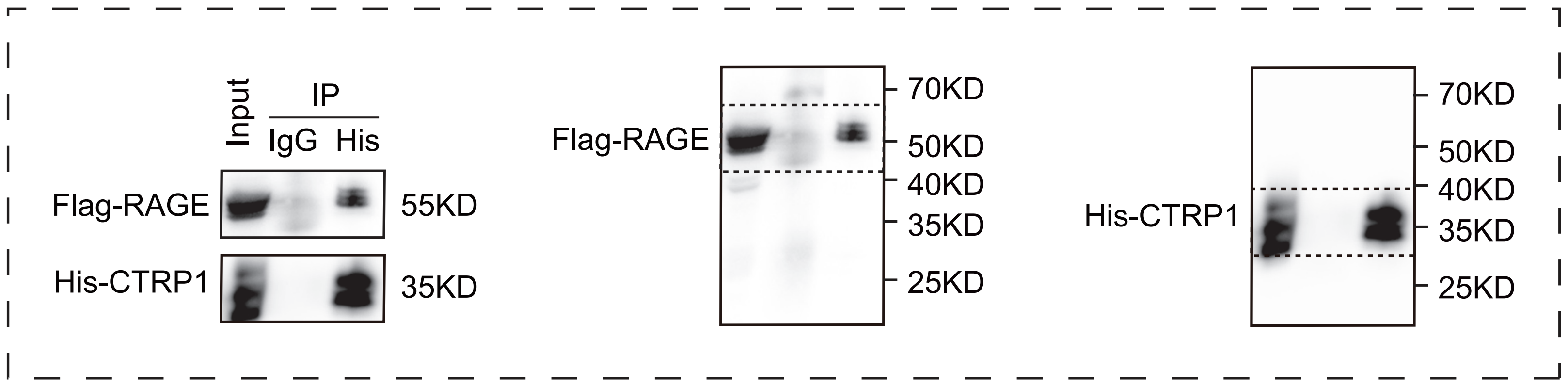

Figure S8D

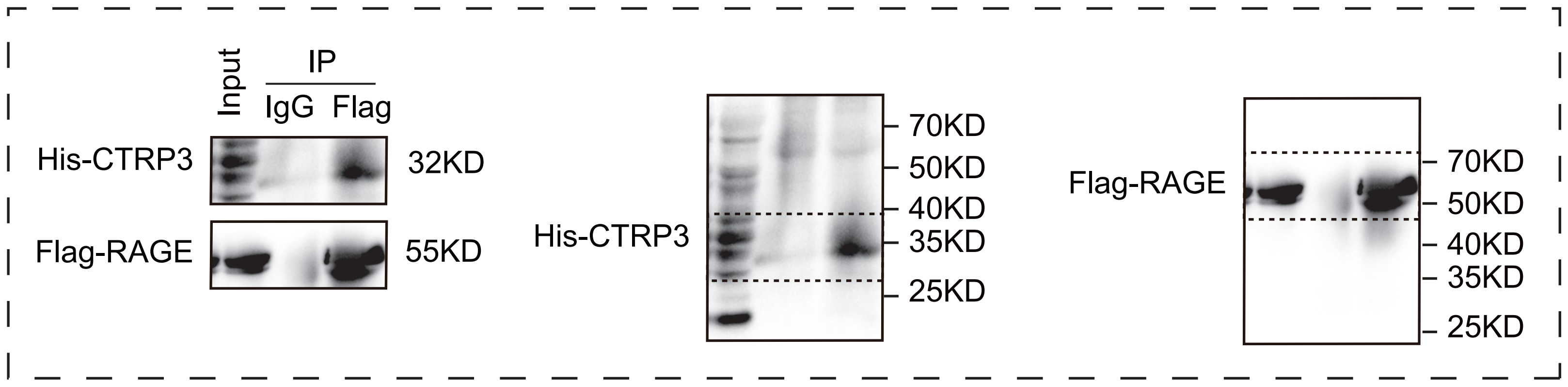

Figure S8E

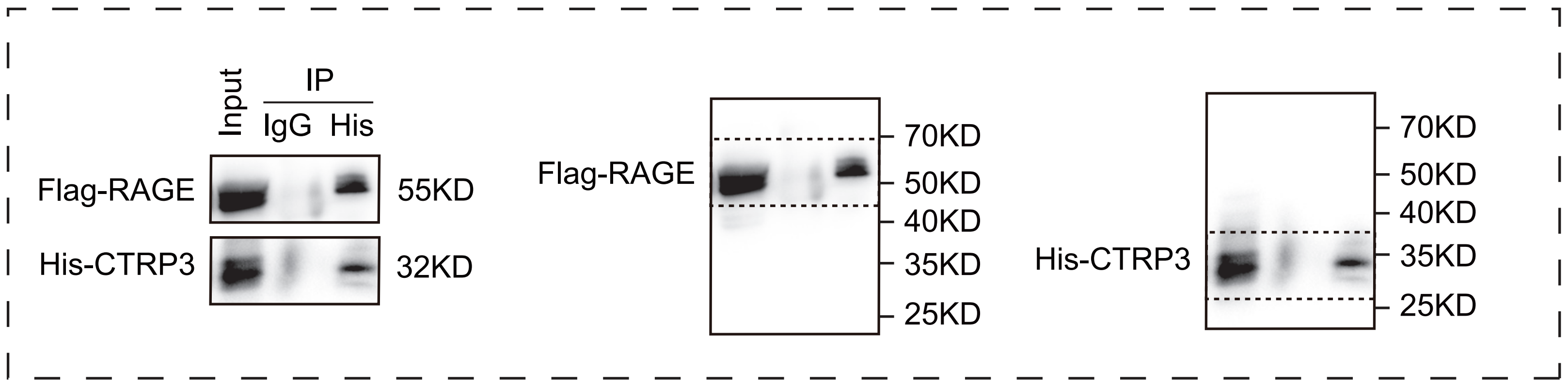

Figure S8F

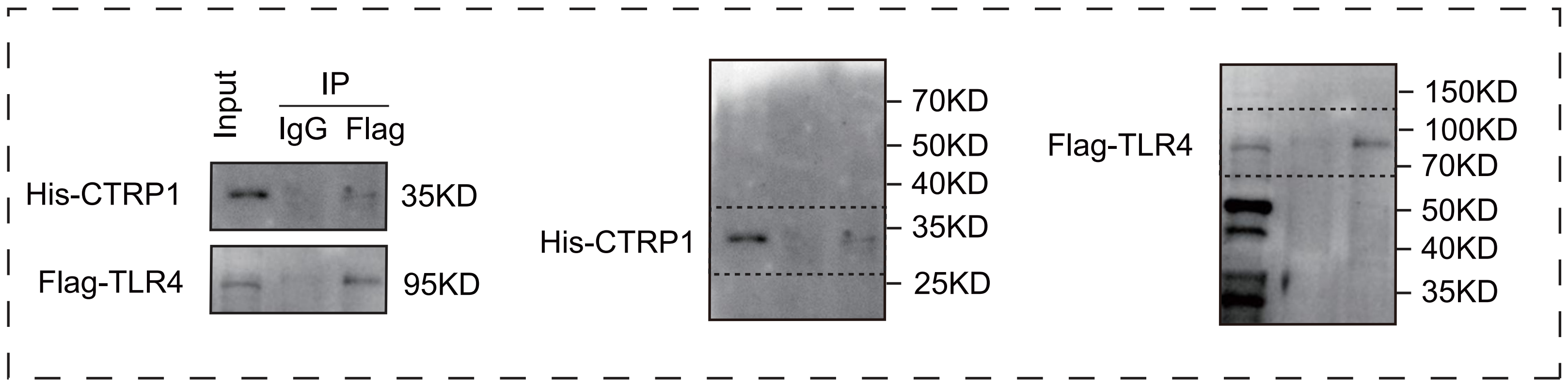

Figure S8G

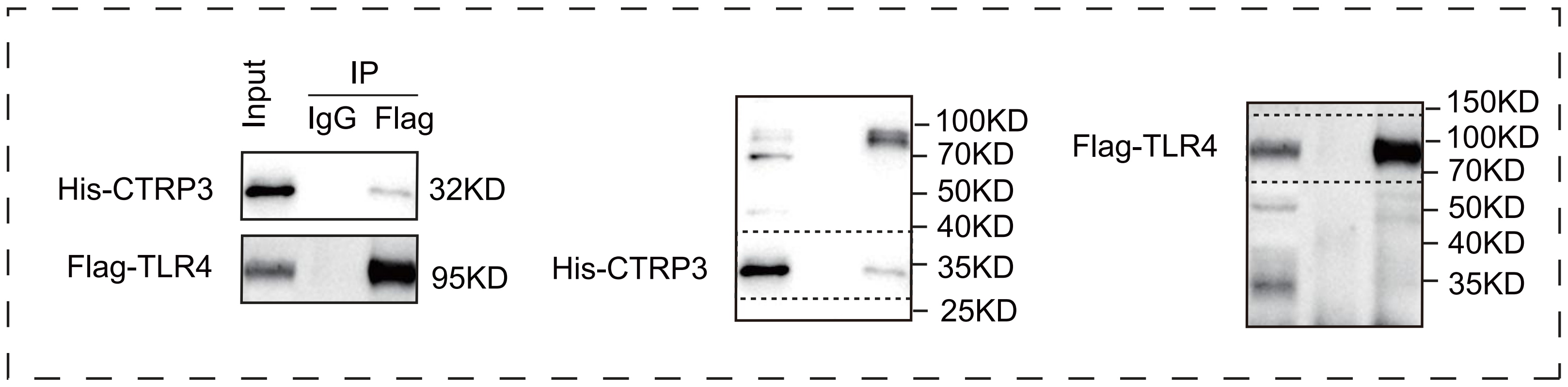

Figure S9A

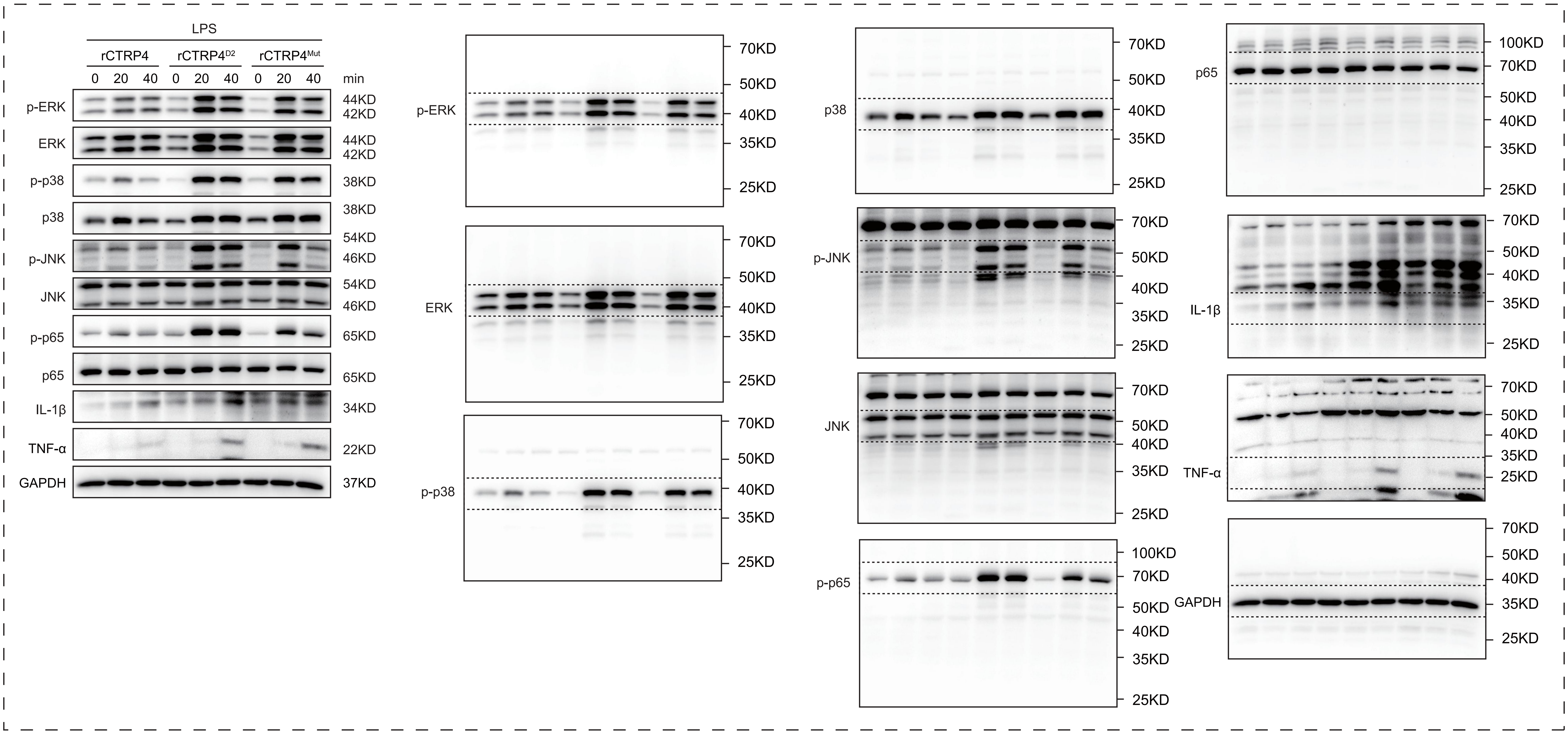

Full unedited gel of immunoblots (7/7)

Figure S10A

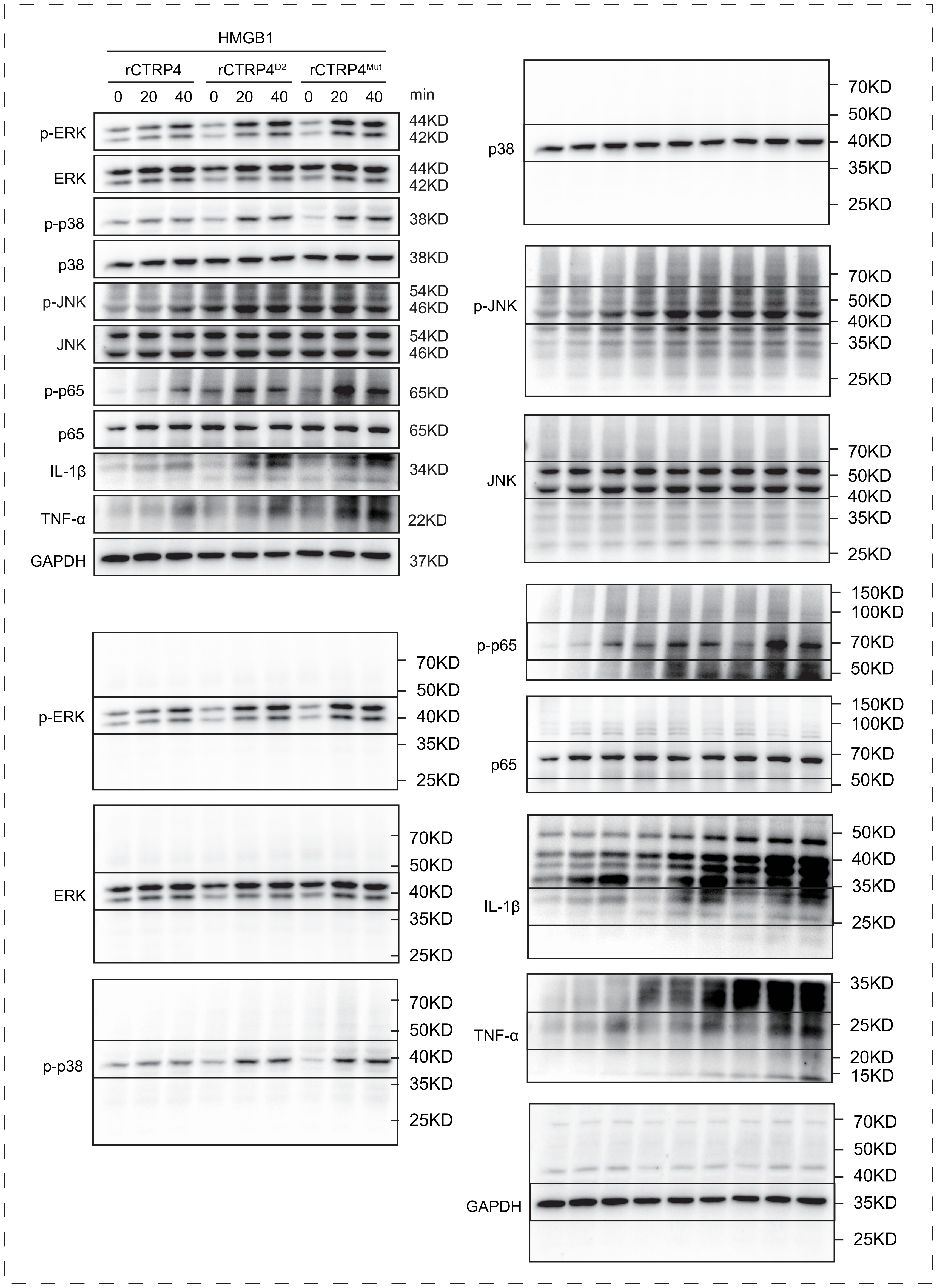

Figure S11A

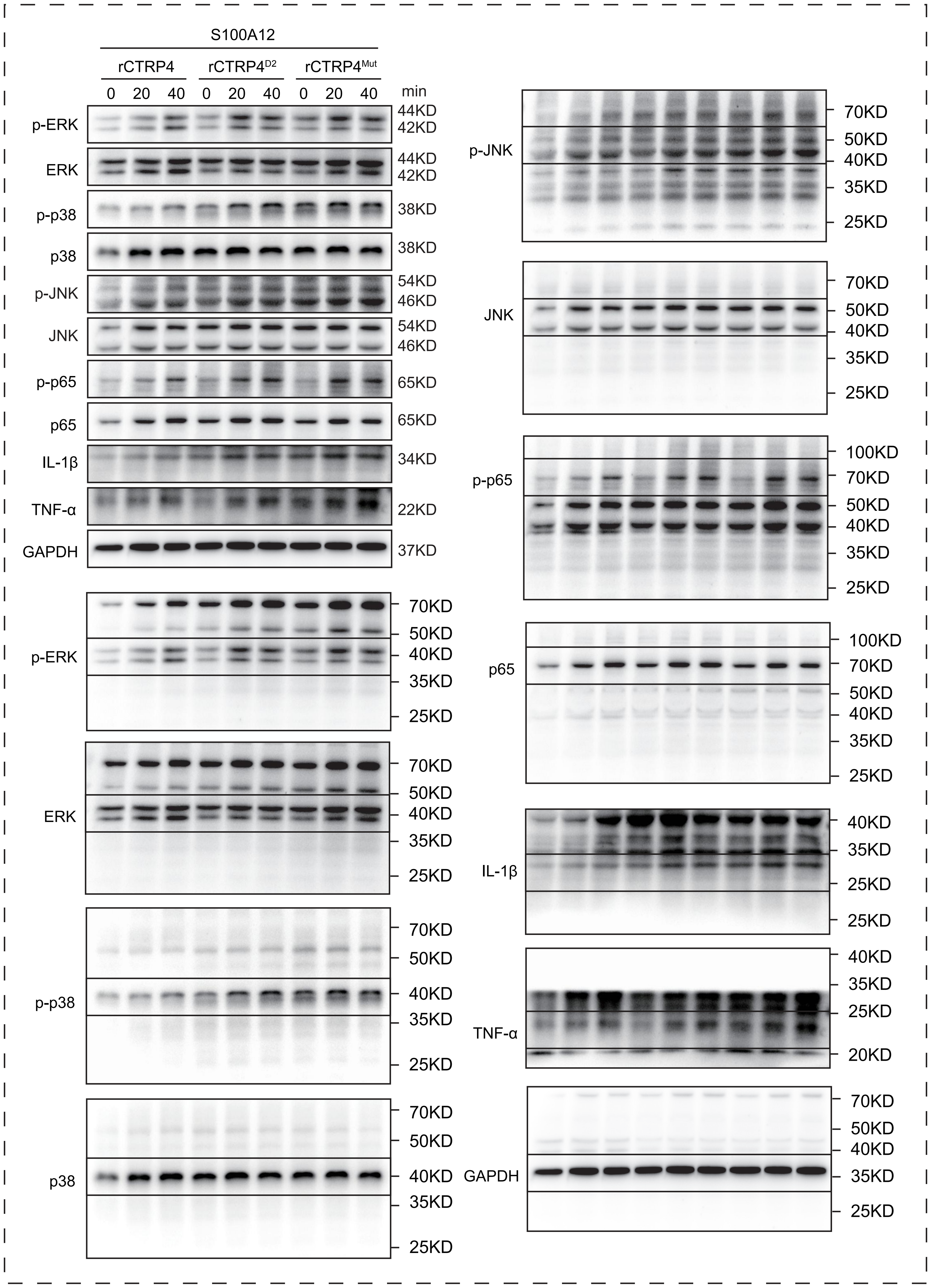

Figure S12A

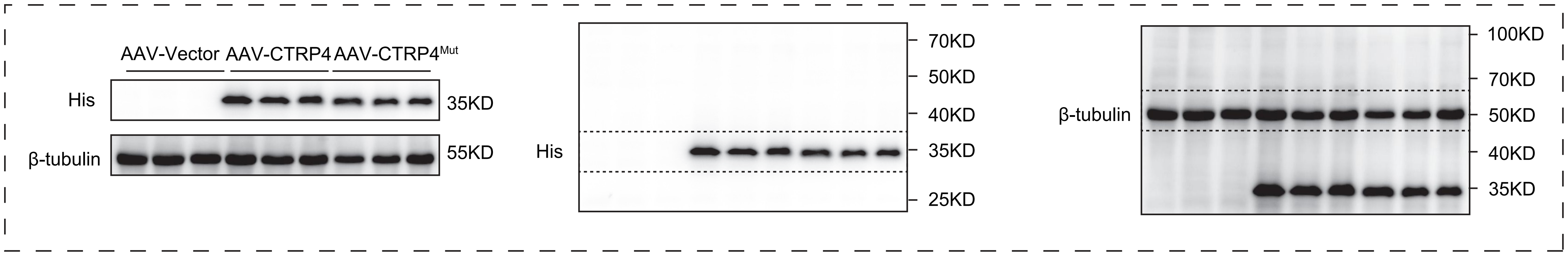

Supplement: Supplementary file 3 — Supporting Information [file CTM2-16-e70624-s001.pdf]
